# Prospective Validation of an Electronic Health Record–Based, Real-Time Suicide Risk Model

**DOI:** 10.1001/jamanetworkopen.2021.1428

**Published:** 2021-03-12

**Authors:** Colin G. Walsh, Kevin B. Johnson, Michael Ripperger, Sarah Sperry, Joyce Harris, Nathaniel Clark, Elliot Fielstein, Laurie Novak, Katelyn Robinson, William W. Stead

**Affiliations:** 1 Department of Biomedical Informatics, Vanderbilt University Medical Center, Nashville, Tennessee; 2 Department of Medicine, Vanderbilt University Medical Center, Nashville, Tennessee; 3 Department of Psychiatry and Behavioral Sciences, Vanderbilt University Medical Center, Nashville, Tennessee; 4 Department of Pediatrics, Vanderbilt University Medical Center, Nashville, Tennessee

## Abstract

**Importance:**

Numerous prognostic models of suicide risk have been published, but few have been implemented outside of integrated managed care systems.

**Objective:**

To evaluate performance of a suicide attempt risk prediction model implemented in a vendor-supplied electronic health record to predict subsequent (1) suicidal ideation and (2) suicide attempt.

**Design, Setting, and Participants:**

This observational cohort study evaluated implementation of a suicide attempt prediction model in live clinical systems without alerting. The cohort comprised patients seen for any reason in adult inpatient, emergency department, and ambulatory surgery settings at an academic medical center in the mid-South from June 2019 to April 2020.

**Main Outcomes and Measures:**

Primary measures assessed external, prospective, and concurrent validity. Manual medical record validation of coded suicide attempts confirmed incident behaviors with intent to die. Subgroup analyses were performed based on demographic characteristics, relevant clinical context/setting, and presence or absence of universal screening. Performance was evaluated using discrimination (number needed to screen, C statistics, positive/negative predictive values) and calibration (Spiegelhalter *z* statistic). Recalibration was performed with logistic calibration.

**Results:**

The system generated 115 905 predictions for 77 973 patients (42 490 [54%] men, 35 404 [45%] women, 60 586 [78%] White, 12 620 [16%] Black). Numbers needed to screen in highest risk quantiles were 23 and 271 for suicidal ideation and attempt, respectively. Performance was maintained across demographic subgroups. Numbers needed to screen for suicide attempt by sex were 256 for men and 323 for women; and by race: 373, 176, and 407 for White, Black, and non-White/non-Black patients, respectively. Model C statistics were, across the health system: 0.836 (95% CI, 0.836-0.837); adult hospital: 0.77 (95% CI, 0.77-0.772); emergency department: 0.778 (95% CI, 0.777-0.778); psychiatry inpatient settings: 0.634 (95% CI, 0.633-0.636). Predictions were initially miscalibrated (Spiegelhalter *z* = −3.1; *P* = .001) with improvement after recalibration (Spiegelhalter *z* = 1.1; *P* = .26).

**Conclusions and Relevance:**

In this study, this real-time predictive model of suicide attempt risk showed reasonable numbers needed to screen in nonpsychiatric specialty settings in a large clinical system. Assuming that research-valid models will translate without performing this type of analysis risks inaccuracy in clinical practice, misclassification of risk, wasted effort, and missed opportunity to correct and prevent such problems. The next step is careful pairing with low-cost, low-harm preventive strategies in a pragmatic trial of effectiveness in preventing future suicidality.

## Key Points

**Question:**

How well do electronic health record–based suicide risk models perform in the clinical setting, and is performance generalizable?

**Findings:**

This cohort study of 30-day suicide attempt risk among 77 973 patients showed good performance in nonpsychiatric clinical settings at scale and in real time. Numbers needed to screen were reasonable for an algorithmic screening test that required no additional data collection or face-to-face screening to calculate.

**Meaning:**

Suicide attempt risk models can be implemented with accurate performance at scale, but performance is not equal in all clinical settings, which requires model recalibration and updating prior to deployment in new settings.

## Teaser

This cohort study evaluates the performance of a suicide attempt risk prediction model implemented in a vendor-supplied electronic health record to predict subsequent suicidal ideation and suicide attempt.

## Introduction

Suicide prevention begins with risk identification and prognostication. The standard of care remains face-to-face screening and routine clinical interaction. Yet rates of suicidal ideation, attempts, and deaths continue to rise internationally despite increased monitoring and intervention efforts.^1^ The coronavirus disease 2019 (COVID-19) pandemic exacerbated contributing factors for suicide and will continue to do so in the post–COVID-19 era.^2,3,4^ Numerous prognostic models of suicide risk have been published.^5^ Few have been implemented into real-world clinical systems outside of integrated managed care settings.^5,6,7^ In some settings, universal screening might reduce risk of downstream suicidality.^8^ But in-person screening takes time and attention and can be conducted with variable quality.^9^ Concealed distress also subverts risk identification in face-to-face screening.^10^ Furthermore, those at risk might not be identified despite health care encounters as recently as the day they die from suicide.^11,12,13^

Linking scalable, automated risk prognostication with real-world clinical processes might improve suicide prevention.^14^ The most prominent example of an operational suicide risk prediction with implemented prevention is REACH VET (Recovery Engagement and Coordination for Health—Veterans Enhanced Treatment) from the Veterans Health Administration.^6^ Similarly, Army STARRS (Study to Assess Risk and Resilience in Servicemembers) demonstrated algorithmic potential in active duty service members.^15,16^ A number of groups, including ours, have published modeling studies for civilians both nationally (eg, the Mental Health Research Network)^5,17,18,19,20^ and internationally.^21^ A recent brief report^7^ estimated the increased potential workload of a suicide risk prediction model to generate alerts in an integrated managed care setting, Kaiser Permanente. In Europe, linking mobile health and predictive modeling for suicide prevention has been described,^22^ as have predictive modeling studies developed for national and single-payer cohorts.^21,23^

While some risk models rely on face-to-face screening data (eg, the Patient Health Questionnaire–9) to calculate risk,^17^ generating these important predictors relies on existing or changing clinical workflow—a difficult task. In some hospitals, universal screening occurs in the emergency department alone. A model reliant solely on routine, passively collected clinical data, such as medication and diagnostic data, might scale to any clinical setting regardless of screening practices. Few real-world data exist on successes and pitfalls of translating such models into operational clinical systems in the presence or absence of universal screening.^7^

Like any prognostic test, such as radiographic imaging and laboratory studies, electronic health record (EHR)–based risk models serve as an additional data point for clinical decision-making. When linked to guideline-informed and evidence-based education along with actionable, user-centered decision support, they might improve provision of suicide prevention. Such systems might prompt care outside of routine health encounters, eg, a prioritized telephone call to a high-risk patient who missed an appointment or guidance on assessing means to a primary care clinician who does not do so regularly. Ideally, these systems would improve quality of care while reducing burden on clinicians to respond appropriately at the right times.

Part of a larger technology-enabled suicide prevention program, our work applied the multiphase framework for action-informed artificial intelligence^24^ to suicide attempt prognostication. We completed phases 1 and 2 in initial model development^20^ followed by phase 3, replicative studies.^18,25^ The fourth phase includes design, usability, and feasibility testing for the operational platform before effectiveness testing and practice improvement in the final phase.

We evaluate prospectively the real-time EHR risk prediction platform here (fourth phase) to answer the question, “How well do EHR-based suicide risk models perform in the clinical setting, and is performance generalizable?” Models that fail to validate at this phase, or those not studied in this fashion prior to implementation, might covertly hinder clinical decision-making. Predictive models might be evaluated similarly to any novel prognostic data point (eg, a laboratory or imaging result).^26^ This validation should account for clinical context, setting, and the presence of universal screening.^8^

## Methods

We studied an observational, prospective cohort of clinical inpatient, emergency department, and ambulatory surgery encounters at a major academic medical center in the mid-South, Vanderbilt University Medical Center (VUMC), from June 2019 to April 2020. Predictions were prompted by the start of routine clinical visits in the EHR. Because model validity was untested outside of research systems,^27^ model predictions did not trigger EHR alerts or decision support.

The VUMC Institutional Review Board approved 2 protocols with waiver of consent given the infeasibility of consenting these EHR-driven analyses across a health system. Only clinical production-grade systems were used to protect privacy and demonstrate feasibility. This study followed the Strengthening the Reporting of Observational Studies in Epidemiology (STROBE) reporting guidelines.^28^

### Study Outcomes

In this study, the predictive model trained on suicide attempt risk was used to predict both suicide attempt (primary) and suicidal ideation (secondary) within 30 days of discharge.^25^ Outcomes were ascertained via reference codes (*International Classification of Diseases, Tenth Revision, Clinical Modification [ICD-10-CM]*^29^; eTable in the Supplement). Because we and others have shown imperfect ascertainment from *ICD* codes for suicide attempt,^20,30^ our team (C.G.W., J.H., S.S.) manually reviewed coded suicide attempts and verified suicidal behaviors with intent to die.

Our previously published approach was internally valid at multiple time points (eg, 30 days vs 90 days).^20^ Thirty-day outcomes were selected as the prediction target with input from behavioral health experts involved in local suicide prevention.

### Inclusion/Exclusion Criteria

We included all adult patients seen in inpatient, emergency department, and ambulatory surgery settings. Individuals with death dates in the Social Security Death Index were right-censored if deaths occurred within 30 days of discharge. Cause-of-death data were not available across the enterprise, so deaths from suicide were not included as prediction targets.

### Implementation

Full modeling details have been published^20^ and are in the eMethods in the Supplement. Briefly, we trained random forests, a nonparametric ensemble machine learning algorithm, on a heterogeneous, retrospective group of adult cases and controls prior to 2017 stored in a deidentified research repository, the Synthetic Derivative.^31^ These models were validated with a variant of bootstrapping with optimism adjustment, in which each bootstrap iteration was tested against a true holdout set to lessen overfitting.^32^ Model performance in training to predict suicide attempt within 30 days showed area under the receiver operating characteristic curve (AUROC) of 0.9 (95% CI, 0.9-0.91) on a deidentified data set of 3250 cases of manually validated suicide attempts and 12 695 adults with no history of suicide attempt.^20^

Predictors included the following:

- Demographic data (age, sex, race)
- Diagnostic data grouped to Centers for Medicare & Medicaid Services Hierarchical Condition Categories (HCC) (eg, schizophrenia-related *ICD* codes mapped to HCC 57)
- Medication data grouped to the Anatomic Therapeutic Classification, level IV (eg, citalopram N06AB04 [level V] maps to Selective Serotonin Reuptake Inhibitors N06AB [level IV])
- Past health care utilization (counts of inpatient, emergency department, and ambulatory surgery visits over the preceding 5 years)
- Area Deprivation Indices^33^ by patient zip code

### Real-Time Prediction

At registration for inpatient, emergency department, or ambulatory surgery visits, the modeling pipeline used 5 years of historical data to build a vector of predictors. Preliminary analyses showed the 5-year lookback window to perform similarly to models using all historical data. The predictive model then generated a probability of subsequent suicide attempt in 30 days. Here, we validate that probability to predict encounters for suicide attempt or suicidal ideation in the subsequent 30 days.

### Recalibration

Calibration measures how well predicted probabilities reflect real-world outcome (eg, a 1% risk of suicide attempt means 1 of 100 similar individuals from that population should have the outcome). Miscalibrated models hamper clinical decision-making.^34^

To enrich signal, the research-grade model^20^ was trained with a sample of the larger population, increasing potential miscalibration. We anticipated miscalibration and corrected it with logistic calibration,^35,36^ a univariate logistic regression model with uncalibrated predictions trained on outcomes from June to October to recalibrate predictions from November to April.

### Evaluation

Performance evaluation included discrimination: AUROC, sensitivity, specificity, positive predictive value (PPV), risk concentration; calibration: calibration slope and intercept, Spiegelhalter *z* statistic (*P* > .05 indicates the model is well calibrated^37^); and usefulness: number needed to screen (NNS, the reciprocal of PPV). Evaluation accounted for the presence or absence of universal screening. To generate CIs and analyze sensitivity to temporal length of EHRs, we varied the minimum length of medical records per performance analysis from all medical records (ie, including records for new patients up to medical records at least 2 years in length). Statistical analyses were conducted in Python, version 3.7 (Python Software Foundation), and in R, version 3.6.1 (R Foundation).

## Results

The study included 115 905 predictions for 77 973 patients (42 490 [54%] men, 35 404 [45%] women, 60 586 [78%] White, 12 620 [16%] Black) over 296 days, approximately 392 predictions per day (Table 1). Our analysis right-censored 1326 patients for all-cause death within 30 days of the preceding encounter. Because patients might be directly admitted without emergency department care, the subtotals per setting sum to greater than enterprisewide totals.

**Table 1.** Baseline Characteristics

Care site	Total	Race					Sex			Age, median, y	Utilization			
		White	Black	Asian	Unknown	Other	Male	Female	Unknown		Median encounters per month	Length of stay, d		Medical record length, median, y
												Median	Mean	
Medical center wide	77 973	60 586	12 620	1454	1233	2080	42 490	35 404	79	52.0	10 923	0.3	1.9	9.0
Behavioral health	2905	2278	497	55	53	23	1532	1373	0	37.0	317	0.3	1.6	5.2
Emergency department	33 235	23 650	7409	646	387	1143	15 862	17 305	68	46.7	3551	0.3	2	5.3
Adult hospital	46 389	38 047	5848	850	678	966	20 540	25 841	8	57.1	6390	2.3	4.5	5.2

Recorded outcomes numbered 129 suicide attempts across 85 individuals (sex: 39 men [46%], 46 women [54%]; race: 64 White [75%]; 18 Black [21%], 3 non-White/non-Black [4%]; 23 repeat attempters) and 946 encounters for suicidal ideation across 395 individuals (sex: 222 men [56%], 156 women [39%]; race: 287 White [73%], 78 Black [20%], 30 non-White/non-Black [8%]; 170 repeat ideators). Manual medical record review of coded suicide attempts had PPV greater than 0.9 in *ICD-10-CM* with an interrater agreement κ of 1, notably higher than the PPV of 0.58 for *ICD-9* in a medical record validation of 5543 medical records in prior work at VUMC.^20^

### Model Performance

Cohort criteria affect model performance, as we and others have shown.^38^ Analyses considered duration of EHRs per patient, clinical settings (eg, inpatient vs emergency department), and universal screening. Demographic characteristics of sex (not gender, which lacks reliable identification in most EHRs) and race were also considered. Performance by length of EHR is shown in aggregate for each outcome (Table 2).

**Table 2.** Prospective Electronic Health Record (EHR) Validation by Length of Individual EHRs, April 2019 to June 2020

Care Site	AUROC (95% CI)	Brier (95% CI)	Spiegelhalter *z* statistic (95% CI)	Spiegelhalter *z*, *P* value (95% CI)	Calibration slope (95% CI)	Calibration intercept (95% CI)
**Suicidal ideation**						
Medical center wide	0.836 (0.836 to 0.837)	0.009 (0.009 to 0.009)	−1.362 (−1.592 to −1.132)	.29 (.25 to .33)	0.273 (0.27 to 0.276)	−2.974 (−2.994 to −2.953)
Emergency department	0.777 (0.776 to 0.778)	0.012 (0.012 to 0.012)	−2.735 (−2.851 to −2.618)	.01 (.01 to .01)	0.342 (0.334 to 0.351)	−2.605 (−2.643 to −2.568)
Adult hospital	0.77 (0.769 to 0.772)	0.002 (0.002 to 0.002)	−10.632 (−10.876 to −10.387)	<.001 (<.001 to <.001)	0.08 (0.079 to 0.08)	−5.432 (−5.452 to −5.412)
Behavioral health	0.634 (0.633 to 0.636)	0.109 (0.108 to 0.109)	27.305 (27.066 to 27.544)	<.001 (<.001 to <.001)	0.075 (0.073 to 0.077)	−1.68 (−1.69 to −1.67)
**Suicide attempt**						
Medical center wide	0.797 (0.796 to 0.798)	0.001 (0.001 to 0.001)	−24.683 (−24.933 to −24.433)	<.001 (<.001 to <.001)	0.189 (0.186 to 0.191)	−5.492 (−5.499 to −5.485)
Emergency department	0.7 (0.699 to 0.7)	0.002 (0.002 to 0.002)	−18.235 (−18.373 to −18.097)	<.001 (<.001 to <.001)	0.113 (0.112 to 0.113)	−5.788 (−5.793 to −5.784)
Adult hospital	0.842 (0.841 to 0.842)	0.001 (0.001 to 0.001)	−14.828 (−15.05 to −14.605)	<.001 (<.001 to <.001)	0.142 (0.141 to 0.142)	−6.462 (−6.48 to −6.444)
Behavioral health	0.544 (0.539 to 0.548)	0.011 (0.011 to 0.011)	−5.539 (−5.567 to −5.511)	<.001 (<.001 to <.001)	0.175 (0.167 to 0.183)	−3.882 (−3.914 to −3.85)

Abbreviation: AUROC, area under the receiver operating characteristic curve.

### Risk Concentration and NNS

**Figure 1.**
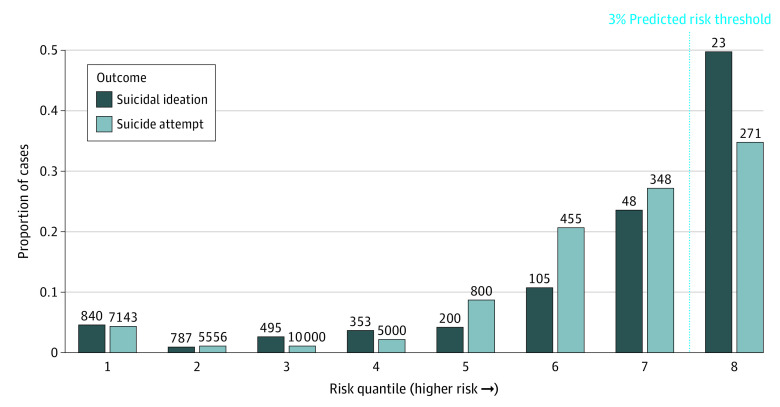
Risk Concentration by Outcome Values above each bar indicate the number needed to screen.

Risk concentration plots for all encounters are shown (Figure 1) with NNS, the reciprocal of PPV, per quantile. The highest risk quantiles have an NNS of 23 and 271 for suicidal ideation and suicide attempt, respectively.

### Evaluation by Status of Universal Screening

Metrics by predicted risk quantile are shown for suicide attempt risk (Table 3). In settings with universal screening, the lowest risk quantile (n = 6795) with predicted risk threshold near 0 had a PPV of 0.1% for suicidal ideation and approximately 0 for suicide attempt. The highest risk quantile (n = 5457) above a threshold of 3.2% had a PPV of 3% for suicidal ideation and 0.3% for suicide attempt.

**Table 3.** Predictive Metrics by Risk Bin and Presence or Absence of Screening

Risk bin	Prediction ranges	No. in bin	No. of cases	PPV	NNS
**Settings without universal screening**					
1	0	23 589	2	8.00E-05	12 500
2	0.000006-0.0005	2842	0	0	NA
3	0.0005-0.002	6894	0	0	NA
4	0.002-0.004	6376	1	0.00016	6250
5	0.004-0.006	6103	1	0.00016	6250
6	0.006-0.01	6582	8	0.00122	820
7	0.01-0.02	6495	17	0.00262	382
8	0.02-0.24	6517	29	0.00445	225
**Universal screening settings**					
1	0	6795	2	0.00029	3448
2	0.000008-0.0002	2752	1	0.00036	2778
3	0.0002-0.0004	3078	0	0	NA
4	0.0004-0.0007	3167	4	0.00126	794
5	0.0007-0.015	3153	7	0.00222	450
6	0.015-0.019	3166	10	0.00316	316
7	0.019-0.03	3171	4	0.00126	794
8	0.03-0.04	3115	10	0.00321	312
9	0.04-0.36	3155	7	0.00222	450

Abbreviations: NA, not applicable; NNS, number needed to screen; PPV, positive predictive value.

In settings without universal screening, the highest risk quantile (n = 4220) above a threshold of 3.2% had a PPV of 4.3% for suicidal ideation and 0.4% for suicide attempt. The lowest risk quantile (n = 23 589) of predicted risk near 0 had a PPV of 0.1% for suicidal ideation and 0 for suicide attempt.

### Risk Prediction Performance by Demographic Subgroup

The NNS for suicide attempt in the highest risk quantiles for men and women in the medical center–wide cohort were 256 and 323, respectively. By race, as coded in the EHR (White, Black), the NNS was 373 for White patients, 176 for Black patients, and 407 for non-White and non-Black patients.

### Calibration

In the first 5 months, predictions were miscalibrated (Spiegelhalter *z* = −3.1; *P* = .001). We applied logistic recalibration using those first 5 months, with improved calibration (Spiegelhalter *z* = 1.1; *P* = .26) in the subsequent 5-month study period.

## Discussion

This study validated performance of a published suicide attempt risk model^20^ using real-time clinical prediction in the background of a vendor-supplied EHR. Primary findings include accuracy at scale regardless of face-to-face screening in nonpsychiatric settings. We note feasible NNS in the highest predicted risk quantiles with potential for reduced screening workload for those at lowest risk. Overall performance was not sensitive to temporal length of EHRs. The decision of minimum length of EHR to display an alert or prediction for an individual patient, however, will be the subject of future decision support testing.

**Figure 2.**
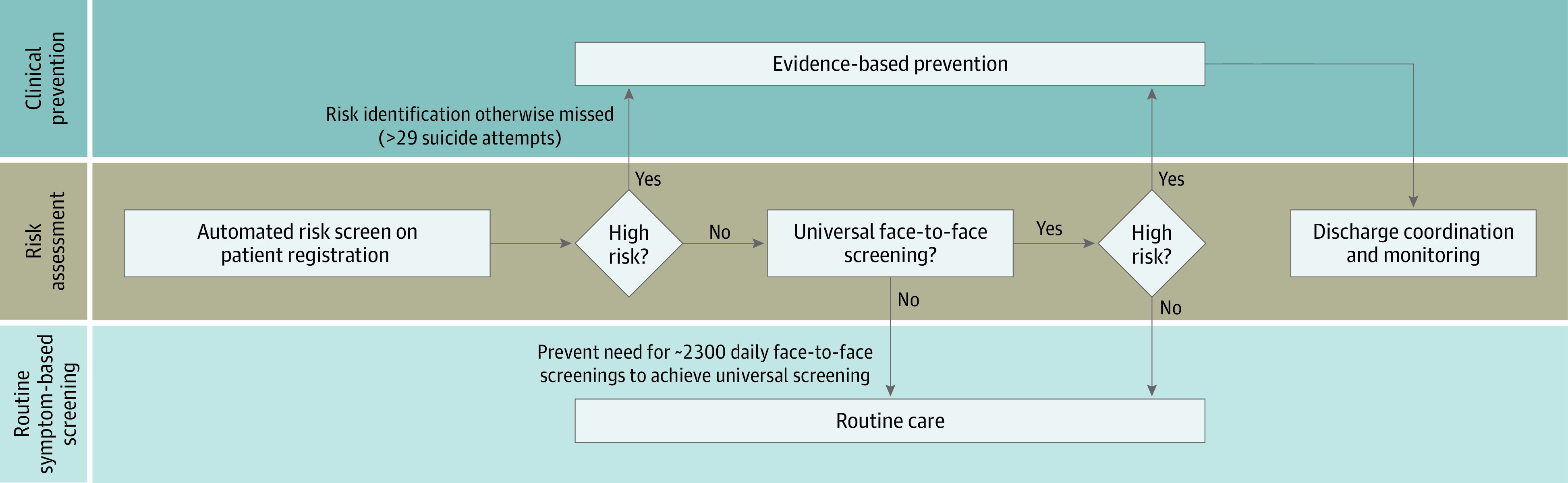
Artificial Intelligence–Enabled Suicide Screening Protocol

The potential implications of this work influence screening practices, clinical decision-making, and care coordination. Regarding screening, both false negatives and false positives have been considered weaknesses of suicide-focused risk models in systematic review.^5^ Here, we note very low false-negative rates in the lowest risk tiers both within (0.02%) and without (0.008%) universal screening settings (Table 3). Assuming that face-to-face screening takes, on average, 1 minute to conduct, automated screening for the lowest quantile alone would release 50 hours of clinician time per month. Regarding false positives, the NNS of 271 was feasible in the highest risk group. Suicidal ideation, even more common, had a better NNS of 23. For context, NNS was 418 for screening for dyslipidemia to prevent cardiac death when it was introduced.^39^ The present study provides further evidence that current models might be best suited to direct prevention to suicidal ideation and attempts—more common yet still in the causal pathway for death from suicide.^5^ A representative screening protocol is shown in Figure 2.

Regarding clinical decision-making, this study suggests clinical utility in identifying those who might not otherwise be assessed for new symptoms, symptomatic worsening, or life stressors not captured in the EHR. Moreover, linking automated risk stratification with evidence-based education on imminent risk, means assessment, and appropriate clinical triage might prove impactful even in the setting of low PPV.^40^

Regarding care coordination, risk models of rare outcomes enable longitudinal monitoring for those who might be at longer-term risk of attempts, (eg, 1-2 years, not 30 days).^40^ Current care coordination in many systems relies on manually curated patient tracking and local workflows by individual clinics or clinicians. Automated risk stratification with decision support might ameliorate challenges such as responding to messages from patients unfamiliar to the nurse or covering clinician, prompting telephone calls to those identified at risk who miss scheduled appointments, and facilitating coordinated care across disparate clinical departments.

A useful model must be well calibrated to reflect reality. Published models often originate from data sets that sample from a larger population to reduce case imbalance given rare outcomes, such as suicide attempts. Once trained, such models risk poor calibration where real-world outcome prevalence does not reflect research. Our risk model is one such example. Calibration of our implemented model was improved with facile logistic recalibration using earlier data to recalibrate later predictions. More sophisticated means of diagnosing and correcting miscalibration and drift merit consideration.^41,42,43^

One model does not fit all. Model performance was lower in psychiatric compared with nonpsychiatric settings. This model was trained on a heterogeneous mix of medical records prior to 2017. First, prevalent mental health–related risk factors in psychiatric settings might worsen model discrimination compared with the training sample. Second, outcomes were rare in those settings, and cohorts were concomitantly smaller. Third, because psychiatric care is likely to address suicidality, care in those settings confounds pure prognostic accuracy. Such therapeutic differences might be well suited to counterfactual prediction in the future.^44^ Future work should include development and validation of site-specific predictive models—or models that will be “site aware” in deployment.

Without attention to these differences and analyses conducted here, intervention might be linked to misspecified models. Moreover, it becomes difficult to assess pure model performance once an intervention is prompted by it. Future iterations of these models (1) might be updated based on site-specific cohorts to improve performance and (2) should include the interventions available to prevent suicide within models themselves to prevent model drift even when the care delivered is accomplishing its intended purpose.^27^

Strengths of this study include a large, real-world vendor-supplied EHR setting. It incorporates prospective validation on natural cohorts of individuals receiving routine care over the study period. The study included visits across the breadth of a major academic medical center, which improves generalizability. These results complete only part of the fourth phase of action-informed artificial intelligence^24^ to help prevent suicide. We have designed our models with usability and feasibility in mind, but these have not yet been tested. Our modeling requires no additional screening (eg, the Patient Health Questionnaire–9 or Columbia Suicide Severity Rating Scale), although future versions might incorporate them to improve risk prognostication. Yet, impact will not be achieved without careful attention to the people and clinical processes to leverage these predictions to prevent suicide.

### Limitations

Limitations of this work include a single-center study with low outcome prevalence, particularly for suicide attempt. Predictors included in this model were chosen to optimize scalability and potential generalizability. They rely on structured data ubiquitous in EHRs (diagnostic codes, medications, past utilization, demographic characteristics). However, they also limit the model’s ability to predict suicide attempt risk by failing to capture important predictors recorded in unstructured free text notes, for example, or outside the EHR. Implemented risk models were initially trained on a noncomprehensive subsample of the medical center population. Ascertainment is limited to care at a single medical center, so events occurring at external health systems were not captured. However, this bias is conservative for model performance analyses; suicide attempts that occur outside the study site are false negatives, far more likely to affect and worsen apparent performance metrics given the low number of cases than the inverse. Deaths from suicide were not ascertained in this study. Future work to improve ascertainment and continuously evaluate these models in production is paramount.

Multiple opportunities to expand this work remain. Better understanding of misclassification of risk will improve model performance and potential impact. Novel means of ascertaining suicidality both in and out of individual health systems through health information exchange—such as that available in the Veterans Health Administration; in large health systems, such as HCA Healthcare, Tenet Healthcare, or Kaiser Permanente; or in states, such as Connecticut^45^—might lead to improved model evaluation and improved performance. Through partnerships such as the Tennessee Department of Health–VUMC Experience,^46^ we are beginning to devise a system that would bridge the current gap preventing ascertainment of death from suicide.

Suicide prevention will not be achieved through a predictive model alone, regardless of its analytic performance. Pragmatic trials to study real-world effectiveness of these predictive models in concert with thoughtful, user-centered clinical decision support remains the path to achieving clinical impact in suicide prevention.

## Conclusions

In this study, implementation of validated predictive models of suicide attempt risk showed reasonable performance at scale and feasible NNS for subsequent suicidal ideation or suicide attempt in a large clinical system. Calibration performance of research-derived models was improved with logistic calibration. Scalable, real-time automated prediction of risk of suicidality through multidisciplinary collaboration is an achievable goal for the growing number of predictive models in this domain. It requires careful pairing with low-cost, low-harm preventive strategies in a pragmatic trial to be evaluated for effectiveness in preventing suicidality in the future.

## Notes

Supplement. **eMethods.** Predictive Modeling Details **eReferences.** **eTable.** Reference Codes From International Classification of Diseases (ICD), Version 10, Clinical Modification
