# Supplementary material for: Prospective Validation of an Electronic Health Record–Based, Real-Time Suicide Risk Model
Source: JAMA Netw Open. 2021 Mar 12;4(3):e211428. doi: 10.1001/jamanetworkopen.2021.1428 (PMC7955273; doi:10.1001/jamanetworkopen.2021.1428)
Supplement: Supplement. — eMethods. Predictive Modeling Details eReferences. eTable. Reference Codes From International Classification of Diseases (ICD), Version 10, Clinical Modification [file jamanetwopen-e211428-s001.pdf]

Supplemental Online Content

Walsh CG, Johnson KB, Ripperger M, et al. Prospective validation of an electronic health record–based, real-time suicide risk model. *JAMA Netw Open*. 2021;4(3):e211428. doi:10.1001/jamanetworkopen.2021.1428

eMethods. Predictive Modeling Details
eReferences.
eTable. Reference Codes From International Classification of Diseases (ICD), Version 10, Clinical Modification

This supplemental material has been provided by the authors to give readers additional information about their work.

© 2021 Walsh CG et al. *JAMA Network Open.*

eMethods. Predictive Modeling Details

Model Training, adapted and abbreviated from Walsh et al, 2017.[1]

Data were drawn from the VUMC Synthetic Derivative (SD), a de-identified electronic health record repository.[2] Candidate charts were identified using self-injury International Classification of Diseases, version 9 (ICD-9) codes (E95x.xx) for all adults in the SD. Cases of suicide attempt were identified through multi-expert chart review on a candidate list of 5543 charts with self-injury codes to identify 3250 adults (aged 18 or older) with expert-validated evidence of self-harm with suicidal intent. A cohort of 12,695 adults with a minimum of three visits to VUMC were drawn from the general population as the control comparison.

Clinical data were preprocessed including demographics (age, coded sex, coded race); clinical diagnoses grouped from individual ICD-9 codes to Center for Medicare and Medicaid Servicers Hierarchical Condition Categories;[3] medications grouped to the Anatomic Therapeutic Classification, level IV;[4] healthcare utilization including counts of inpatient, outpatient, and emergency department visits for each year of the preceding five years; body mass index.

Variables were measured as counts—diagnoses, medications and visits— so missing data were imputed to zeroes if not present. Zip codes were used to calculate Area Deprivation Index[5]. Missing data tallies included zip code in 6% of charts, body mass index was missing in 9.9%, race was missing in 3.6%, and date of birth used to calculate age was missing in 0.7%. Multiple imputation was used to impute missing values in those instances.[6]

Random forests showed superior discrimination performance in identifying suicide attempt risk compared to support vector machines and regression with or without penalization. With tuning parameters of 500 trees per forest and splits of the square root of the number of predictors at each node in the tree, the predictive models used here were trained via optimism adjustment with the bootstrap using 100 bootstraps. To prevent overfitting in testing each bootstrap iteration on the full apparent dataset in a method that also incorporates sampling with replacement (random forests),[7,8] the optimism adjustment validation strategy was as follows:

1) Train and test on the full study dataset to establish apparent (and optimistic) performance.
2) Resample the dataset with replacement and hold-out those cases not selected in resampling as a test set to be used per bootstrap iteration.
3) Train a new random forest on the resampled training set and test on the held-out test set per 100 bootstrap iterations. In traditional optimism adjustment with the bootstrap, the test set per iteration might be the full, apparent dataset, but this strategy can lead to overly optimistic results even after optimism adjustment.
4) Proceed with optimism adjustment using the performance differences per bootstrap and the apparent performance established in step #1.

eReferences

1. Walsh CG, Ribeiro JD, Franklin JC. Predicting risk of suicide attempts over time through machine learning. *Clinical Psychological Science*. 2017;5(3):457–469.

2. Roden D, Pulley J, Basford M, et al. Development of a Large-Scale De-Identified DNA Biobank to Enable Personalized Medicine. *Clinical Pharmacology & Therapeutics*. 2008;84(3):362-369. doi:10.1038/clpt.2008.89

3. Pope GC, Kautter J, Ph D, et al. Risk Adjustment of Medicare Capitation Payments Using the CMS-HCC Model. 2006;25(4):119–141.

4. WHO Collaborating Centre for Drug Statistics Methodology. *Anatomical Therapeutic Chemical (ATC) Classification System*. WHO Collaborating Centre for Drug Statistics Methodology; 2015. http://www.whocc.no/copyright_disclaimer/

5. Singh GK. Area deprivation and widening inequalities in US mortality, 1969-1998. *American journal of public health*. 2003;93(7):1137–43.

6. Buuren S, Groothuis-Oudshoorn K. mice: Multivariate imputation by chained equations in R. *Journal of statistical software*. 2011;45(3). Accessed August 22, 2017. http://doc.utwente.nl/78938/

7. Miao Y, Francisco S, Boscardin WJ, Francisco S, Francisco S. SAS Global Forum 2013: Statistics and Data Analysis. Estimating Harrell ' s Optimism on Predictive Indices Using Bootstrap Samples. Published online 2013:1–12.

8. Efron B, Tibshirani R. *An Introduction to the Bootstrap*. Chapman & Hall; 1993.

© 2021 Walsh CG et al. *JAMA Network Open.*

eTable. Reference Codes From International Classification of Diseases (ICD), Version 10, Clinical Modification

ICD Code	Diagnostic Code Name	ICD Schema
X60	Intentional self-poisoning by and exposure to nonopioid analgesics, antipyretics and antirheumatics	ICD10
X61	Intentional self-poisoning by and exposure to antiepileptic, sedative-hypnotic, antiparkinsonism and psychotropic drugs, not elsewhere classified	ICD10
X62	Intentional self-poisoning by and exposure to narcotics and psychodysleptics [hallucinogens], not elsewhere classified	ICD10
X63	Intentional self-poisoning by and exposure to other drugs acting on the autonomic nervous system	ICD10
X64	Intentional self-poisoning by and exposure to other and unspecified drugs, medicaments and biological substances	ICD10
X65	Intentional self-poisoning by and exposure to alcohol	ICD10
X66	Intentional self-poisoning by and exposure to organic solvents and halogenated hydrocarbons and their vapours	ICD10
X67	Intentional self-poisoning by and exposure to other gases and vapours	ICD10
X68	Intentional self-poisoning by and exposure to pesticides	ICD10
X69	Intentional self-poisoning by and exposure to other and unspecified chemicals and noxious substances	ICD10
X70	Intentional self-harm by hanging, strangulation and suffocation	ICD10
X71	Intentional self-harm by drowning and submersion	ICD10
X72	Intentional self-harm by handgun discharge	ICD10
X73	Intentional self-harm by rifle, shotgun and larger firearm discharge	ICD10
X74	Intentional self-harm by other and unspecified firearm discharge	ICD10
X75	Intentional self-harm by explosive material	ICD10
X76	Intentional self-harm by smoke, fire and flames	ICD10

© 2021 Walsh CG et al. *JAMA Network Open.*

X77	Intentional self-harm by steam, hot vapours and hot objects	ICD10
X78	Intentional self-harm by sharp object	ICD10
X79	Intentional self-harm by blunt object	ICD10
X80	Intentional self-harm by jumping from a high place	ICD10
X81	Intentional self-harm by jumping or lying before moving object	ICD10
X82	Intentional self-harm by crashing of motor vehicle	ICD10
X83	Intentional self-harm by other specified means	ICD10
X84	Intentional self-harm by unspecified means	ICD10
Y87.0	Sequelae of intentional self-harm	ICD10
T14.91	Suicide attempt	ICD10CM
T14.91XA	Suicide attempt, initial encounter	ICD10CM
T14.91XD	Suicide attempt, subsequent encounter	ICD10CM
T14.91XS	Suicide attempt, sequela	ICD10CM
T36.0X2	Poisoning by penicillins, intentional self-harm	ICD10CM
T36.0X2A	Poisoning by penicillins, intentional self-harm, initial encounter	ICD10CM
T36.0X2D	Poisoning by penicillins, intentional self-harm, subsequent encounter	ICD10CM
T36.0X2S	Poisoning by penicillins, intentional self-harm, sequela	ICD10CM
T36.1X2	Poisoning by cephalosporins and other beta-lactam antibiotics, intentional self-harm	ICD10CM
T36.1X2A	Poisoning by cephalosporins and other beta-lactam antibiotics, intentional self-harm, initial encounter	ICD10CM
T36.1X2D	Poisoning by cephalosporins and other beta-lactam antibiotics, intentional self-harm, subsequent encounter	ICD10CM
T36.1X2S	Poisoning by cephalosporins and other beta-lactam antibiotics, intentional self-harm, sequela	ICD10CM

© 2021 Walsh CG et al. *JAMA Network Open.*

T36.2X2	Poisoning by chloramphenicol group, intentional self-harm	ICD10CM
T36.2X2A	Poisoning by chloramphenicol group, intentional self-harm, initial encounter	ICD10CM
T36.2X2D	Poisoning by chloramphenicol group, intentional self-harm, subsequent encounter	ICD10CM
T36.2X2S	Poisoning by chloramphenicol group, intentional self-harm, sequela	ICD10CM
T36.3X2	Poisoning by macrolides, intentional self-harm	ICD10CM
T36.3X2A	Poisoning by macrolides, intentional self-harm, initial encounter	ICD10CM
T36.3X2D	Poisoning by macrolides, intentional self-harm, subsequent encounter	ICD10CM
T36.3X2S	Poisoning by macrolides, intentional self-harm, sequela	ICD10CM
T36.4X2	Poisoning by tetracyclines, intentional self-harm	ICD10CM
T36.4X2A	Poisoning by tetracyclines, intentional self-harm, initial encounter	ICD10CM
T36.4X2D	Poisoning by tetracyclines, intentional self-harm, subsequent encounter	ICD10CM
T36.4X2S	Poisoning by tetracyclines, intentional self-harm, sequela	ICD10CM
T36.5X2	Poisoning by aminoglycosides, intentional self-harm	ICD10CM
T36.5X2A	Poisoning by aminoglycosides, intentional self-harm, initial encounter	ICD10CM
T36.5X2D	Poisoning by aminoglycosides, intentional self-harm, subsequent encounter	ICD10CM
T36.5X2S	Poisoning by aminoglycosides, intentional self-harm, sequela	ICD10CM
T36.6X2	Poisoning by rifampicins, intentional self-harm	ICD10CM
T36.6X2A	Poisoning by rifampicins, intentional self-harm, initial encounter	ICD10CM
T36.6X2D	Poisoning by rifampicins, intentional self-harm, subsequent encounter	ICD10CM
T36.6X2S	Poisoning by rifampicins, intentional self-harm, sequela	ICD10CM
T36.7X2	Poisoning by antifungal antibiotics, systemically used, intentional self-harm	ICD10CM
T36.7X2A	Poisoning by antifungal antibiotics, systemically used, intentional self-harm, initial encounter	ICD10CM

© 2021 Walsh CG et al. *JAMA Network Open.*

T36.7X2D	Poisoning by antifungal antibiotics, systemically used, intentional self-harm, subsequent encounter	ICD10CM
T36.7X2S	Poisoning by antifungal antibiotics, systemically used, intentional self-harm, sequela	ICD10CM
T36.8X2	Poisoning by other systemic antibiotics, intentional self-harm	ICD10CM
T36.8X2A	Poisoning by other systemic antibiotics, intentional self-harm, initial encounter	ICD10CM
T36.8X2D	Poisoning by other systemic antibiotics, intentional self-harm, subsequent encounter	ICD10CM
T36.8X2S	Poisoning by other systemic antibiotics, intentional self-harm, sequela	ICD10CM
T36.92	Poisoning by unspecified systemic antibiotic, intentional self-harm	ICD10CM
T36.92XA	Poisoning by unspecified systemic antibiotic, intentional self-harm, initial encounter	ICD10CM
T36.92XD	Poisoning by unspecified systemic antibiotic, intentional self-harm, subsequent encounter	ICD10CM
T36.92XS	Poisoning by unspecified systemic antibiotic, intentional self-harm, sequela	ICD10CM
T37.0X2	Poisoning by sulfonamides, intentional self-harm	ICD10CM
T37.0X2A	Poisoning by sulfonamides, intentional self-harm, initial encounter	ICD10CM
T37.0X2D	Poisoning by sulfonamides, intentional self-harm, subsequent encounter	ICD10CM
T37.0X2S	Poisoning by sulfonamides, intentional self-harm, sequela	ICD10CM
T37.1X2	Poisoning by antimycobacterial drugs, intentional self-harm	ICD10CM
T37.1X2A	Poisoning by antimycobacterial drugs, intentional self-harm, initial encounter	ICD10CM
T37.1X2D	Poisoning by antimycobacterial drugs, intentional self-harm, subsequent encounter	ICD10CM
T37.1X2S	Poisoning by antimycobacterial drugs, intentional self-harm, sequela	ICD10CM
T37.2X2	Poisoning by antimalarials and drugs acting on other blood protozoa, intentional self-harm	ICD10CM
T37.2X2A	Poisoning by antimalarials and drugs acting on other blood protozoa, intentional self-harm, initial encounter	ICD10CM

© 2021 Walsh CG et al. *JAMA Network Open.*

T37.2X2D	Poisoning by antimalarials and drugs acting on other blood protozoa, intentional self-harm, subsequent encounter	ICD10CM
T37.2X2S	Poisoning by antimalarials and drugs acting on other blood protozoa, intentional self-harm, sequela	ICD10CM
T37.3X2	Poisoning by other antiprotozoal drugs, intentional self-harm	ICD10CM
T37.3X2A	Poisoning by other antiprotozoal drugs, intentional self-harm, initial encounter	ICD10CM
T37.3X2D	Poisoning by other antiprotozoal drugs, intentional self-harm, subsequent encounter	ICD10CM
T37.3X2S	Poisoning by other antiprotozoal drugs, intentional self-harm, sequela	ICD10CM
T37.4X2	Poisoning by anthelminthics, intentional self-harm	ICD10CM
T37.4X2A	Poisoning by anthelminthics, intentional self-harm, initial encounter	ICD10CM
T37.4X2D	Poisoning by anthelminthics, intentional self-harm, subsequent encounter	ICD10CM
T37.4X2S	Poisoning by anthelminthics, intentional self-harm, sequela	ICD10CM
T37.5X2	Poisoning by antiviral drugs, intentional self-harm	ICD10CM
T37.5X2A	Poisoning by antiviral drugs, intentional self-harm, initial encounter	ICD10CM
T37.5X2D	Poisoning by antiviral drugs, intentional self-harm, subsequent encounter	ICD10CM
T37.5X2S	Poisoning by antiviral drugs, intentional self-harm, sequela	ICD10CM
T37.8X2	Poisoning by other specified systemic anti-infectives and antiparasitics, intentional self-harm	ICD10CM
T37.8X2A	Poisoning by other specified systemic anti-infectives and antiparasitics, intentional self-harm, initial encounter	ICD10CM
T37.8X2D	Poisoning by other specified systemic anti-infectives and antiparasitics, intentional self-harm, subsequent encounter	ICD10CM
T37.8X2S	Poisoning by other specified systemic anti-infectives and antiparasitics, intentional self-harm, sequela	ICD10CM
T37.92	Poisoning by unspecified systemic anti-infective and antiparasitics, intentional self-harm	ICD10CM
T37.92XA	Poisoning by unspecified systemic anti-infective and antiparasitics, intentional self-harm, initial encounter	ICD10CM

© 2021 Walsh CG et al. *JAMA Network Open.*

T37.92XD	Poisoning by unspecified systemic anti-infective and antiparasitics, intentional self-harm, subsequent encounter	ICD10CM
T37.92XS	Poisoning by unspecified systemic anti-infective and antiparasitics, intentional self-harm, sequela	ICD10CM
T38.0X2	Poisoning by glucocorticoids and synthetic analogues, intentional self-harm	ICD10CM
T38.0X2A	Poisoning by glucocorticoids and synthetic analogues, intentional self-harm, initial encounter	ICD10CM
T38.0X2D	Poisoning by glucocorticoids and synthetic analogues, intentional self-harm, subsequent encounter	ICD10CM
T38.0X2S	Poisoning by glucocorticoids and synthetic analogues, intentional self-harm, sequela	ICD10CM
T38.1X2	Poisoning by thyroid hormones and substitutes, intentional self-harm	ICD10CM
T38.1X2A	Poisoning by thyroid hormones and substitutes, intentional self-harm, initial encounter	ICD10CM
T38.1X2D	Poisoning by thyroid hormones and substitutes, intentional self-harm, subsequent encounter	ICD10CM
T38.1X2S	Poisoning by thyroid hormones and substitutes, intentional self-harm, sequela	ICD10CM
T38.2X2	Poisoning by antithyroid drugs, intentional self-harm	ICD10CM
T38.2X2A	Poisoning by antithyroid drugs, intentional self-harm, initial encounter	ICD10CM
T38.2X2D	Poisoning by antithyroid drugs, intentional self-harm, subsequent encounter	ICD10CM
T38.2X2S	Poisoning by antithyroid drugs, intentional self-harm, sequela	ICD10CM
T38.3X2	Poisoning by insulin and oral hypoglycemic [antidiabetic] drugs, intentional self-harm	ICD10CM
T38.3X2A	Poisoning by insulin and oral hypoglycemic [antidiabetic] drugs, intentional self-harm, initial encounter	ICD10CM
T38.3X2D	Poisoning by insulin and oral hypoglycemic [antidiabetic] drugs, intentional self-harm, subsequent encounter	ICD10CM
T38.3X2S	Poisoning by insulin and oral hypoglycemic [antidiabetic] drugs, intentional self-harm, sequela	ICD10CM
T38.4X2	Poisoning by oral contraceptives, intentional self-harm	ICD10CM

© 2021 Walsh CG et al. *JAMA Network Open.*

**T38.4X2A**	Poisoning by oral contraceptives, intentional self-harm, initial encounter	ICD10CM
**T38.4X2D**	Poisoning by oral contraceptives, intentional self-harm, subsequent encounter	ICD10CM
**T38.4X2S**	Poisoning by oral contraceptives, intentional self-harm, sequela	ICD10CM
**T38.5X2**	Poisoning by other estrogens and progestogens, intentional self-harm	ICD10CM
**T38.5X2A**	Poisoning by other estrogens and progestogens, intentional self-harm, initial encounter	ICD10CM
**T38.5X2D**	Poisoning by other estrogens and progestogens, intentional self-harm, subsequent encounter	ICD10CM
**T38.5X2S**	Poisoning by other estrogens and progestogens, intentional self-harm, sequela	ICD10CM
**T38.6X2**	Poisoning by antigonadotrophins, antiestrogens, antiandrogens, not elsewhere classified, intentional self-harm	ICD10CM
**T38.6X2A**	Poisoning by antigonadotrophins, antiestrogens, antiandrogens, not elsewhere classified, intentional self-harm, initial encounter	ICD10CM
**T38.6X2D**	Poisoning by antigonadotrophins, antiestrogens, antiandrogens, not elsewhere classified, intentional self-harm, subsequent encounter	ICD10CM
**T38.6X2S**	Poisoning by antigonadotrophins, antiestrogens, antiandrogens, not elsewhere classified, intentional self-harm, sequela	ICD10CM
**T38.7X2**	Poisoning by androgens and anabolic congeners, intentional self-harm	ICD10CM
**T38.7X2A**	Poisoning by androgens and anabolic congeners, intentional self-harm, initial encounter	ICD10CM
**T38.7X2D**	Poisoning by androgens and anabolic congeners, intentional self-harm, subsequent encounter	ICD10CM
**T38.7X2S**	Poisoning by androgens and anabolic congeners, intentional self-harm, sequela	ICD10CM
**T38.802**	Poisoning by unspecified hormones and synthetic substitutes, intentional self-harm	ICD10CM
**T38.802A**	Poisoning by unspecified hormones and synthetic substitutes, intentional self-harm, initial encounter	ICD10CM
**T38.802D**	Poisoning by unspecified hormones and synthetic substitutes, intentional self-harm, subsequent encounter	ICD10CM
**T38.802S**	Poisoning by unspecified hormones and synthetic substitutes, intentional self-harm, sequela	ICD10CM

© 2021 Walsh CG et al. *JAMA Network Open.*

T38.812	Poisoning by anterior pituitary [adenohypophyseal] hormones, intentional self-harm	ICD10CM
T38.812A	Poisoning by anterior pituitary [adenohypophyseal] hormones, intentional self-harm, initial encounter	ICD10CM
T38.812D	Poisoning by anterior pituitary [adenohypophyseal] hormones, intentional self-harm, subsequent encounter	ICD10CM
T38.812S	Poisoning by anterior pituitary [adenohypophyseal] hormones, intentional self-harm, sequela	ICD10CM
T38.892	Poisoning by other hormones and synthetic substitutes, intentional self-harm	ICD10CM
T38.892A	Poisoning by other hormones and synthetic substitutes, intentional self-harm, initial encounter	ICD10CM
T38.892D	Poisoning by other hormones and synthetic substitutes, intentional self-harm, subsequent encounter	ICD10CM
T38.892S	Poisoning by other hormones and synthetic substitutes, intentional self-harm, sequela	ICD10CM
T38.902	Poisoning by unspecified hormone antagonists, intentional self-harm	ICD10CM
T38.902A	Poisoning by unspecified hormone antagonists, intentional self-harm, initial encounter	ICD10CM
T38.902D	Poisoning by unspecified hormone antagonists, intentional self-harm, subsequent encounter	ICD10CM
T38.902S	Poisoning by unspecified hormone antagonists, intentional self-harm, sequela	ICD10CM
T38.992	Poisoning by other hormone antagonists, intentional self-harm	ICD10CM
T38.992A	Poisoning by other hormone antagonists, intentional self-harm, initial encounter	ICD10CM
T38.992D	Poisoning by other hormone antagonists, intentional self-harm, subsequent encounter	ICD10CM
T38.992S	Poisoning by other hormone antagonists, intentional self-harm, sequela	ICD10CM
T39.012	Poisoning by aspirin, intentional self-harm	ICD10CM
T39.012A	Poisoning by aspirin, intentional self-harm, initial encounter	ICD10CM
T39.012D	Poisoning by aspirin, intentional self-harm, subsequent encounter	ICD10CM

© 2021 Walsh CG et al. *JAMA Network Open.*

T39.012S	Poisoning by aspirin, intentional self-harm, sequela	ICD10CM
T39.092	Poisoning by salicylates, intentional self-harm	ICD10CM
T39.092A	Poisoning by salicylates, intentional self-harm, initial encounter	ICD10CM
T39.092D	Poisoning by salicylates, intentional self-harm, subsequent encounter	ICD10CM
T39.092S	Poisoning by salicylates, intentional self-harm, sequela	ICD10CM
T39.1X2	Poisoning by 4-Aminophenol derivatives, intentional self-harm	ICD10CM
T39.1X2A	Poisoning by 4-Aminophenol derivatives, intentional self-harm, initial encounter	ICD10CM
T39.1X2D	Poisoning by 4-Aminophenol derivatives, intentional self-harm, subsequent encounter	ICD10CM
T39.1X2S	Poisoning by 4-Aminophenol derivatives, intentional self-harm, sequela	ICD10CM
T39.2X2	Poisoning by pyrazolone derivatives, intentional self-harm	ICD10CM
T39.2X2A	Poisoning by pyrazolone derivatives, intentional self-harm, initial encounter	ICD10CM
T39.2X2D	Poisoning by pyrazolone derivatives, intentional self-harm, subsequent encounter	ICD10CM
T39.2X2S	Poisoning by pyrazolone derivatives, intentional self-harm, sequela	ICD10CM
T39.312	Poisoning by propionic acid derivatives, intentional self-harm	ICD10CM
T39.312A	Poisoning by propionic acid derivatives, intentional self-harm, initial encounter	ICD10CM
T39.312D	Poisoning by propionic acid derivatives, intentional self-harm, subsequent encounter	ICD10CM
T39.312S	Poisoning by propionic acid derivatives, intentional self-harm, sequela	ICD10CM
T39.392	Poisoning by other nonsteroidal anti-inflammatory drugs [NSAID], intentional self-harm	ICD10CM
T39.392A	Poisoning by other nonsteroidal anti-inflammatory drugs [NSAID], intentional self-harm, initial encounter	ICD10CM
T39.392D	Poisoning by other nonsteroidal anti-inflammatory drugs [NSAID], intentional self-harm, subsequent encounter	ICD10CM

© 2021 Walsh CG et al. *JAMA Network Open.*

T39.392S	Poisoning by other nonsteroidal anti-inflammatory drugs [NSAID], intentional self-harm, sequela	ICD10CM
T39.4X2	Poisoning by antirheumatics, not elsewhere classified, intentional self-harm	ICD10CM
T39.4X2A	Poisoning by antirheumatics, not elsewhere classified, intentional self-harm, initial encounter	ICD10CM
T39.4X2D	Poisoning by antirheumatics, not elsewhere classified, intentional self-harm, subsequent encounter	ICD10CM
T39.4X2S	Poisoning by antirheumatics, not elsewhere classified, intentional self-harm, sequela	ICD10CM
T39.8X2	Poisoning by other nonopioid analgesics and antipyretics, not elsewhere classified, intentional self-harm	ICD10CM
T39.8X2A	Poisoning by other nonopioid analgesics and antipyretics, not elsewhere classified, intentional self-harm, initial encounter	ICD10CM
T39.8X2D	Poisoning by other nonopioid analgesics and antipyretics, not elsewhere classified, intentional self-harm, subsequent encounter	ICD10CM
T39.8X2S	Poisoning by other nonopioid analgesics and antipyretics, not elsewhere classified, intentional self-harm, sequela	ICD10CM
T39.92	Poisoning by unspecified nonopioid analgesic, antipyretic and antirheumatic, intentional self-harm	ICD10CM
T39.92XA	Poisoning by unspecified nonopioid analgesic, antipyretic and antirheumatic, intentional self-harm, initial encounter	ICD10CM
T39.92XD	Poisoning by unspecified nonopioid analgesic, antipyretic and antirheumatic, intentional self-harm, subsequent encounter	ICD10CM
T39.92XS	Poisoning by unspecified nonopioid analgesic, antipyretic and antirheumatic, intentional self-harm, sequela	ICD10CM
T40.0X2	Poisoning by opium, intentional self-harm	ICD10CM
T40.0X2A	Poisoning by opium, intentional self-harm, initial encounter	ICD10CM
T40.0X2D	Poisoning by opium, intentional self-harm, subsequent encounter	ICD10CM
T40.0X2S	Poisoning by opium, intentional self-harm, sequela	ICD10CM
T40.1X2	Poisoning by heroin, intentional self-harm	ICD10CM
T40.1X2A	Poisoning by heroin, intentional self-harm, initial encounter	ICD10CM

© 2021 Walsh CG et al. *JAMA Network Open.*

T40.1X2D	Poisoning by heroin, intentional self-harm, subsequent encounter	ICD10CM
T40.1X2S	Poisoning by heroin, intentional self-harm, sequela	ICD10CM
T40.2X2	Poisoning by other opioids, intentional self-harm	ICD10CM
T40.2X2A	Poisoning by other opioids, intentional self-harm, initial encounter	ICD10CM
T40.2X2D	Poisoning by other opioids, intentional self-harm, subsequent encounter	ICD10CM
T40.2X2S	Poisoning by other opioids, intentional self-harm, sequela	ICD10CM
T40.3X2	Poisoning by methadone, intentional self-harm	ICD10CM
T40.3X2A	Poisoning by methadone, intentional self-harm, initial encounter	ICD10CM
T40.3X2D	Poisoning by methadone, intentional self-harm, subsequent encounter	ICD10CM
T40.3X2S	Poisoning by methadone, intentional self-harm, sequela	ICD10CM
T40.4X2	Poisoning by other synthetic narcotics, intentional self-harm	ICD10CM
T40.4X2A	Poisoning by other synthetic narcotics, intentional self-harm, initial encounter	ICD10CM
T40.4X2D	Poisoning by other synthetic narcotics, intentional self-harm, subsequent encounter	ICD10CM
T40.4X2S	Poisoning by other synthetic narcotics, intentional self-harm, sequela	ICD10CM
T40.5X2	Poisoning by cocaine, intentional self-harm	ICD10CM
T40.5X2A	Poisoning by cocaine, intentional self-harm, initial encounter	ICD10CM
T40.5X2D	Poisoning by cocaine, intentional self-harm, subsequent encounter	ICD10CM
T40.5X2S	Poisoning by cocaine, intentional self-harm, sequela	ICD10CM
T40.602	Poisoning by unspecified narcotics, intentional self-harm	ICD10CM
T40.602A	Poisoning by unspecified narcotics, intentional self-harm, initial encounter	ICD10CM
T40.602D	Poisoning by unspecified narcotics, intentional self-harm, subsequent encounter	ICD10CM
T40.602S	Poisoning by unspecified narcotics, intentional self-harm, sequela	ICD10CM

© 2021 Walsh CG et al. *JAMA Network Open.*

**T40.692**	Poisoning by other narcotics, intentional self-harm	ICD10CM
**T40.692A**	Poisoning by other narcotics, intentional self-harm, initial encounter	ICD10CM
**T40.692D**	Poisoning by other narcotics, intentional self-harm, subsequent encounter	ICD10CM
**T40.692S**	Poisoning by other narcotics, intentional self-harm, sequela	ICD10CM
**T40.7X2**	Poisoning by cannabis (derivatives), intentional self-harm	ICD10CM
**T40.7X2A**	Poisoning by cannabis (derivatives), intentional self-harm, initial encounter	ICD10CM
**T40.7X2D**	Poisoning by cannabis (derivatives), intentional self-harm, subsequent encounter	ICD10CM
**T40.7X2S**	Poisoning by cannabis (derivatives), intentional self-harm, sequela	ICD10CM
**T40.8X2**	Poisoning by lysergide [LSD], intentional self-harm	ICD10CM
**T40.8X2A**	Poisoning by lysergide [LSD], intentional self-harm, initial encounter	ICD10CM
**T40.8X2D**	Poisoning by lysergide [LSD], intentional self-harm, subsequent encounter	ICD10CM
**T40.8X2S**	Poisoning by lysergide [LSD], intentional self-harm, sequela	ICD10CM
**T40.902**	Poisoning by unspecified psychodysleptics [hallucinogens], intentional self-harm	ICD10CM
**T40.902A**	Poisoning by unspecified psychodysleptics [hallucinogens], intentional self-harm, initial encounter	ICD10CM
**T40.902D**	Poisoning by unspecified psychodysleptics [hallucinogens], intentional self-harm, subsequent encounter	ICD10CM
**T40.902S**	Poisoning by unspecified psychodysleptics [hallucinogens], intentional self-harm, sequela	ICD10CM
**T40.992**	Poisoning by other psychodysleptics [hallucinogens], intentional self-harm	ICD10CM
**T40.992A**	Poisoning by other psychodysleptics [hallucinogens], intentional self-harm, initial encounter	ICD10CM
**T40.992D**	Poisoning by other psychodysleptics [hallucinogens], intentional self-harm, subsequent encounter	ICD10CM
**T40.992S**	Poisoning by other psychodysleptics [hallucinogens], intentional self-harm, sequela	ICD10CM
**T41.0X2**	Poisoning by inhaled anesthetics, intentional self-harm	ICD10CM

© 2021 Walsh CG et al. *JAMA Network Open.*

T41.0X2A	Poisoning by inhaled anesthetics, intentional self-harm, initial encounter	ICD10CM
T41.0X2D	Poisoning by inhaled anesthetics, intentional self-harm, subsequent encounter	ICD10CM
T41.0X2S	Poisoning by inhaled anesthetics, intentional self-harm, sequela	ICD10CM
T41.1X2	Poisoning by intravenous anesthetics, intentional self-harm	ICD10CM
T41.1X2A	Poisoning by intravenous anesthetics, intentional self-harm, initial encounter	ICD10CM
T41.1X2D	Poisoning by intravenous anesthetics, intentional self-harm, subsequent encounter	ICD10CM
T41.1X2S	Poisoning by intravenous anesthetics, intentional self-harm, sequela	ICD10CM
T41.202	Poisoning by unspecified general anesthetics, intentional self-harm	ICD10CM
T41.202A	Poisoning by unspecified general anesthetics, intentional self-harm, initial encounter	ICD10CM
T41.202D	Poisoning by unspecified general anesthetics, intentional self-harm, subsequent encounter	ICD10CM
T41.202S	Poisoning by unspecified general anesthetics, intentional self-harm, sequela	ICD10CM
T41.292	Poisoning by other general anesthetics, intentional self-harm	ICD10CM
T41.292A	Poisoning by other general anesthetics, intentional self-harm, initial encounter	ICD10CM
T41.292D	Poisoning by other general anesthetics, intentional self-harm, subsequent encounter	ICD10CM
T41.292S	Poisoning by other general anesthetics, intentional self-harm, sequela	ICD10CM
T41.3X2	Poisoning by local anesthetics, intentional self-harm	ICD10CM
T41.3X2A	Poisoning by local anesthetics, intentional self-harm, initial encounter	ICD10CM
T41.3X2D	Poisoning by local anesthetics, intentional self-harm, subsequent encounter	ICD10CM
T41.3X2S	Poisoning by local anesthetics, intentional self-harm, sequela	ICD10CM
T41.42	Poisoning by unspecified anesthetic, intentional self-harm	ICD10CM
T41.42XA	Poisoning by unspecified anesthetic, intentional self-harm, initial encounter	ICD10CM
T41.42XD	Poisoning by unspecified anesthetic, intentional self-harm, subsequent encounter	ICD10CM

© 2021 Walsh CG et al. *JAMA Network Open.*

T41.42XS	Poisoning by unspecified anesthetic, intentional self-harm, sequela	ICD10CM
T41.5X2	Poisoning by therapeutic gases, intentional self-harm	ICD10CM
T41.5X2A	Poisoning by therapeutic gases, intentional self-harm, initial encounter	ICD10CM
T41.5X2D	Poisoning by therapeutic gases, intentional self-harm, subsequent encounter	ICD10CM
T41.5X2S	Poisoning by therapeutic gases, intentional self-harm, sequela	ICD10CM
T42.0X2	Poisoning by hydantoin derivatives, intentional self-harm	ICD10CM
T42.0X2A	Poisoning by hydantoin derivatives, intentional self-harm, initial encounter	ICD10CM
T42.0X2D	Poisoning by hydantoin derivatives, intentional self-harm, subsequent encounter	ICD10CM
T42.0X2S	Poisoning by hydantoin derivatives, intentional self-harm, sequela	ICD10CM
T42.1X2	Poisoning by iminostilbenes, intentional self-harm	ICD10CM
T42.1X2A	Poisoning by iminostilbenes, intentional self-harm, initial encounter	ICD10CM
T42.1X2D	Poisoning by iminostilbenes, intentional self-harm, subsequent encounter	ICD10CM
T42.1X2S	Poisoning by iminostilbenes, intentional self-harm, sequela	ICD10CM
T42.2X2	Poisoning by succinimides and oxazolidinediones, intentional self-harm	ICD10CM
T42.2X2A	Poisoning by succinimides and oxazolidinediones, intentional self-harm, initial encounter	ICD10CM
T42.2X2D	Poisoning by succinimides and oxazolidinediones, intentional self-harm, subsequent encounter	ICD10CM
T42.2X2S	Poisoning by succinimides and oxazolidinediones, intentional self-harm, sequela	ICD10CM
T42.3X2	Poisoning by barbiturates, intentional self-harm	ICD10CM
T42.3X2A	Poisoning by barbiturates, intentional self-harm, initial encounter	ICD10CM
T42.3X2D	Poisoning by barbiturates, intentional self-harm, subsequent encounter	ICD10CM
T42.3X2S	Poisoning by barbiturates, intentional self-harm, sequela	ICD10CM
T42.4X2	Poisoning by benzodiazepines, intentional self-harm	ICD10CM

© 2021 Walsh CG et al. *JAMA Network Open.*

T42.4X2A	Poisoning by benzodiazepines, intentional self-harm, initial encounter	ICD10CM
T42.4X2D	Poisoning by benzodiazepines, intentional self-harm, subsequent encounter	ICD10CM
T42.4X2S	Poisoning by benzodiazepines, intentional self-harm, sequela	ICD10CM
T42.5X2	Poisoning by mixed antiepileptics, intentional self-harm	ICD10CM
T42.5X2A	Poisoning by mixed antiepileptics, intentional self-harm, initial encounter	ICD10CM
T42.5X2D	Poisoning by mixed antiepileptics, intentional self-harm, subsequent encounter	ICD10CM
T42.5X2S	Poisoning by mixed antiepileptics, intentional self-harm, sequela	ICD10CM
T42.6X2	Poisoning by other antiepileptic and sedative-hypnotic drugs, intentional self-harm	ICD10CM
T42.6X2A	Poisoning by other antiepileptic and sedative-hypnotic drugs, intentional self-harm, initial encounter	ICD10CM
T42.6X2D	Poisoning by other antiepileptic and sedative-hypnotic drugs, intentional self-harm, subsequent encounter	ICD10CM
T42.6X2S	Poisoning by other antiepileptic and sedative-hypnotic drugs, intentional self-harm, sequela	ICD10CM
T42.72	Poisoning by unspecified antiepileptic and sedative-hypnotic drugs, intentional self-harm	ICD10CM
T42.72XA	Poisoning by unspecified antiepileptic and sedative-hypnotic drugs, intentional self-harm, initial encounter	ICD10CM
T42.72XD	Poisoning by unspecified antiepileptic and sedative-hypnotic drugs, intentional self-harm, subsequent encounter	ICD10CM
T42.72XS	Poisoning by unspecified antiepileptic and sedative-hypnotic drugs, intentional self-harm, sequela	ICD10CM
T42.8X2	Poisoning by antiparkinsonism drugs and other central muscle-tone depressants, intentional self-harm	ICD10CM
T42.8X2A	Poisoning by antiparkinsonism drugs and other central muscle-tone depressants, intentional self-harm, initial encounter	ICD10CM
T42.8X2D	Poisoning by antiparkinsonism drugs and other central muscle-tone depressants, intentional self-harm, subsequent encounter	ICD10CM
T42.8X2S	Poisoning by antiparkinsonism drugs and other central muscle-tone depressants, intentional self-harm, sequela	ICD10CM

© 2021 Walsh CG et al. *JAMA Network Open.*

T43.012	Poisoning by tricyclic antidepressants, intentional self-harm	ICD10CM
T43.012A	Poisoning by tricyclic antidepressants, intentional self-harm, initial encounter	ICD10CM
T43.012D	Poisoning by tricyclic antidepressants, intentional self-harm, subsequent encounter	ICD10CM
T43.012S	Poisoning by tricyclic antidepressants, intentional self-harm, sequela	ICD10CM
T43.022	Poisoning by tetracyclic antidepressants, intentional self-harm	ICD10CM
T43.022A	Poisoning by tetracyclic antidepressants, intentional self-harm, initial encounter	ICD10CM
T43.022D	Poisoning by tetracyclic antidepressants, intentional self-harm, subsequent encounter	ICD10CM
T43.022S	Poisoning by tetracyclic antidepressants, intentional self-harm, sequela	ICD10CM
T43.0x2A	Poisoning by tricyclic and tetracyclic antidepressants, intentional self-harm, initial encounter	ICD10CM
T43.0x2D	Poisoning by tricyclic and tetracyclic antidepressants, intentional self-harm, subsequent encounter	ICD10CM
T43.0x2S	Poisoning by tricyclic and tetracyclic antidepressants, intentional self-harm, sequela	ICD10CM
T43.1X2	Poisoning by monoamine-oxidase-inhibitor antidepressants, intentional self-harm	ICD10CM
T43.1X2A	Poisoning by monoamine-oxidase-inhibitor antidepressants, intentional self-harm, initial encounter	ICD10CM
T43.1X2D	Poisoning by monoamine-oxidase-inhibitor antidepressants, intentional self-harm, subsequent encounter	ICD10CM
T43.1X2S	Poisoning by monoamine-oxidase-inhibitor antidepressants, intentional self-harm, sequela	ICD10CM
T43.202	Poisoning by unspecified antidepressants, intentional self-harm	ICD10CM
T43.202A	Poisoning by unspecified antidepressants, intentional self-harm, initial encounter	ICD10CM
T43.202D	Poisoning by unspecified antidepressants, intentional self-harm, subsequent encounter	ICD10CM
T43.202S	Poisoning by unspecified antidepressants, intentional self-harm, sequela	ICD10CM
T43.212	Poisoning by selective serotonin and norepinephrine reuptake inhibitors, intentional self-harm	ICD10CM

© 2021 Walsh CG et al. *JAMA Network Open.*

T43.212A	Poisoning by selective serotonin and norepinephrine reuptake inhibitors, intentional self-harm, initial encounter	ICD10CM
T43.212D	Poisoning by selective serotonin and norepinephrine reuptake inhibitors, intentional self-harm, subsequent encounter	ICD10CM
T43.212S	Poisoning by selective serotonin and norepinephrine reuptake inhibitors, intentional self-harm, sequela	ICD10CM
T43.222	Poisoning by selective serotonin reuptake inhibitors, intentional self-harm	ICD10CM
T43.222A	Poisoning by selective serotonin reuptake inhibitors, intentional self-harm, initial encounter	ICD10CM
T43.222D	Poisoning by selective serotonin reuptake inhibitors, intentional self-harm, subsequent encounter	ICD10CM
T43.222S	Poisoning by selective serotonin reuptake inhibitors, intentional self-harm, sequela	ICD10CM
T43.292	Poisoning by other antidepressants, intentional self-harm	ICD10CM
T43.292A	Poisoning by other antidepressants, intentional self-harm, initial encounter	ICD10CM
T43.292D	Poisoning by other antidepressants, intentional self-harm, subsequent encounter	ICD10CM
T43.292S	Poisoning by other antidepressants, intentional self-harm, sequela	ICD10CM
T43.3X2	Poisoning by phenothiazine antipsychotics and neuroleptics, intentional self-harm	ICD10CM
T43.3X2A	Poisoning by phenothiazine antipsychotics and neuroleptics, intentional self-harm, initial encounter	ICD10CM
T43.3X2D	Poisoning by phenothiazine antipsychotics and neuroleptics, intentional self-harm, subsequent encounter	ICD10CM
T43.3X2S	Poisoning by phenothiazine antipsychotics and neuroleptics, intentional self-harm, sequela	ICD10CM
T43.4X2	Poisoning by butyrophenone and thiothixene neuroleptics, intentional self-harm	ICD10CM
T43.4X2A	Poisoning by butyrophenone and thiothixene neuroleptics, intentional self-harm, initial encounter	ICD10CM
T43.4X2D	Poisoning by butyrophenone and thiothixene neuroleptics, intentional self-harm, subsequent encounter	ICD10CM
T43.4X2S	Poisoning by butyrophenone and thiothixene neuroleptics, intentional self-harm, sequela	ICD10CM

© 2021 Walsh CG et al. *JAMA Network Open.*

T43.502	Poisoning by unspecified antipsychotics and neuroleptics, intentional self-harm	ICD10CM
T43.502A	Poisoning by unspecified antipsychotics and neuroleptics, intentional self-harm, initial encounter	ICD10CM
T43.502D	Poisoning by unspecified antipsychotics and neuroleptics, intentional self-harm, subsequent encounter	ICD10CM
T43.502S	Poisoning by unspecified antipsychotics and neuroleptics, intentional self-harm, sequela	ICD10CM
T43.592	Poisoning by other antipsychotics and neuroleptics, intentional self-harm	ICD10CM
T43.592A	Poisoning by other antipsychotics and neuroleptics, intentional self-harm, initial encounter	ICD10CM
T43.592D	Poisoning by other antipsychotics and neuroleptics, intentional self-harm, subsequent encounter	ICD10CM
T43.592S	Poisoning by other antipsychotics and neuroleptics, intentional self-harm, sequela	ICD10CM
T43.602	Poisoning by unspecified psychostimulants, intentional self-harm	ICD10CM
T43.602A	Poisoning by unspecified psychostimulants, intentional self-harm, initial encounter	ICD10CM
T43.602D	Poisoning by unspecified psychostimulants, intentional self-harm, subsequent encounter	ICD10CM
T43.602S	Poisoning by unspecified psychostimulants, intentional self-harm, sequela	ICD10CM
T43.612	Poisoning by caffeine, intentional self-harm	ICD10CM
T43.612A	Poisoning by caffeine, intentional self-harm, initial encounter	ICD10CM
T43.612D	Poisoning by caffeine, intentional self-harm, subsequent encounter	ICD10CM
T43.612S	Poisoning by caffeine, intentional self-harm, sequela	ICD10CM
T43.622	Poisoning by amphetamines, intentional self-harm	ICD10CM
T43.622A	Poisoning by amphetamines, intentional self-harm, initial encounter	ICD10CM
T43.622D	Poisoning by amphetamines, intentional self-harm, subsequent encounter	ICD10CM
T43.622S	Poisoning by amphetamines, intentional self-harm, sequela	ICD10CM

© 2021 Walsh CG et al. *JAMA Network Open.*

T43.632	Poisoning by methylphenidate, intentional self-harm	ICD10CM
T43.632A	Poisoning by methylphenidate, intentional self-harm, initial encounter	ICD10CM
T43.632D	Poisoning by methylphenidate, intentional self-harm, subsequent encounter	ICD10CM
T43.632S	Poisoning by methylphenidate, intentional self-harm, sequela	ICD10CM
T43.642	Poisoning by ecstasy, intentional self-harm	ICD10CM
T43.642A	Poisoning by ecstasy, intentional self-harm, initial encounter	ICD10CM
T43.642D	Poisoning by ecstasy, intentional self-harm, subsequent encounter	ICD10CM
T43.642S	Poisoning by ecstasy, intentional self-harm, sequela	ICD10CM
T43.692	Poisoning by other psychostimulants, intentional self-harm	ICD10CM
T43.692A	Poisoning by other psychostimulants, intentional self-harm, initial encounter	ICD10CM
T43.692D	Poisoning by other psychostimulants, intentional self-harm, subsequent encounter	ICD10CM
T43.692S	Poisoning by other psychostimulants, intentional self-harm, sequela	ICD10CM
T43.6x2A	Poisoning by psychostimulants with abuse potential, intentional self-harm, initial encounter	ICD10CM
T43.6x2D	Poisoning by psychostimulants with abuse potential, intentional self-harm, subsequent encounter	ICD10CM
T43.6x2S	Poisoning by psychostimulants with abuse potential, intentional self-harm, sequela	ICD10CM
T43.8X2	Poisoning by other psychotropic drugs, intentional self-harm	ICD10CM
T43.8X2A	Poisoning by other psychotropic drugs, intentional self-harm, initial encounter	ICD10CM
T43.8X2D	Poisoning by other psychotropic drugs, intentional self-harm, subsequent encounter	ICD10CM
T43.8X2S	Poisoning by other psychotropic drugs, intentional self-harm, sequela	ICD10CM
T43.92	Poisoning by unspecified psychotropic drug, intentional self-harm	ICD10CM
T43.92XA	Poisoning by unspecified psychotropic drug, intentional self-harm, initial encounter	ICD10CM

© 2021 Walsh CG et al. *JAMA Network Open.*

T43.92XD	Poisoning by unspecified psychotropic drug, intentional self-harm, subsequent encounter	ICD10CM
T43.92XS	Poisoning by unspecified psychotropic drug, intentional self-harm, sequela	ICD10CM
T44.0X2	Poisoning by anticholinesterase agents, intentional self-harm	ICD10CM
T44.0X2A	Poisoning by anticholinesterase agents, intentional self-harm, initial encounter	ICD10CM
T44.0X2D	Poisoning by anticholinesterase agents, intentional self-harm, subsequent encounter	ICD10CM
T44.0X2S	Poisoning by anticholinesterase agents, intentional self-harm, sequela	ICD10CM
T44.1X2	Poisoning by other parasympathomimetics [cholinergics], intentional self-harm	ICD10CM
T44.1X2A	Poisoning by other parasympathomimetics [cholinergics], intentional self-harm, initial encounter	ICD10CM
T44.1X2D	Poisoning by other parasympathomimetics [cholinergics], intentional self-harm, subsequent encounter	ICD10CM
T44.1X2S	Poisoning by other parasympathomimetics [cholinergics], intentional self-harm, sequela	ICD10CM
T44.2X2	Poisoning by ganglionic blocking drugs, intentional self-harm	ICD10CM
T44.2X2A	Poisoning by ganglionic blocking drugs, intentional self-harm, initial encounter	ICD10CM
T44.2X2D	Poisoning by ganglionic blocking drugs, intentional self-harm, subsequent encounter	ICD10CM
T44.2X2S	Poisoning by ganglionic blocking drugs, intentional self-harm, sequela	ICD10CM
T44.3X2	Poisoning by other parasympatholytics [anticholinergics and antimuscarinics] and spasmolytics, intentional self-harm	ICD10CM
T44.3X2A	Poisoning by other parasympatholytics [anticholinergics and antimuscarinics] and spasmolytics, intentional self-harm, initial encounter	ICD10CM
T44.3X2D	Poisoning by other parasympatholytics [anticholinergics and antimuscarinics] and spasmolytics, intentional self-harm, subsequent encounter	ICD10CM
T44.3X2S	Poisoning by other parasympatholytics [anticholinergics and antimuscarinics] and spasmolytics, intentional self-harm, sequela	ICD10CM
T44.4X2	Poisoning by predominantly alpha-adrenoreceptor agonists, intentional self-harm	ICD10CM

© 2021 Walsh CG et al. *JAMA Network Open.*

T44.4X2A	Poisoning by predominantly alpha-adrenoreceptor agonists, intentional self-harm, initial encounter	ICD10CM
T44.4X2D	Poisoning by predominantly alpha-adrenoreceptor agonists, intentional self-harm, subsequent encounter	ICD10CM
T44.4X2S	Poisoning by predominantly alpha-adrenoreceptor agonists, intentional self-harm, sequela	ICD10CM
T44.5X2	Poisoning by predominantly beta-adrenoreceptor agonists, intentional self-harm	ICD10CM
T44.5X2A	Poisoning by predominantly beta-adrenoreceptor agonists, intentional self-harm, initial encounter	ICD10CM
T44.5X2D	Poisoning by predominantly beta-adrenoreceptor agonists, intentional self-harm, subsequent encounter	ICD10CM
T44.5X2S	Poisoning by predominantly beta-adrenoreceptor agonists, intentional self-harm, sequela	ICD10CM
T44.6X2	Poisoning by alpha-adrenoreceptor antagonists, intentional self-harm	ICD10CM
T44.6X2A	Poisoning by alpha-adrenoreceptor antagonists, intentional self-harm, initial encounter	ICD10CM
T44.6X2D	Poisoning by alpha-adrenoreceptor antagonists, intentional self-harm, subsequent encounter	ICD10CM
T44.6X2S	Poisoning by alpha-adrenoreceptor antagonists, intentional self-harm, sequela	ICD10CM
T44.7X2	Poisoning by beta-adrenoreceptor antagonists, intentional self-harm	ICD10CM
T44.7X2A	Poisoning by beta-adrenoreceptor antagonists, intentional self-harm, initial encounter	ICD10CM
T44.7X2D	Poisoning by beta-adrenoreceptor antagonists, intentional self-harm, subsequent encounter	ICD10CM
T44.7X2S	Poisoning by beta-adrenoreceptor antagonists, intentional self-harm, sequela	ICD10CM
T44.8X2	Poisoning by centrally-acting and adrenergic-neuron-blocking agents, intentional self-harm	ICD10CM
T44.8X2A	Poisoning by centrally-acting and adrenergic-neuron-blocking agents, intentional self-harm, initial encounter	ICD10CM
T44.8X2D	Poisoning by centrally-acting and adrenergic-neuron-blocking agents, intentional self-harm, subsequent encounter	ICD10CM

© 2021 Walsh CG et al. *JAMA Network Open.*

**T44.8X2S**	Poisoning by centrally-acting and adrenergic-neuron-blocking agents, intentional self-harm, sequela	ICD10CM
**T44.902**	Poisoning by unspecified drugs primarily affecting the autonomic nervous system, intentional self-harm	ICD10CM
**T44.902A**	Poisoning by unspecified drugs primarily affecting the autonomic nervous system, intentional self-harm, initial encounter	ICD10CM
**T44.902D**	Poisoning by unspecified drugs primarily affecting the autonomic nervous system, intentional self-harm, subsequent encounter	ICD10CM
**T44.902S**	Poisoning by unspecified drugs primarily affecting the autonomic nervous system, intentional self-harm, sequela	ICD10CM
**T44.992**	Poisoning by other drug primarily affecting the autonomic nervous system, intentional self-harm	ICD10CM
**T44.992A**	Poisoning by other drug primarily affecting the autonomic nervous system, intentional self-harm, initial encounter	ICD10CM
**T44.992D**	Poisoning by other drug primarily affecting the autonomic nervous system, intentional self-harm, subsequent encounter	ICD10CM
**T44.992S**	Poisoning by other drug primarily affecting the autonomic nervous system, intentional self-harm, sequela	ICD10CM
**T45.0X2**	Poisoning by antiallergic and antiemetic drugs, intentional self-harm	ICD10CM
**T45.0X2A**	Poisoning by antiallergic and antiemetic drugs, intentional self-harm, initial encounter	ICD10CM
**T45.0X2D**	Poisoning by antiallergic and antiemetic drugs, intentional self-harm, subsequent encounter	ICD10CM
**T45.0X2S**	Poisoning by antiallergic and antiemetic drugs, intentional self-harm, sequela	ICD10CM
**T45.1X2**	Poisoning by antineoplastic and immunosuppressive drugs, intentional self-harm	ICD10CM
**T45.1X2A**	Poisoning by antineoplastic and immunosuppressive drugs, intentional self-harm, initial encounter	ICD10CM
**T45.1X2D**	Poisoning by antineoplastic and immunosuppressive drugs, intentional self-harm, subsequent encounter	ICD10CM
**T45.1X2S**	Poisoning by antineoplastic and immunosuppressive drugs, intentional self-harm, sequela	ICD10CM
**T45.2X2**	Poisoning by vitamins, intentional self-harm	ICD10CM

© 2021 Walsh CG et al. *JAMA Network Open.*

**T45.2X2A**	Poisoning by vitamins, intentional self-harm, initial encounter	ICD10CM
**T45.2X2D**	Poisoning by vitamins, intentional self-harm, subsequent encounter	ICD10CM
**T45.2X2S**	Poisoning by vitamins, intentional self-harm, sequela	ICD10CM
**T45.3X2**	Poisoning by enzymes, intentional self-harm	ICD10CM
**T45.3X2A**	Poisoning by enzymes, intentional self-harm, initial encounter	ICD10CM
**T45.3X2D**	Poisoning by enzymes, intentional self-harm, subsequent encounter	ICD10CM
**T45.3X2S**	Poisoning by enzymes, intentional self-harm, sequela	ICD10CM
**T45.4X2**	Poisoning by iron and its compounds, intentional self-harm	ICD10CM
**T45.4X2A**	Poisoning by iron and its compounds, intentional self-harm, initial encounter	ICD10CM
**T45.4X2D**	Poisoning by iron and its compounds, intentional self-harm, subsequent encounter	ICD10CM
**T45.4X2S**	Poisoning by iron and its compounds, intentional self-harm, sequela	ICD10CM
**T45.512**	Poisoning by anticoagulants, intentional self-harm	ICD10CM
**T45.512A**	Poisoning by anticoagulants, intentional self-harm, initial encounter	ICD10CM
**T45.512D**	Poisoning by anticoagulants, intentional self-harm, subsequent encounter	ICD10CM
**T45.512S**	Poisoning by anticoagulants, intentional self-harm, sequela	ICD10CM
**T45.522**	Poisoning by antithrombotic drugs, intentional self-harm	ICD10CM
**T45.522A**	Poisoning by antithrombotic drugs, intentional self-harm, initial encounter	ICD10CM
**T45.522D**	Poisoning by antithrombotic drugs, intentional self-harm, subsequent encounter	ICD10CM
**T45.522S**	Poisoning by antithrombotic drugs, intentional self-harm, sequela	ICD10CM
**T45.602**	Poisoning by unspecified fibrinolysis-affecting drugs, intentional self-harm	ICD10CM
**T45.602A**	Poisoning by unspecified fibrinolysis-affecting drugs, intentional self-harm, initial encounter	ICD10CM
**T45.602D**	Poisoning by unspecified fibrinolysis-affecting drugs, intentional self-harm, subsequent encounter	ICD10CM

© 2021 Walsh CG et al. *JAMA Network Open.*

**T45.602S**	Poisoning by unspecified fibrinolysis-affecting drugs, intentional self-harm, sequela	ICD10CM
**T45.612**	Poisoning by thrombolytic drug, intentional self-harm	ICD10CM
**T45.612A**	Poisoning by thrombolytic drug, intentional self-harm, initial encounter	ICD10CM
**T45.612D**	Poisoning by thrombolytic drug, intentional self-harm, subsequent encounter	ICD10CM
**T45.612S**	Poisoning by thrombolytic drug, intentional self-harm, sequela	ICD10CM
**T45.622**	Poisoning by hemostatic drug, intentional self-harm	ICD10CM
**T45.622A**	Poisoning by hemostatic drug, intentional self-harm, initial encounter	ICD10CM
**T45.622D**	Poisoning by hemostatic drug, intentional self-harm, subsequent encounter	ICD10CM
**T45.622S**	Poisoning by hemostatic drug, intentional self-harm, sequela	ICD10CM
**T45.692**	Poisoning by other fibrinolysis-affecting drugs, intentional self-harm	ICD10CM
**T45.692A**	Poisoning by other fibrinolysis-affecting drugs, intentional self-harm, initial encounter	ICD10CM
**T45.692D**	Poisoning by other fibrinolysis-affecting drugs, intentional self-harm, subsequent encounter	ICD10CM
**T45.692S**	Poisoning by other fibrinolysis-affecting drugs, intentional self-harm, sequela	ICD10CM
**T45.7X2**	Poisoning by anticoagulant antagonists, vitamin K and other coagulants, intentional self-harm	ICD10CM
**T45.7X2A**	Poisoning by anticoagulant antagonists, vitamin K and other coagulants, intentional self-harm, initial encounter	ICD10CM
**T45.7X2D**	Poisoning by anticoagulant antagonists, vitamin K and other coagulants, intentional self-harm, subsequent encounter	ICD10CM
**T45.7X2S**	Poisoning by anticoagulant antagonists, vitamin K and other coagulants, intentional self-harm, sequela	ICD10CM
**T45.8X2**	Poisoning by other primarily systemic and hematological agents, intentional self-harm	ICD10CM
**T45.8X2A**	Poisoning by other primarily systemic and hematological agents, intentional self-harm, initial encounter	ICD10CM
**T45.8X2D**	Poisoning by other primarily systemic and hematological agents, intentional self-harm, subsequent encounter	ICD10CM

© 2021 Walsh CG et al. *JAMA Network Open.*

T45.8X2S	Poisoning by other primarily systemic and hematological agents, intentional self-harm, sequela	ICD10CM
T45.92	Poisoning by unspecified primarily systemic and hematological agent, intentional self-harm	ICD10CM
T45.92XA	Poisoning by unspecified primarily systemic and hematological agent, intentional self-harm, initial encounter	ICD10CM
T45.92XD	Poisoning by unspecified primarily systemic and hematological agent, intentional self-harm, subsequent encounter	ICD10CM
T45.92XS	Poisoning by unspecified primarily systemic and hematological agent, intentional self-harm, sequela	ICD10CM
T46.0X2	Poisoning by cardiac-stimulant glycosides and drugs of similar action, intentional self-harm	ICD10CM
T46.0X2A	Poisoning by cardiac-stimulant glycosides and drugs of similar action, intentional self-harm, initial encounter	ICD10CM
T46.0X2D	Poisoning by cardiac-stimulant glycosides and drugs of similar action, intentional self-harm, subsequent encounter	ICD10CM
T46.0X2S	Poisoning by cardiac-stimulant glycosides and drugs of similar action, intentional self-harm, sequela	ICD10CM
T46.1X2	Poisoning by calcium-channel blockers, intentional self-harm	ICD10CM
T46.1X2A	Poisoning by calcium-channel blockers, intentional self-harm, initial encounter	ICD10CM
T46.1X2D	Poisoning by calcium-channel blockers, intentional self-harm, subsequent encounter	ICD10CM
T46.1X2S	Poisoning by calcium-channel blockers, intentional self-harm, sequela	ICD10CM
T46.2X2	Poisoning by other antidysrhythmic drugs, intentional self-harm	ICD10CM
T46.2X2A	Poisoning by other antidysrhythmic drugs, intentional self-harm, initial encounter	ICD10CM
T46.2X2D	Poisoning by other antidysrhythmic drugs, intentional self-harm, subsequent encounter	ICD10CM
T46.2X2S	Poisoning by other antidysrhythmic drugs, intentional self-harm, sequela	ICD10CM
T46.3X2	Poisoning by coronary vasodilators, intentional self-harm	ICD10CM
T46.3X2A	Poisoning by coronary vasodilators, intentional self-harm, initial encounter	ICD10CM

© 2021 Walsh CG et al. *JAMA Network Open.*

**T46.3X2D**	Poisoning by coronary vasodilators, intentional self-harm, subsequent encounter	ICD10CM
**T46.3X2S**	Poisoning by coronary vasodilators, intentional self-harm, sequela	ICD10CM
**T46.4X2**	Poisoning by angiotensin-converting-enzyme inhibitors, intentional self-harm	ICD10CM
**T46.4X2A**	Poisoning by angiotensin-converting-enzyme inhibitors, intentional self-harm, initial encounter	ICD10CM
**T46.4X2D**	Poisoning by angiotensin-converting-enzyme inhibitors, intentional self-harm, subsequent encounter	ICD10CM
**T46.4X2S**	Poisoning by angiotensin-converting-enzyme inhibitors, intentional self-harm, sequela	ICD10CM
**T46.5X2**	Poisoning by other antihypertensive drugs, intentional self-harm	ICD10CM
**T46.5X2A**	Poisoning by other antihypertensive drugs, intentional self-harm, initial encounter	ICD10CM
**T46.5X2D**	Poisoning by other antihypertensive drugs, intentional self-harm, subsequent encounter	ICD10CM
**T46.5X2S**	Poisoning by other antihypertensive drugs, intentional self-harm, sequela	ICD10CM
**T46.6X2**	Poisoning by antihyperlipidemic and antiarteriosclerotic drugs, intentional self-harm	ICD10CM
**T46.6X2A**	Poisoning by antihyperlipidemic and antiarteriosclerotic drugs, intentional self-harm, initial encounter	ICD10CM
**T46.6X2D**	Poisoning by antihyperlipidemic and antiarteriosclerotic drugs, intentional self-harm, subsequent encounter	ICD10CM
**T46.6X2S**	Poisoning by antihyperlipidemic and antiarteriosclerotic drugs, intentional self-harm, sequela	ICD10CM
**T46.7X2**	Poisoning by peripheral vasodilators, intentional self-harm	ICD10CM
**T46.7X2A**	Poisoning by peripheral vasodilators, intentional self-harm, initial encounter	ICD10CM
**T46.7X2D**	Poisoning by peripheral vasodilators, intentional self-harm, subsequent encounter	ICD10CM
**T46.7X2S**	Poisoning by peripheral vasodilators, intentional self-harm, sequela	ICD10CM
**T46.8X2**	Poisoning by antivaricose drugs, including sclerosing agents, intentional self-harm	ICD10CM
**T46.8X2A**	Poisoning by antivaricose drugs, including sclerosing agents, intentional self-harm, initial encounter	ICD10CM

© 2021 Walsh CG et al. *JAMA Network Open.*

**T46.8X2D**	Poisoning by antivaricose drugs, including sclerosing agents, intentional self-harm, subsequent encounter	ICD10CM
**T46.8X2S**	Poisoning by antivaricose drugs, including sclerosing agents, intentional self-harm, sequela	ICD10CM
**T46.902**	Poisoning by unspecified agents primarily affecting the cardiovascular system, intentional self-harm	ICD10CM
**T46.902A**	Poisoning by unspecified agents primarily affecting the cardiovascular system, intentional self-harm, initial encounter	ICD10CM
**T46.902D**	Poisoning by unspecified agents primarily affecting the cardiovascular system, intentional self-harm, subsequent encounter	ICD10CM
**T46.902S**	Poisoning by unspecified agents primarily affecting the cardiovascular system, intentional self-harm, sequela	ICD10CM
**T46.992**	Poisoning by other agents primarily affecting the cardiovascular system, intentional self-harm	ICD10CM
**T46.992A**	Poisoning by other agents primarily affecting the cardiovascular system, intentional self-harm, initial encounter	ICD10CM
**T46.992D**	Poisoning by other agents primarily affecting the cardiovascular system, intentional self-harm, subsequent encounter	ICD10CM
**T46.992S**	Poisoning by other agents primarily affecting the cardiovascular system, intentional self-harm, sequela	ICD10CM
**T47.0X2**	Poisoning by histamine H2-receptor blockers, intentional self-harm	ICD10CM
**T47.0X2A**	Poisoning by histamine H2-receptor blockers, intentional self-harm, initial encounter	ICD10CM
**T47.0X2D**	Poisoning by histamine H2-receptor blockers, intentional self-harm, subsequent encounter	ICD10CM
**T47.0X2S**	Poisoning by histamine H2-receptor blockers, intentional self-harm, sequela	ICD10CM
**T47.1X2**	Poisoning by other antacids and anti-gastric-secretion drugs, intentional self-harm	ICD10CM
**T47.1X2A**	Poisoning by other antacids and anti-gastric-secretion drugs, intentional self-harm, initial encounter	ICD10CM
**T47.1X2D**	Poisoning by other antacids and anti-gastric-secretion drugs, intentional self-harm, subsequent encounter	ICD10CM
**T47.1X2S**	Poisoning by other antacids and anti-gastric-secretion drugs, intentional self-harm, sequela	ICD10CM

© 2021 Walsh CG et al. *JAMA Network Open.*

T47.2X2	Poisoning by stimulant laxatives, intentional self-harm	ICD10CM
T47.2X2A	Poisoning by stimulant laxatives, intentional self-harm, initial encounter	ICD10CM
T47.2X2D	Poisoning by stimulant laxatives, intentional self-harm, subsequent encounter	ICD10CM
T47.2X2S	Poisoning by stimulant laxatives, intentional self-harm, sequela	ICD10CM
T47.3X2	Poisoning by saline and osmotic laxatives, intentional self-harm	ICD10CM
T47.3X2A	Poisoning by saline and osmotic laxatives, intentional self-harm, initial encounter	ICD10CM
T47.3X2D	Poisoning by saline and osmotic laxatives, intentional self-harm, subsequent encounter	ICD10CM
T47.3X2S	Poisoning by saline and osmotic laxatives, intentional self-harm, sequela	ICD10CM
T47.4X2	Poisoning by other laxatives, intentional self-harm	ICD10CM
T47.4X2A	Poisoning by other laxatives, intentional self-harm, initial encounter	ICD10CM
T47.4X2D	Poisoning by other laxatives, intentional self-harm, subsequent encounter	ICD10CM
T47.4X2S	Poisoning by other laxatives, intentional self-harm, sequela	ICD10CM
T47.5X2	Poisoning by digestants, intentional self-harm	ICD10CM
T47.5X2A	Poisoning by digestants, intentional self-harm, initial encounter	ICD10CM
T47.5X2D	Poisoning by digestants, intentional self-harm, subsequent encounter	ICD10CM
T47.5X2S	Poisoning by digestants, intentional self-harm, sequela	ICD10CM
T47.6X2	Poisoning by antidiarrheal drugs, intentional self-harm	ICD10CM
T47.6X2A	Poisoning by antidiarrheal drugs, intentional self-harm, initial encounter	ICD10CM
T47.6X2D	Poisoning by antidiarrheal drugs, intentional self-harm, subsequent encounter	ICD10CM
T47.6X2S	Poisoning by antidiarrheal drugs, intentional self-harm, sequela	ICD10CM
T47.7X2	Poisoning by emetics, intentional self-harm	ICD10CM
T47.7X2A	Poisoning by emetics, intentional self-harm, initial encounter	ICD10CM

© 2021 Walsh CG et al. *JAMA Network Open.*

**T47.7X2D**	Poisoning by emetics, intentional self-harm, subsequent encounter	ICD10CM
**T47.7X2S**	Poisoning by emetics, intentional self-harm, sequela	ICD10CM
**T47.8X2**	Poisoning by other agents primarily affecting gastrointestinal system, intentional self-harm	ICD10CM
**T47.8X2A**	Poisoning by other agents primarily affecting gastrointestinal system, intentional self-harm, initial encounter	ICD10CM
**T47.8X2D**	Poisoning by other agents primarily affecting gastrointestinal system, intentional self-harm, subsequent encounter	ICD10CM
**T47.8X2S**	Poisoning by other agents primarily affecting gastrointestinal system, intentional self-harm, sequela	ICD10CM
**T47.92**	Poisoning by unspecified agents primarily affecting the gastrointestinal system, intentional self-harm	ICD10CM
**T47.92XA**	Poisoning by unspecified agents primarily affecting the gastrointestinal system, intentional self-harm, initial encounter	ICD10CM
**T47.92XD**	Poisoning by unspecified agents primarily affecting the gastrointestinal system, intentional self-harm, subsequent encounter	ICD10CM
**T47.92XS**	Poisoning by unspecified agents primarily affecting the gastrointestinal system, intentional self-harm, sequela	ICD10CM
**T48.0X2**	Poisoning by oxytocic drugs, intentional self-harm	ICD10CM
**T48.0X2A**	Poisoning by oxytocic drugs, intentional self-harm, initial encounter	ICD10CM
**T48.0X2D**	Poisoning by oxytocic drugs, intentional self-harm, subsequent encounter	ICD10CM
**T48.0X2S**	Poisoning by oxytocic drugs, intentional self-harm, sequela	ICD10CM
**T48.1X2**	Poisoning by skeletal muscle relaxants [neuromuscular blocking agents], intentional self-harm	ICD10CM
**T48.1X2A**	Poisoning by skeletal muscle relaxants [neuromuscular blocking agents], intentional self-harm, initial encounter	ICD10CM
**T48.1X2D**	Poisoning by skeletal muscle relaxants [neuromuscular blocking agents], intentional self-harm, subsequent encounter	ICD10CM
**T48.1X2S**	Poisoning by skeletal muscle relaxants [neuromuscular blocking agents], intentional self-harm, sequela	ICD10CM
**T48.202**	Poisoning by unspecified drugs acting on muscles, intentional self-harm	ICD10CM

© 2021 Walsh CG et al. *JAMA Network Open.*

**T48.202A**	Poisoning by unspecified drugs acting on muscles, intentional self-harm, initial encounter	ICD10CM
**T48.202D**	Poisoning by unspecified drugs acting on muscles, intentional self-harm, subsequent encounter	ICD10CM
**T48.202S**	Poisoning by unspecified drugs acting on muscles, intentional self-harm, sequela	ICD10CM
**T48.292**	Poisoning by other drugs acting on muscles, intentional self-harm	ICD10CM
**T48.292A**	Poisoning by other drugs acting on muscles, intentional self-harm, initial encounter	ICD10CM
**T48.292D**	Poisoning by other drugs acting on muscles, intentional self-harm, subsequent encounter	ICD10CM
**T48.292S**	Poisoning by other drugs acting on muscles, intentional self-harm, sequela	ICD10CM
**T48.3X2**	Poisoning by antitussives, intentional self-harm	ICD10CM
**T48.3X2A**	Poisoning by antitussives, intentional self-harm, initial encounter	ICD10CM
**T48.3X2D**	Poisoning by antitussives, intentional self-harm, subsequent encounter	ICD10CM
**T48.3X2S**	Poisoning by antitussives, intentional self-harm, sequela	ICD10CM
**T48.4X2**	Poisoning by expectorants, intentional self-harm	ICD10CM
**T48.4X2A**	Poisoning by expectorants, intentional self-harm, initial encounter	ICD10CM
**T48.4X2D**	Poisoning by expectorants, intentional self-harm, subsequent encounter	ICD10CM
**T48.4X2S**	Poisoning by expectorants, intentional self-harm, sequela	ICD10CM
**T48.5X2**	Poisoning by other anti-common-cold drugs, intentional self-harm	ICD10CM
**T48.5X2A**	Poisoning by other anti-common-cold drugs, intentional self-harm, initial encounter	ICD10CM
**T48.5X2D**	Poisoning by other anti-common-cold drugs, intentional self-harm, subsequent encounter	ICD10CM
**T48.5X2S**	Poisoning by other anti-common-cold drugs, intentional self-harm, sequela	ICD10CM
**T48.6X2**	Poisoning by antiasthmatics, intentional self-harm	ICD10CM
**T48.6X2A**	Poisoning by antiasthmatics, intentional self-harm, initial encounter	ICD10CM

**T48.6X2D**	Poisoning by antiasthmatics, intentional self-harm, subsequent encounter	ICD10CM
**T48.6X2S**	Poisoning by antiasthmatics, intentional self-harm, sequela	ICD10CM
**T48.902**	Poisoning by unspecified agents primarily acting on the respiratory system, intentional self-harm	ICD10CM
**T48.902A**	Poisoning by unspecified agents primarily acting on the respiratory system, intentional self-harm, initial encounter	ICD10CM
**T48.902D**	Poisoning by unspecified agents primarily acting on the respiratory system, intentional self-harm, subsequent encounter	ICD10CM
**T48.902S**	Poisoning by unspecified agents primarily acting on the respiratory system, intentional self-harm, sequela	ICD10CM
**T48.992**	Poisoning by other agents primarily acting on the respiratory system, intentional self-harm	ICD10CM
**T48.992A**	Poisoning by other agents primarily acting on the respiratory system, intentional self-harm, initial encounter	ICD10CM
**T48.992D**	Poisoning by other agents primarily acting on the respiratory system, intentional self-harm, subsequent encounter	ICD10CM
**T48.992S**	Poisoning by other agents primarily acting on the respiratory system, intentional self-harm, sequela	ICD10CM
**T49.0X2**	Poisoning by local antifungal, anti-infective and anti-inflammatory drugs, intentional self-harm	ICD10CM
**T49.0X2A**	Poisoning by local antifungal, anti-infective and anti-inflammatory drugs, intentional self-harm, initial encounter	ICD10CM
**T49.0X2D**	Poisoning by local antifungal, anti-infective and anti-inflammatory drugs, intentional self-harm, subsequent encounter	ICD10CM
**T49.0X2S**	Poisoning by local antifungal, anti-infective and anti-inflammatory drugs, intentional self-harm, sequela	ICD10CM
**T49.1X2**	Poisoning by antipruritics, intentional self-harm	ICD10CM
**T49.1X2A**	Poisoning by antipruritics, intentional self-harm, initial encounter	ICD10CM
**T49.1X2D**	Poisoning by antipruritics, intentional self-harm, subsequent encounter	ICD10CM
**T49.1X2S**	Poisoning by antipruritics, intentional self-harm, sequela	ICD10CM
**T49.2X2**	Poisoning by local astringents and local detergents, intentional self-harm	ICD10CM

© 2021 Walsh CG et al. *JAMA Network Open.*

T49.2X2A	Poisoning by local astringents and local detergents, intentional self-harm, initial encounter	ICD10CM
T49.2X2D	Poisoning by local astringents and local detergents, intentional self-harm, subsequent encounter	ICD10CM
T49.2X2S	Poisoning by local astringents and local detergents, intentional self-harm, sequela	ICD10CM
T49.3X2	Poisoning by emollients, demulcents and protectants, intentional self-harm	ICD10CM
T49.3X2A	Poisoning by emollients, demulcents and protectants, intentional self-harm, initial encounter	ICD10CM
T49.3X2D	Poisoning by emollients, demulcents and protectants, intentional self-harm, subsequent encounter	ICD10CM
T49.3X2S	Poisoning by emollients, demulcents and protectants, intentional self-harm, sequela	ICD10CM
T49.4X2	Poisoning by keratolytics, keratoplastics, and other hair treatment drugs and preparations, intentional self-harm	ICD10CM
T49.4X2A	Poisoning by keratolytics, keratoplastics, and other hair treatment drugs and preparations, intentional self-harm, initial encounter	ICD10CM
T49.4X2D	Poisoning by keratolytics, keratoplastics, and other hair treatment drugs and preparations, intentional self-harm, subsequent encounter	ICD10CM
T49.4X2S	Poisoning by keratolytics, keratoplastics, and other hair treatment drugs and preparations, intentional self-harm, sequela	ICD10CM
T49.5X2	Poisoning by ophthalmological drugs and preparations, intentional self-harm	ICD10CM
T49.5X2A	Poisoning by ophthalmological drugs and preparations, intentional self-harm, initial encounter	ICD10CM
T49.5X2D	Poisoning by ophthalmological drugs and preparations, intentional self-harm, subsequent encounter	ICD10CM
T49.5X2S	Poisoning by ophthalmological drugs and preparations, intentional self-harm, sequela	ICD10CM
T49.6X2	Poisoning by otorhinolaryngological drugs and preparations, intentional self-harm	ICD10CM
T49.6X2A	Poisoning by otorhinolaryngological drugs and preparations, intentional self-harm, initial encounter	ICD10CM
T49.6X2D	Poisoning by otorhinolaryngological drugs and preparations, intentional self-harm, subsequent encounter	ICD10CM

© 2021 Walsh CG et al. *JAMA Network Open.*

T49.6X2S	Poisoning by otorhinolaryngological drugs and preparations, intentional self-harm, sequela	ICD10CM
T49.7X2	Poisoning by dental drugs, topically applied, intentional self-harm	ICD10CM
T49.7X2A	Poisoning by dental drugs, topically applied, intentional self-harm, initial encounter	ICD10CM
T49.7X2D	Poisoning by dental drugs, topically applied, intentional self-harm, subsequent encounter	ICD10CM
T49.7X2S	Poisoning by dental drugs, topically applied, intentional self-harm, sequela	ICD10CM
T49.8X2	Poisoning by other topical agents, intentional self-harm	ICD10CM
T49.8X2A	Poisoning by other topical agents, intentional self-harm, initial encounter	ICD10CM
T49.8X2D	Poisoning by other topical agents, intentional self-harm, subsequent encounter	ICD10CM
T49.8X2S	Poisoning by other topical agents, intentional self-harm, sequela	ICD10CM
T49.92	Poisoning by unspecified topical agent, intentional self-harm	ICD10CM
T49.92XA	Poisoning by unspecified topical agent, intentional self-harm, initial encounter	ICD10CM
T49.92XD	Poisoning by unspecified topical agent, intentional self-harm, subsequent encounter	ICD10CM
T49.92XS	Poisoning by unspecified topical agent, intentional self-harm, sequela	ICD10CM
T50.0X2	Poisoning by mineralocorticoids and their antagonists, intentional self-harm	ICD10CM
T50.0X2A	Poisoning by mineralocorticoids and their antagonists, intentional self-harm, initial encounter	ICD10CM
T50.0X2D	Poisoning by mineralocorticoids and their antagonists, intentional self-harm, subsequent encounter	ICD10CM
T50.0X2S	Poisoning by mineralocorticoids and their antagonists, intentional self-harm, sequela	ICD10CM
T50.1X2	Poisoning by loop [high-ceiling] diuretics, intentional self-harm	ICD10CM
T50.1X2A	Poisoning by loop [high-ceiling] diuretics, intentional self-harm, initial encounter	ICD10CM
T50.1X2D	Poisoning by loop [high-ceiling] diuretics, intentional self-harm, subsequent encounter	ICD10CM

© 2021 Walsh CG et al. *JAMA Network Open.*

T50.1X2S	Poisoning by loop [high-ceiling] diuretics, intentional self-harm, sequela	ICD10CM
T50.2X2	Poisoning by carbonic-anhydrase inhibitors, benzothiadiazides and other diuretics, intentional self-harm	ICD10CM
T50.2X2A	Poisoning by carbonic-anhydrase inhibitors, benzothiadiazides and other diuretics, intentional self-harm, initial encounter	ICD10CM
T50.2X2D	Poisoning by carbonic-anhydrase inhibitors, benzothiadiazides and other diuretics, intentional self-harm, subsequent encounter	ICD10CM
T50.2X2S	Poisoning by carbonic-anhydrase inhibitors, benzothiadiazides and other diuretics, intentional self-harm, sequela	ICD10CM
T50.3X2	Poisoning by electrolytic, caloric and water-balance agents, intentional self-harm	ICD10CM
T50.3X2A	Poisoning by electrolytic, caloric and water-balance agents, intentional self-harm, initial encounter	ICD10CM
T50.3X2D	Poisoning by electrolytic, caloric and water-balance agents, intentional self-harm, subsequent encounter	ICD10CM
T50.3X2S	Poisoning by electrolytic, caloric and water-balance agents, intentional self-harm, sequela	ICD10CM
T50.4X2	Poisoning by drugs affecting uric acid metabolism, intentional self-harm	ICD10CM
T50.4X2A	Poisoning by drugs affecting uric acid metabolism, intentional self-harm, initial encounter	ICD10CM
T50.4X2D	Poisoning by drugs affecting uric acid metabolism, intentional self-harm, subsequent encounter	ICD10CM
T50.4X2S	Poisoning by drugs affecting uric acid metabolism, intentional self-harm, sequela	ICD10CM
T50.5X2	Poisoning by appetite depressants, intentional self-harm	ICD10CM
T50.5X2A	Poisoning by appetite depressants, intentional self-harm, initial encounter	ICD10CM
T50.5X2D	Poisoning by appetite depressants, intentional self-harm, subsequent encounter	ICD10CM
T50.5X2S	Poisoning by appetite depressants, intentional self-harm, sequela	ICD10CM
T50.6X2	Poisoning by antidotes and chelating agents, intentional self-harm	ICD10CM
T50.6X2A	Poisoning by antidotes and chelating agents, intentional self-harm, initial encounter	ICD10CM

© 2021 Walsh CG et al. *JAMA Network Open.*

T50.6X2D	Poisoning by antidotes and chelating agents, intentional self-harm, subsequent encounter	ICD10CM
T50.6X2S	Poisoning by antidotes and chelating agents, intentional self-harm, sequela	ICD10CM
T50.7X2	Poisoning by analeptics and opioid receptor antagonists, intentional self-harm	ICD10CM
T50.7X2A	Poisoning by analeptics and opioid receptor antagonists, intentional self-harm, initial encounter	ICD10CM
T50.7X2D	Poisoning by analeptics and opioid receptor antagonists, intentional self-harm, subsequent encounter	ICD10CM
T50.7X2S	Poisoning by analeptics and opioid receptor antagonists, intentional self-harm, sequela	ICD10CM
T50.8X2	Poisoning by diagnostic agents, intentional self-harm	ICD10CM
T50.8X2A	Poisoning by diagnostic agents, intentional self-harm, initial encounter	ICD10CM
T50.8X2D	Poisoning by diagnostic agents, intentional self-harm, subsequent encounter	ICD10CM
T50.8X2S	Poisoning by diagnostic agents, intentional self-harm, sequela	ICD10CM
T50.902	Poisoning by unspecified drugs, medicaments and biological substances, intentional self-harm	ICD10CM
T50.902A	Poisoning by unspecified drugs, medicaments and biological substances, intentional self-harm, initial encounter	ICD10CM
T50.902D	Poisoning by unspecified drugs, medicaments and biological substances, intentional self-harm, subsequent encounter	ICD10CM
T50.902S	Poisoning by unspecified drugs, medicaments and biological substances, intentional self-harm, sequela	ICD10CM
T50.992	Poisoning by other drugs, medicaments and biological substances, intentional self-harm	ICD10CM
T50.992A	Poisoning by other drugs, medicaments and biological substances, intentional self-harm, initial encounter	ICD10CM
T50.992D	Poisoning by other drugs, medicaments and biological substances, intentional self-harm, subsequent encounter	ICD10CM
T50.992S	Poisoning by other drugs, medicaments and biological substances, intentional self-harm, sequela	ICD10CM

© 2021 Walsh CG et al. *JAMA Network Open.*

T50.A12	Poisoning by pertussis vaccine, including combinations with a pertussis component, intentional self-harm	ICD10CM
T50.A12A	Poisoning by pertussis vaccine, including combinations with a pertussis component, intentional self-harm, initial encounter	ICD10CM
T50.A12D	Poisoning by pertussis vaccine, including combinations with a pertussis component, intentional self-harm, subsequent encounter	ICD10CM
T50.A12S	Poisoning by pertussis vaccine, including combinations with a pertussis component, intentional self-harm, sequela	ICD10CM
T50.A22	Poisoning by mixed bacterial vaccines without a pertussis component, intentional self-harm	ICD10CM
T50.A22A	Poisoning by mixed bacterial vaccines without a pertussis component, intentional self-harm, initial encounter	ICD10CM
T50.A22D	Poisoning by mixed bacterial vaccines without a pertussis component, intentional self-harm, subsequent encounter	ICD10CM
T50.A22S	Poisoning by mixed bacterial vaccines without a pertussis component, intentional self-harm, sequela	ICD10CM
T50.A92	Poisoning by other bacterial vaccines, intentional self-harm	ICD10CM
T50.A92A	Poisoning by other bacterial vaccines, intentional self-harm, initial encounter	ICD10CM
T50.A92D	Poisoning by other bacterial vaccines, intentional self-harm, subsequent encounter	ICD10CM
T50.A92S	Poisoning by other bacterial vaccines, intentional self-harm, sequela	ICD10CM
T50.B12	Poisoning by smallpox vaccines, intentional self-harm	ICD10CM
T50.B12A	Poisoning by smallpox vaccines, intentional self-harm, initial encounter	ICD10CM
T50.B12D	Poisoning by smallpox vaccines, intentional self-harm, subsequent encounter	ICD10CM
T50.B12S	Poisoning by smallpox vaccines, intentional self-harm, sequela	ICD10CM
T50.B92	Poisoning by other viral vaccines, intentional self-harm	ICD10CM
T50.B92A	Poisoning by other viral vaccines, intentional self-harm, initial encounter	ICD10CM
T50.B92D	Poisoning by other viral vaccines, intentional self-harm, subsequent encounter	ICD10CM
T50.B92S	Poisoning by other viral vaccines, intentional self-harm, sequela	ICD10CM

© 2021 Walsh CG et al. *JAMA Network Open.*

**T50.Z12**	Poisoning by immunoglobulin, intentional self-harm	ICD10CM
**T50.Z12A**	Poisoning by immunoglobulin, intentional self-harm, initial encounter	ICD10CM
**T50.Z12D**	Poisoning by immunoglobulin, intentional self-harm, subsequent encounter	ICD10CM
**T50.Z12S**	Poisoning by immunoglobulin, intentional self-harm, sequela	ICD10CM
**T50.Z92**	Poisoning by other vaccines and biological substances, intentional self-harm	ICD10CM
**T50.Z92A**	Poisoning by other vaccines and biological substances, intentional self-harm, initial encounter	ICD10CM
**T50.Z92D**	Poisoning by other vaccines and biological substances, intentional self-harm, subsequent encounter	ICD10CM
**T50.Z92S**	Poisoning by other vaccines and biological substances, intentional self-harm, sequela	ICD10CM
**T51.0X2**	Toxic effect of ethanol, intentional self-harm	ICD10CM
**T51.0X2A**	Toxic effect of ethanol, intentional self-harm, initial encounter	ICD10CM
**T51.0X2D**	Toxic effect of ethanol, intentional self-harm, subsequent encounter	ICD10CM
**T51.0X2S**	Toxic effect of ethanol, intentional self-harm, sequela	ICD10CM
**T51.1X2**	Toxic effect of methanol, intentional self-harm	ICD10CM
**T51.1X2A**	Toxic effect of methanol, intentional self-harm, initial encounter	ICD10CM
**T51.1X2D**	Toxic effect of methanol, intentional self-harm, subsequent encounter	ICD10CM
**T51.1X2S**	Toxic effect of methanol, intentional self-harm, sequela	ICD10CM
**T51.2X2**	Toxic effect of 2-Propanol, intentional self-harm	ICD10CM
**T51.2X2A**	Toxic effect of 2-Propanol, intentional self-harm, initial encounter	ICD10CM
**T51.2X2D**	Toxic effect of 2-Propanol, intentional self-harm, subsequent encounter	ICD10CM
**T51.2X2S**	Toxic effect of 2-Propanol, intentional self-harm, sequela	ICD10CM
**T51.3X2**	Toxic effect of fusel oil, intentional self-harm	ICD10CM

© 2021 Walsh CG et al. *JAMA Network Open.*

T51.3X2A	Toxic effect of fusel oil, intentional self-harm, initial encounter	ICD10CM
T51.3X2D	Toxic effect of fusel oil, intentional self-harm, subsequent encounter	ICD10CM
T51.3X2S	Toxic effect of fusel oil, intentional self-harm, sequela	ICD10CM
T51.8X2	Toxic effect of other alcohols, intentional self-harm	ICD10CM
T51.8X2A	Toxic effect of other alcohols, intentional self-harm, initial encounter	ICD10CM
T51.8X2D	Toxic effect of other alcohols, intentional self-harm, subsequent encounter	ICD10CM
T51.8X2S	Toxic effect of other alcohols, intentional self-harm, sequela	ICD10CM
T51.92	Toxic effect of unspecified alcohol, intentional self-harm	ICD10CM
T51.92XA	Toxic effect of unspecified alcohol, intentional self-harm, initial encounter	ICD10CM
T51.92XD	Toxic effect of unspecified alcohol, intentional self-harm, subsequent encounter	ICD10CM
T51.92XS	Toxic effect of unspecified alcohol, intentional self-harm, sequela	ICD10CM
T52.0X2	Toxic effect of petroleum products, intentional self-harm	ICD10CM
T52.0X2A	Toxic effect of petroleum products, intentional self-harm, initial encounter	ICD10CM
T52.0X2D	Toxic effect of petroleum products, intentional self-harm, subsequent encounter	ICD10CM
T52.0X2S	Toxic effect of petroleum products, intentional self-harm, sequela	ICD10CM
T52.1X2	Toxic effect of benzene, intentional self-harm	ICD10CM
T52.1X2A	Toxic effect of benzene, intentional self-harm, initial encounter	ICD10CM
T52.1X2D	Toxic effect of benzene, intentional self-harm, subsequent encounter	ICD10CM
T52.1X2S	Toxic effect of benzene, intentional self-harm, sequela	ICD10CM
T52.2X2	Toxic effect of homologues of benzene, intentional self-harm	ICD10CM
T52.2X2A	Toxic effect of homologues of benzene, intentional self-harm, initial encounter	ICD10CM
T52.2X2D	Toxic effect of homologues of benzene, intentional self-harm, subsequent encounter	ICD10CM

T52.2X2S	Toxic effect of homologues of benzene, intentional self-harm, sequela	ICD10CM
T52.3X2	Toxic effect of glycols, intentional self-harm	ICD10CM
T52.3X2A	Toxic effect of glycols, intentional self-harm, initial encounter	ICD10CM
T52.3X2D	Toxic effect of glycols, intentional self-harm, subsequent encounter	ICD10CM
T52.3X2S	Toxic effect of glycols, intentional self-harm, sequela	ICD10CM
T52.4X2	Toxic effect of ketones, intentional self-harm	ICD10CM
T52.4X2A	Toxic effect of ketones, intentional self-harm, initial encounter	ICD10CM
T52.4X2D	Toxic effect of ketones, intentional self-harm, subsequent encounter	ICD10CM
T52.4X2S	Toxic effect of ketones, intentional self-harm, sequela	ICD10CM
T52.8X2	Toxic effect of other organic solvents, intentional self-harm	ICD10CM
T52.8X2A	Toxic effect of other organic solvents, intentional self-harm, initial encounter	ICD10CM
T52.8X2D	Toxic effect of other organic solvents, intentional self-harm, subsequent encounter	ICD10CM
T52.8X2S	Toxic effect of other organic solvents, intentional self-harm, sequela	ICD10CM
T52.92	Toxic effect of unspecified organic solvent, intentional self-harm	ICD10CM
T52.92XA	Toxic effect of unspecified organic solvent, intentional self-harm, initial encounter	ICD10CM
T52.92XD	Toxic effect of unspecified organic solvent, intentional self-harm, subsequent encounter	ICD10CM
T52.92XS	Toxic effect of unspecified organic solvent, intentional self-harm, sequela	ICD10CM
T53.0X2	Toxic effect of carbon tetrachloride, intentional self-harm	ICD10CM
T53.0X2A	Toxic effect of carbon tetrachloride, intentional self-harm, initial encounter	ICD10CM
T53.0X2D	Toxic effect of carbon tetrachloride, intentional self-harm, subsequent encounter	ICD10CM
T53.0X2S	Toxic effect of carbon tetrachloride, intentional self-harm, sequela	ICD10CM
T53.1X2	Toxic effect of chloroform, intentional self-harm	ICD10CM

© 2021 Walsh CG et al. *JAMA Network Open.*

T53.1X2A	Toxic effect of chloroform, intentional self-harm, initial encounter	ICD10CM
T53.1X2D	Toxic effect of chloroform, intentional self-harm, subsequent encounter	ICD10CM
T53.1X2S	Toxic effect of chloroform, intentional self-harm, sequela	ICD10CM
T53.2X2	Toxic effect of trichloroethylene, intentional self-harm	ICD10CM
T53.2X2A	Toxic effect of trichloroethylene, intentional self-harm, initial encounter	ICD10CM
T53.2X2D	Toxic effect of trichloroethylene, intentional self-harm, subsequent encounter	ICD10CM
T53.2X2S	Toxic effect of trichloroethylene, intentional self-harm, sequela	ICD10CM
T53.3X2	Toxic effect of tetrachloroethylene, intentional self-harm	ICD10CM
T53.3X2A	Toxic effect of tetrachloroethylene, intentional self-harm, initial encounter	ICD10CM
T53.3X2D	Toxic effect of tetrachloroethylene, intentional self-harm, subsequent encounter	ICD10CM
T53.3X2S	Toxic effect of tetrachloroethylene, intentional self-harm, sequela	ICD10CM
T53.4X2	Toxic effect of dichloromethane, intentional self-harm	ICD10CM
T53.4X2A	Toxic effect of dichloromethane, intentional self-harm, initial encounter	ICD10CM
T53.4X2D	Toxic effect of dichloromethane, intentional self-harm, subsequent encounter	ICD10CM
T53.4X2S	Toxic effect of dichloromethane, intentional self-harm, sequela	ICD10CM
T53.5X2	Toxic effect of chlorofluorocarbons, intentional self-harm	ICD10CM
T53.5X2A	Toxic effect of chlorofluorocarbons, intentional self-harm, initial encounter	ICD10CM
T53.5X2D	Toxic effect of chlorofluorocarbons, intentional self-harm, subsequent encounter	ICD10CM
T53.5X2S	Toxic effect of chlorofluorocarbons, intentional self-harm, sequela	ICD10CM
T53.6X2	Toxic effect of other halogen derivatives of aliphatic hydrocarbons, intentional self-harm	ICD10CM
T53.6X2A	Toxic effect of other halogen derivatives of aliphatic hydrocarbons, intentional self-harm, initial encounter	ICD10CM

© 2021 Walsh CG et al. *JAMA Network Open.*

T53.6X2D	Toxic effect of other halogen derivatives of aliphatic hydrocarbons, intentional self-harm, subsequent encounter	ICD10CM
T53.6X2S	Toxic effect of other halogen derivatives of aliphatic hydrocarbons, intentional self-harm, sequela	ICD10CM
T53.7X2	Toxic effect of other halogen derivatives of aromatic hydrocarbons, intentional self-harm	ICD10CM
T53.7X2A	Toxic effect of other halogen derivatives of aromatic hydrocarbons, intentional self-harm, initial encounter	ICD10CM
T53.7X2D	Toxic effect of other halogen derivatives of aromatic hydrocarbons, intentional self-harm, subsequent encounter	ICD10CM
T53.7X2S	Toxic effect of other halogen derivatives of aromatic hydrocarbons, intentional self-harm, sequela	ICD10CM
T53.92	Toxic effect of unspecified halogen derivatives of aliphatic and aromatic hydrocarbons, intentional self-harm	ICD10CM
T53.92XA	Toxic effect of unspecified halogen derivatives of aliphatic and aromatic hydrocarbons, intentional self-harm, initial encounter	ICD10CM
T53.92XD	Toxic effect of unspecified halogen derivatives of aliphatic and aromatic hydrocarbons, intentional self-harm, subsequent encounter	ICD10CM
T53.92XS	Toxic effect of unspecified halogen derivatives of aliphatic and aromatic hydrocarbons, intentional self-harm, sequela	ICD10CM
T54.0X2	Toxic effect of phenol and phenol homologues, intentional self-harm	ICD10CM
T54.0X2A	Toxic effect of phenol and phenol homologues, intentional self-harm, initial encounter	ICD10CM
T54.0X2D	Toxic effect of phenol and phenol homologues, intentional self-harm, subsequent encounter	ICD10CM
T54.0X2S	Toxic effect of phenol and phenol homologues, intentional self-harm, sequela	ICD10CM
T54.1X2	Toxic effect of other corrosive organic compounds, intentional self-harm	ICD10CM
T54.1X2A	Toxic effect of other corrosive organic compounds, intentional self-harm, initial encounter	ICD10CM
T54.1X2D	Toxic effect of other corrosive organic compounds, intentional self-harm, subsequent encounter	ICD10CM
T54.1X2S	Toxic effect of other corrosive organic compounds, intentional self-harm, sequela	ICD10CM

© 2021 Walsh CG et al. *JAMA Network Open.*

T54.2X2	Toxic effect of corrosive acids and acid-like substances, intentional self-harm	ICD10CM
T54.2X2A	Toxic effect of corrosive acids and acid-like substances, intentional self-harm, initial encounter	ICD10CM
T54.2X2D	Toxic effect of corrosive acids and acid-like substances, intentional self-harm, subsequent encounter	ICD10CM
T54.2X2S	Toxic effect of corrosive acids and acid-like substances, intentional self-harm, sequela	ICD10CM
T54.3X2	Toxic effect of corrosive alkalis and alkali-like substances, intentional self-harm	ICD10CM
T54.3X2A	Toxic effect of corrosive alkalis and alkali-like substances, intentional self-harm, initial encounter	ICD10CM
T54.3X2D	Toxic effect of corrosive alkalis and alkali-like substances, intentional self-harm, subsequent encounter	ICD10CM
T54.3X2S	Toxic effect of corrosive alkalis and alkali-like substances, intentional self-harm, sequela	ICD10CM
T54.92	Toxic effect of unspecified corrosive substance, intentional self-harm	ICD10CM
T54.92XA	Toxic effect of unspecified corrosive substance, intentional self-harm, initial encounter	ICD10CM
T54.92XD	Toxic effect of unspecified corrosive substance, intentional self-harm, subsequent encounter	ICD10CM
T54.92XS	Toxic effect of unspecified corrosive substance, intentional self-harm, sequela	ICD10CM
T55.0X2	Toxic effect of soaps, intentional self-harm	ICD10CM
T55.0X2A	Toxic effect of soaps, intentional self-harm, initial encounter	ICD10CM
T55.0X2D	Toxic effect of soaps, intentional self-harm, subsequent encounter	ICD10CM
T55.0X2S	Toxic effect of soaps, intentional self-harm, sequela	ICD10CM
T55.1X2	Toxic effect of detergents, intentional self-harm	ICD10CM
T55.1X2A	Toxic effect of detergents, intentional self-harm, initial encounter	ICD10CM
T55.1X2D	Toxic effect of detergents, intentional self-harm, subsequent encounter	ICD10CM
T55.1X2S	Toxic effect of detergents, intentional self-harm, sequela	ICD10CM

© 2021 Walsh CG et al. *JAMA Network Open.*

T56.0X2	Toxic effect of lead and its compounds, intentional self-harm	ICD10CM
T56.0X2A	Toxic effect of lead and its compounds, intentional self-harm, initial encounter	ICD10CM
T56.0X2D	Toxic effect of lead and its compounds, intentional self-harm, subsequent encounter	ICD10CM
T56.0X2S	Toxic effect of lead and its compounds, intentional self-harm, sequela	ICD10CM
T56.1X2	Toxic effect of mercury and its compounds, intentional self-harm	ICD10CM
T56.1X2A	Toxic effect of mercury and its compounds, intentional self-harm, initial encounter	ICD10CM
T56.1X2D	Toxic effect of mercury and its compounds, intentional self-harm, subsequent encounter	ICD10CM
T56.1X2S	Toxic effect of mercury and its compounds, intentional self-harm, sequela	ICD10CM
T56.2X2	Toxic effect of chromium and its compounds, intentional self-harm	ICD10CM
T56.2X2A	Toxic effect of chromium and its compounds, intentional self-harm, initial encounter	ICD10CM
T56.2X2D	Toxic effect of chromium and its compounds, intentional self-harm, subsequent encounter	ICD10CM
T56.2X2S	Toxic effect of chromium and its compounds, intentional self-harm, sequela	ICD10CM
T56.3X2	Toxic effect of cadmium and its compounds, intentional self-harm	ICD10CM
T56.3X2A	Toxic effect of cadmium and its compounds, intentional self-harm, initial encounter	ICD10CM
T56.3X2D	Toxic effect of cadmium and its compounds, intentional self-harm, subsequent encounter	ICD10CM
T56.3X2S	Toxic effect of cadmium and its compounds, intentional self-harm, sequela	ICD10CM
T56.4X2	Toxic effect of copper and its compounds, intentional self-harm	ICD10CM
T56.4X2A	Toxic effect of copper and its compounds, intentional self-harm, initial encounter	ICD10CM
T56.4X2D	Toxic effect of copper and its compounds, intentional self-harm, subsequent encounter	ICD10CM
T56.4X2S	Toxic effect of copper and its compounds, intentional self-harm, sequela	ICD10CM
T56.5X2	Toxic effect of zinc and its compounds, intentional self-harm	ICD10CM

© 2021 Walsh CG et al. *JAMA Network Open.*

**T56.5X2A**	Toxic effect of zinc and its compounds, intentional self-harm, initial encounter	ICD10CM
**T56.5X2D**	Toxic effect of zinc and its compounds, intentional self-harm, subsequent encounter	ICD10CM
**T56.5X2S**	Toxic effect of zinc and its compounds, intentional self-harm, sequela	ICD10CM
**T56.6X2**	Toxic effect of tin and its compounds, intentional self-harm	ICD10CM
**T56.6X2A**	Toxic effect of tin and its compounds, intentional self-harm, initial encounter	ICD10CM
**T56.6X2D**	Toxic effect of tin and its compounds, intentional self-harm, subsequent encounter	ICD10CM
**T56.6X2S**	Toxic effect of tin and its compounds, intentional self-harm, sequela	ICD10CM
**T56.7X2**	Toxic effect of beryllium and its compounds, intentional self-harm	ICD10CM
**T56.7X2A**	Toxic effect of beryllium and its compounds, intentional self-harm, initial encounter	ICD10CM
**T56.7X2D**	Toxic effect of beryllium and its compounds, intentional self-harm, subsequent encounter	ICD10CM
**T56.7X2S**	Toxic effect of beryllium and its compounds, intentional self-harm, sequela	ICD10CM
**T56.812**	Toxic effect of thallium, intentional self-harm	ICD10CM
**T56.812A**	Toxic effect of thallium, intentional self-harm, initial encounter	ICD10CM
**T56.812D**	Toxic effect of thallium, intentional self-harm, subsequent encounter	ICD10CM
**T56.812S**	Toxic effect of thallium, intentional self-harm, sequela	ICD10CM
**T56.892**	Toxic effect of other metals, intentional self-harm	ICD10CM
**T56.892A**	Toxic effect of other metals, intentional self-harm, initial encounter	ICD10CM
**T56.892D**	Toxic effect of other metals, intentional self-harm, subsequent encounter	ICD10CM
**T56.892S**	Toxic effect of other metals, intentional self-harm, sequela	ICD10CM
**T56.8x2A**	Toxic effect of other metals, intentional self-harm, initial encounter	ICD10CM
**T56.8x2D**	Toxic effect of other metals, intentional self-harm, subsequent encounter	ICD10CM
**T56.8x2S**	Toxic effect of other metals, intentional self-harm, sequela	ICD10CM

© 2021 Walsh CG et al. *JAMA Network Open.*

T56.92	Toxic effect of unspecified metal, intentional self-harm	ICD10CM
T56.92XA	Toxic effect of unspecified metal, intentional self-harm, initial encounter	ICD10CM
T56.92XD	Toxic effect of unspecified metal, intentional self-harm, subsequent encounter	ICD10CM
T56.92XS	Toxic effect of unspecified metal, intentional self-harm, sequela	ICD10CM
T57.0X2	Toxic effect of arsenic and its compounds, intentional self-harm	ICD10CM
T57.0X2A	Toxic effect of arsenic and its compounds, intentional self-harm, initial encounter	ICD10CM
T57.0X2D	Toxic effect of arsenic and its compounds, intentional self-harm, subsequent encounter	ICD10CM
T57.0X2S	Toxic effect of arsenic and its compounds, intentional self-harm, sequela	ICD10CM
T57.1X2	Toxic effect of phosphorus and its compounds, intentional self-harm	ICD10CM
T57.1X2A	Toxic effect of phosphorus and its compounds, intentional self-harm, initial encounter	ICD10CM
T57.1X2D	Toxic effect of phosphorus and its compounds, intentional self-harm, subsequent encounter	ICD10CM
T57.1X2S	Toxic effect of phosphorus and its compounds, intentional self-harm, sequela	ICD10CM
T57.2X2	Toxic effect of manganese and its compounds, intentional self-harm	ICD10CM
T57.2X2A	Toxic effect of manganese and its compounds, intentional self-harm, initial encounter	ICD10CM
T57.2X2D	Toxic effect of manganese and its compounds, intentional self-harm, subsequent encounter	ICD10CM
T57.2X2S	Toxic effect of manganese and its compounds, intentional self-harm, sequela	ICD10CM
T57.3X2	Toxic effect of hydrogen cyanide, intentional self-harm	ICD10CM
T57.3X2A	Toxic effect of hydrogen cyanide, intentional self-harm, initial encounter	ICD10CM
T57.3X2D	Toxic effect of hydrogen cyanide, intentional self-harm, subsequent encounter	ICD10CM
T57.3X2S	Toxic effect of hydrogen cyanide, intentional self-harm, sequela	ICD10CM
T57.8X2	Toxic effect of other specified inorganic substances, intentional self-harm	ICD10CM

© 2021 Walsh CG et al. *JAMA Network Open.*

T57.8X2A	Toxic effect of other specified inorganic substances, intentional self-harm, initial encounter	ICD10CM
T57.8X2D	Toxic effect of other specified inorganic substances, intentional self-harm, subsequent encounter	ICD10CM
T57.8X2S	Toxic effect of other specified inorganic substances, intentional self-harm, sequela	ICD10CM
T57.92	Toxic effect of unspecified inorganic substance, intentional self-harm	ICD10CM
T57.92XA	Toxic effect of unspecified inorganic substance, intentional self-harm, initial encounter	ICD10CM
T57.92XD	Toxic effect of unspecified inorganic substance, intentional self-harm, subsequent encounter	ICD10CM
T57.92XS	Toxic effect of unspecified inorganic substance, intentional self-harm, sequela	ICD10CM
T58.02	Toxic effect of carbon monoxide from motor vehicle exhaust, intentional self-harm	ICD10CM
T58.02XA	Toxic effect of carbon monoxide from motor vehicle exhaust, intentional self-harm, initial encounter	ICD10CM
T58.02XD	Toxic effect of carbon monoxide from motor vehicle exhaust, intentional self-harm, subsequent encounter	ICD10CM
T58.02XS	Toxic effect of carbon monoxide from motor vehicle exhaust, intentional self-harm, sequela	ICD10CM
T58.12	Toxic effect of carbon monoxide from utility gas, intentional self-harm	ICD10CM
T58.12XA	Toxic effect of carbon monoxide from utility gas, intentional self-harm, initial encounter	ICD10CM
T58.12XD	Toxic effect of carbon monoxide from utility gas, intentional self-harm, subsequent encounter	ICD10CM
T58.12XS	Toxic effect of carbon monoxide from utility gas, intentional self-harm, sequela	ICD10CM
T58.2X2	Toxic effect of carbon monoxide from incomplete combustion of other domestic fuels, intentional self-harm	ICD10CM
T58.2X2A	Toxic effect of carbon monoxide from incomplete combustion of other domestic fuels, intentional self-harm, initial encounter	ICD10CM
T58.2X2D	Toxic effect of carbon monoxide from incomplete combustion of other domestic fuels, intentional self-harm, subsequent encounter	ICD10CM

© 2021 Walsh CG et al. *JAMA Network Open.*

T58.2X2S	Toxic effect of carbon monoxide from incomplete combustion of other domestic fuels, intentional self-harm, sequela	ICD10CM
T58.8X2	Toxic effect of carbon monoxide from other source, intentional self-harm	ICD10CM
T58.8X2A	Toxic effect of carbon monoxide from other source, intentional self-harm, initial encounter	ICD10CM
T58.8X2D	Toxic effect of carbon monoxide from other source, intentional self-harm, subsequent encounter	ICD10CM
T58.8X2S	Toxic effect of carbon monoxide from other source, intentional self-harm, sequela	ICD10CM
T58.92	Toxic effect of carbon monoxide from unspecified source, intentional self-harm	ICD10CM
T58.92XA	Toxic effect of carbon monoxide from unspecified source, intentional self-harm, initial encounter	ICD10CM
T58.92XD	Toxic effect of carbon monoxide from unspecified source, intentional self-harm, subsequent encounter	ICD10CM
T58.92XS	Toxic effect of carbon monoxide from unspecified source, intentional self-harm, sequela	ICD10CM
T59.0X2	Toxic effect of nitrogen oxides, intentional self-harm	ICD10CM
T59.0X2A	Toxic effect of nitrogen oxides, intentional self-harm, initial encounter	ICD10CM
T59.0X2D	Toxic effect of nitrogen oxides, intentional self-harm, subsequent encounter	ICD10CM
T59.0X2S	Toxic effect of nitrogen oxides, intentional self-harm, sequela	ICD10CM
T59.1X2	Toxic effect of sulfur dioxide, intentional self-harm	ICD10CM
T59.1X2A	Toxic effect of sulfur dioxide, intentional self-harm, initial encounter	ICD10CM
T59.1X2D	Toxic effect of sulfur dioxide, intentional self-harm, subsequent encounter	ICD10CM
T59.1X2S	Toxic effect of sulfur dioxide, intentional self-harm, sequela	ICD10CM
T59.2X2	Toxic effect of formaldehyde, intentional self-harm	ICD10CM
T59.2X2A	Toxic effect of formaldehyde, intentional self-harm, initial encounter	ICD10CM
T59.2X2D	Toxic effect of formaldehyde, intentional self-harm, subsequent encounter	ICD10CM

© 2021 Walsh CG et al. *JAMA Network Open.*

T59.2X2S	Toxic effect of formaldehyde, intentional self-harm, sequela	ICD10CM
T59.3X2	Toxic effect of lacrimogenic gas, intentional self-harm	ICD10CM
T59.3X2A	Toxic effect of lacrimogenic gas, intentional self-harm, initial encounter	ICD10CM
T59.3X2D	Toxic effect of lacrimogenic gas, intentional self-harm, subsequent encounter	ICD10CM
T59.3X2S	Toxic effect of lacrimogenic gas, intentional self-harm, sequela	ICD10CM
T59.4X2	Toxic effect of chlorine gas, intentional self-harm	ICD10CM
T59.4X2A	Toxic effect of chlorine gas, intentional self-harm, initial encounter	ICD10CM
T59.4X2D	Toxic effect of chlorine gas, intentional self-harm, subsequent encounter	ICD10CM
T59.4X2S	Toxic effect of chlorine gas, intentional self-harm, sequela	ICD10CM
T59.5X2	Toxic effect of fluorine gas and hydrogen fluoride, intentional self-harm	ICD10CM
T59.5X2A	Toxic effect of fluorine gas and hydrogen fluoride, intentional self-harm, initial encounter	ICD10CM
T59.5X2D	Toxic effect of fluorine gas and hydrogen fluoride, intentional self-harm, subsequent encounter	ICD10CM
T59.5X2S	Toxic effect of fluorine gas and hydrogen fluoride, intentional self-harm, sequela	ICD10CM
T59.6X2	Toxic effect of hydrogen sulfide, intentional self-harm	ICD10CM
T59.6X2A	Toxic effect of hydrogen sulfide, intentional self-harm, initial encounter	ICD10CM
T59.6X2D	Toxic effect of hydrogen sulfide, intentional self-harm, subsequent encounter	ICD10CM
T59.6X2S	Toxic effect of hydrogen sulfide, intentional self-harm, sequela	ICD10CM
T59.7X2	Toxic effect of carbon dioxide, intentional self-harm	ICD10CM
T59.7X2A	Toxic effect of carbon dioxide, intentional self-harm, initial encounter	ICD10CM
T59.7X2D	Toxic effect of carbon dioxide, intentional self-harm, subsequent encounter	ICD10CM
T59.7X2S	Toxic effect of carbon dioxide, intentional self-harm, sequela	ICD10CM
T59.812	Toxic effect of smoke, intentional self-harm	ICD10CM

© 2021 Walsh CG et al. *JAMA Network Open.*

T59.812A	Toxic effect of smoke, intentional self-harm, initial encounter	ICD10CM
T59.812D	Toxic effect of smoke, intentional self-harm, subsequent encounter	ICD10CM
T59.812S	Toxic effect of smoke, intentional self-harm, sequela	ICD10CM
T59.892	Toxic effect of other specified gases, fumes and vapors, intentional self-harm	ICD10CM
T59.892A	Toxic effect of other specified gases, fumes and vapors, intentional self-harm, initial encounter	ICD10CM
T59.892D	Toxic effect of other specified gases, fumes and vapors, intentional self-harm, subsequent encounter	ICD10CM
T59.892S	Toxic effect of other specified gases, fumes and vapors, intentional self-harm, sequela	ICD10CM
T59.92	Toxic effect of unspecified gases, fumes and vapors, intentional self-harm	ICD10CM
T59.92XA	Toxic effect of unspecified gases, fumes and vapors, intentional self-harm, initial encounter	ICD10CM
T59.92XD	Toxic effect of unspecified gases, fumes and vapors, intentional self-harm, subsequent encounter	ICD10CM
T59.92XS	Toxic effect of unspecified gases, fumes and vapors, intentional self-harm, sequela	ICD10CM
T60.0X2	Toxic effect of organophosphate and carbamate insecticides, intentional self-harm	ICD10CM
T60.0X2A	Toxic effect of organophosphate and carbamate insecticides, intentional self-harm, initial encounter	ICD10CM
T60.0X2D	Toxic effect of organophosphate and carbamate insecticides, intentional self-harm, subsequent encounter	ICD10CM
T60.0X2S	Toxic effect of organophosphate and carbamate insecticides, intentional self-harm, sequela	ICD10CM
T60.1X2	Toxic effect of halogenated insecticides, intentional self-harm	ICD10CM
T60.1X2A	Toxic effect of halogenated insecticides, intentional self-harm, initial encounter	ICD10CM
T60.1X2D	Toxic effect of halogenated insecticides, intentional self-harm, subsequent encounter	ICD10CM
T60.1X2S	Toxic effect of halogenated insecticides, intentional self-harm, sequela	ICD10CM
T60.2X2	Toxic effect of other insecticides, intentional self-harm	ICD10CM

© 2021 Walsh CG et al. *JAMA Network Open.*

T60.2X2A	Toxic effect of other insecticides, intentional self-harm, initial encounter	ICD10CM
T60.2X2D	Toxic effect of other insecticides, intentional self-harm, subsequent encounter	ICD10CM
T60.2X2S	Toxic effect of other insecticides, intentional self-harm, sequela	ICD10CM
T60.3X2	Toxic effect of herbicides and fungicides, intentional self-harm	ICD10CM
T60.3X2A	Toxic effect of herbicides and fungicides, intentional self-harm, initial encounter	ICD10CM
T60.3X2D	Toxic effect of herbicides and fungicides, intentional self-harm, subsequent encounter	ICD10CM
T60.3X2S	Toxic effect of herbicides and fungicides, intentional self-harm, sequela	ICD10CM
T60.4X2	Toxic effect of rodenticides, intentional self-harm	ICD10CM
T60.4X2A	Toxic effect of rodenticides, intentional self-harm, initial encounter	ICD10CM
T60.4X2D	Toxic effect of rodenticides, intentional self-harm, subsequent encounter	ICD10CM
T60.4X2S	Toxic effect of rodenticides, intentional self-harm, sequela	ICD10CM
T60.8X2	Toxic effect of other pesticides, intentional self-harm	ICD10CM
T60.8X2A	Toxic effect of other pesticides, intentional self-harm, initial encounter	ICD10CM
T60.8X2D	Toxic effect of other pesticides, intentional self-harm, subsequent encounter	ICD10CM
T60.8X2S	Toxic effect of other pesticides, intentional self-harm, sequela	ICD10CM
T60.92	Toxic effect of unspecified pesticide, intentional self-harm	ICD10CM
T60.92XA	Toxic effect of unspecified pesticide, intentional self-harm, initial encounter	ICD10CM
T60.92XD	Toxic effect of unspecified pesticide, intentional self-harm, subsequent encounter	ICD10CM
T60.92XS	Toxic effect of unspecified pesticide, intentional self-harm, sequela	ICD10CM
T61.02	Ciguatera fish poisoning, intentional self-harm	ICD10CM
T61.02XA	Ciguatera fish poisoning, intentional self-harm, initial encounter	ICD10CM
T61.02XD	Ciguatera fish poisoning, intentional self-harm, subsequent encounter	ICD10CM

**T61.02XS**	Ciguatera fish poisoning, intentional self-harm, sequela	ICD10CM
**T61.12**	Scombroid fish poisoning, intentional self-harm	ICD10CM
**T61.12XA**	Scombroid fish poisoning, intentional self-harm, initial encounter	ICD10CM
**T61.12XD**	Scombroid fish poisoning, intentional self-harm, subsequent encounter	ICD10CM
**T61.12XS**	Scombroid fish poisoning, intentional self-harm, sequela	ICD10CM
**T61.772**	Other fish poisoning, intentional self-harm	ICD10CM
**T61.772A**	Other fish poisoning, intentional self-harm, initial encounter	ICD10CM
**T61.772D**	Other fish poisoning, intentional self-harm, subsequent encounter	ICD10CM
**T61.772S**	Other fish poisoning, intentional self-harm, sequela	ICD10CM
**T61.782**	Other shellfish poisoning, intentional self-harm	ICD10CM
**T61.782A**	Other shellfish poisoning, intentional self-harm, initial encounter	ICD10CM
**T61.782D**	Other shellfish poisoning, intentional self-harm, subsequent encounter	ICD10CM
**T61.782S**	Other shellfish poisoning, intentional self-harm, sequela	ICD10CM
**T61.8X2**	Toxic effect of other seafood, intentional self-harm	ICD10CM
**T61.8X2A**	Toxic effect of other seafood, intentional self-harm, initial encounter	ICD10CM
**T61.8X2D**	Toxic effect of other seafood, intentional self-harm, subsequent encounter	ICD10CM
**T61.8X2S**	Toxic effect of other seafood, intentional self-harm, sequela	ICD10CM
**T61.92**	Toxic effect of unspecified seafood, intentional self-harm	ICD10CM
**T61.92XA**	Toxic effect of unspecified seafood, intentional self-harm, initial encounter	ICD10CM
**T61.92XD**	Toxic effect of unspecified seafood, intentional self-harm, subsequent encounter	ICD10CM
**T61.92XS**	Toxic effect of unspecified seafood, intentional self-harm, sequela	ICD10CM
**T62.0X2**	Toxic effect of ingested mushrooms, intentional self-harm	ICD10CM

© 2021 Walsh CG et al. *JAMA Network Open.*

**T62.0X2A**	Toxic effect of ingested mushrooms, intentional self-harm, initial encounter	ICD10CM
**T62.0X2D**	Toxic effect of ingested mushrooms, intentional self-harm, subsequent encounter	ICD10CM
**T62.0X2S**	Toxic effect of ingested mushrooms, intentional self-harm, sequela	ICD10CM
**T62.1X2**	Toxic effect of ingested berries, intentional self-harm	ICD10CM
**T62.1X2A**	Toxic effect of ingested berries, intentional self-harm, initial encounter	ICD10CM
**T62.1X2D**	Toxic effect of ingested berries, intentional self-harm, subsequent encounter	ICD10CM
**T62.1X2S**	Toxic effect of ingested berries, intentional self-harm, sequela	ICD10CM
**T62.2X2**	Toxic effect of other ingested (parts of) plant(s), intentional self-harm	ICD10CM
**T62.2X2A**	Toxic effect of other ingested (parts of) plant(s), intentional self-harm, initial encounter	ICD10CM
**T62.2X2D**	Toxic effect of other ingested (parts of) plant(s), intentional self-harm, subsequent encounter	ICD10CM
**T62.2X2S**	Toxic effect of other ingested (parts of) plant(s), intentional self-harm, sequela	ICD10CM
**T62.8X2**	Toxic effect of other specified noxious substances eaten as food, intentional self-harm	ICD10CM
**T62.8X2A**	Toxic effect of other specified noxious substances eaten as food, intentional self-harm, initial encounter	ICD10CM
**T62.8X2D**	Toxic effect of other specified noxious substances eaten as food, intentional self-harm, subsequent encounter	ICD10CM
**T62.8X2S**	Toxic effect of other specified noxious substances eaten as food, intentional self-harm, sequela	ICD10CM
**T62.92**	Toxic effect of unspecified noxious substance eaten as food, intentional self-harm	ICD10CM
**T62.92XA**	Toxic effect of unspecified noxious substance eaten as food, intentional self-harm, initial encounter	ICD10CM
**T62.92XD**	Toxic effect of unspecified noxious substance eaten as food, intentional self-harm, subsequent encounter	ICD10CM
**T62.92XS**	Toxic effect of unspecified noxious substance eaten as food, intentional self-harm, sequela	ICD10CM
**T63.002**	Toxic effect of unspecified snake venom, intentional self-harm	ICD10CM

© 2021 Walsh CG et al. *JAMA Network Open.*

T63.002A	Toxic effect of unspecified snake venom, intentional self-harm, initial encounter	ICD10CM
T63.002D	Toxic effect of unspecified snake venom, intentional self-harm, subsequent encounter	ICD10CM
T63.002S	Toxic effect of unspecified snake venom, intentional self-harm, sequela	ICD10CM
T63.012	Toxic effect of rattlesnake venom, intentional self-harm	ICD10CM
T63.012A	Toxic effect of rattlesnake venom, intentional self-harm, initial encounter	ICD10CM
T63.012D	Toxic effect of rattlesnake venom, intentional self-harm, subsequent encounter	ICD10CM
T63.012S	Toxic effect of rattlesnake venom, intentional self-harm, sequela	ICD10CM
T63.022	Toxic effect of coral snake venom, intentional self-harm	ICD10CM
T63.022A	Toxic effect of coral snake venom, intentional self-harm, initial encounter	ICD10CM
T63.022D	Toxic effect of coral snake venom, intentional self-harm, subsequent encounter	ICD10CM
T63.022S	Toxic effect of coral snake venom, intentional self-harm, sequela	ICD10CM
T63.032	Toxic effect of taipan venom, intentional self-harm	ICD10CM
T63.032A	Toxic effect of taipan venom, intentional self-harm, initial encounter	ICD10CM
T63.032D	Toxic effect of taipan venom, intentional self-harm, subsequent encounter	ICD10CM
T63.032S	Toxic effect of taipan venom, intentional self-harm, sequela	ICD10CM
T63.042	Toxic effect of cobra venom, intentional self-harm	ICD10CM
T63.042A	Toxic effect of cobra venom, intentional self-harm, initial encounter	ICD10CM
T63.042D	Toxic effect of cobra venom, intentional self-harm, subsequent encounter	ICD10CM
T63.042S	Toxic effect of cobra venom, intentional self-harm, sequela	ICD10CM
T63.062	Toxic effect of venom of other North and South American snake, intentional self-harm	ICD10CM
T63.062A	Toxic effect of venom of other North and South American snake, intentional self-harm, initial encounter	ICD10CM

© 2021 Walsh CG et al. *JAMA Network Open.*

T63.062D	Toxic effect of venom of other North and South American snake, intentional self-harm, subsequent encounter	ICD10CM
T63.062S	Toxic effect of venom of other North and South American snake, intentional self-harm, sequela	ICD10CM
T63.072	Toxic effect of venom of other Australian snake, intentional self-harm	ICD10CM
T63.072A	Toxic effect of venom of other Australian snake, intentional self-harm, initial encounter	ICD10CM
T63.072D	Toxic effect of venom of other Australian snake, intentional self-harm, subsequent encounter	ICD10CM
T63.072S	Toxic effect of venom of other Australian snake, intentional self-harm, sequela	ICD10CM
T63.082	Toxic effect of venom of other African and Asian snake, intentional self-harm	ICD10CM
T63.082A	Toxic effect of venom of other African and Asian snake, intentional self-harm, initial encounter	ICD10CM
T63.082D	Toxic effect of venom of other African and Asian snake, intentional self-harm, subsequent encounter	ICD10CM
T63.082S	Toxic effect of venom of other African and Asian snake, intentional self-harm, sequela	ICD10CM
T63.092	Toxic effect of venom of other snake, intentional self-harm	ICD10CM
T63.092A	Toxic effect of venom of other snake, intentional self-harm, initial encounter	ICD10CM
T63.092D	Toxic effect of venom of other snake, intentional self-harm, subsequent encounter	ICD10CM
T63.092S	Toxic effect of venom of other snake, intentional self-harm, sequela	ICD10CM
T63.112	Toxic effect of venom of gila monster, intentional self-harm	ICD10CM
T63.112A	Toxic effect of venom of gila monster, intentional self-harm, initial encounter	ICD10CM
T63.112D	Toxic effect of venom of gila monster, intentional self-harm, subsequent encounter	ICD10CM
T63.112S	Toxic effect of venom of gila monster, intentional self-harm, sequela	ICD10CM
T63.122	Toxic effect of venom of other venomous lizard, intentional self-harm	ICD10CM
T63.122A	Toxic effect of venom of other venomous lizard, intentional self-harm, initial encounter	ICD10CM

© 2021 Walsh CG et al. *JAMA Network Open.*

**T63.122D**	Toxic effect of venom of other venomous lizard, intentional self-harm, subsequent encounter	ICD10CM
**T63.122S**	Toxic effect of venom of other venomous lizard, intentional self-harm, sequela	ICD10CM
**T63.192**	Toxic effect of venom of other reptiles, intentional self-harm	ICD10CM
**T63.192A**	Toxic effect of venom of other reptiles, intentional self-harm, initial encounter	ICD10CM
**T63.192D**	Toxic effect of venom of other reptiles, intentional self-harm, subsequent encounter	ICD10CM
**T63.192S**	Toxic effect of venom of other reptiles, intentional self-harm, sequela	ICD10CM
**T63.2X2**	Toxic effect of venom of scorpion, intentional self-harm	ICD10CM
**T63.2X2A**	Toxic effect of venom of scorpion, intentional self-harm, initial encounter	ICD10CM
**T63.2X2D**	Toxic effect of venom of scorpion, intentional self-harm, subsequent encounter	ICD10CM
**T63.2X2S**	Toxic effect of venom of scorpion, intentional self-harm, sequela	ICD10CM
**T63.302**	Toxic effect of unspecified spider venom, intentional self-harm	ICD10CM
**T63.302A**	Toxic effect of unspecified spider venom, intentional self-harm, initial encounter	ICD10CM
**T63.302D**	Toxic effect of unspecified spider venom, intentional self-harm, subsequent encounter	ICD10CM
**T63.302S**	Toxic effect of unspecified spider venom, intentional self-harm, sequela	ICD10CM
**T63.312**	Toxic effect of venom of black widow spider, intentional self-harm	ICD10CM
**T63.312A**	Toxic effect of venom of black widow spider, intentional self-harm, initial encounter	ICD10CM
**T63.312D**	Toxic effect of venom of black widow spider, intentional self-harm, subsequent encounter	ICD10CM
**T63.312S**	Toxic effect of venom of black widow spider, intentional self-harm, sequela	ICD10CM
**T63.322**	Toxic effect of venom of tarantula, intentional self-harm	ICD10CM
**T63.322A**	Toxic effect of venom of tarantula, intentional self-harm, initial encounter	ICD10CM
**T63.322D**	Toxic effect of venom of tarantula, intentional self-harm, subsequent encounter	ICD10CM

© 2021 Walsh CG et al. *JAMA Network Open.*

**T63.322S**	Toxic effect of venom of tarantula, intentional self-harm, sequela	ICD10CM
**T63.332**	Toxic effect of venom of brown recluse spider, intentional self-harm	ICD10CM
**T63.332A**	Toxic effect of venom of brown recluse spider, intentional self-harm, initial encounter	ICD10CM
**T63.332D**	Toxic effect of venom of brown recluse spider, intentional self-harm, subsequent encounter	ICD10CM
**T63.332S**	Toxic effect of venom of brown recluse spider, intentional self-harm, sequela	ICD10CM
**T63.392**	Toxic effect of venom of other spider, intentional self-harm	ICD10CM
**T63.392A**	Toxic effect of venom of other spider, intentional self-harm, initial encounter	ICD10CM
**T63.392D**	Toxic effect of venom of other spider, intentional self-harm, subsequent encounter	ICD10CM
**T63.392S**	Toxic effect of venom of other spider, intentional self-harm, sequela	ICD10CM
**T63.412**	Toxic effect of venom of centipedes and venomous millipedes, intentional self-harm	ICD10CM
**T63.412A**	Toxic effect of venom of centipedes and venomous millipedes, intentional self-harm, initial encounter	ICD10CM
**T63.412D**	Toxic effect of venom of centipedes and venomous millipedes, intentional self-harm, subsequent encounter	ICD10CM
**T63.412S**	Toxic effect of venom of centipedes and venomous millipedes, intentional self-harm, sequela	ICD10CM
**T63.422**	Toxic effect of venom of ants, intentional self-harm	ICD10CM
**T63.422A**	Toxic effect of venom of ants, intentional self-harm, initial encounter	ICD10CM
**T63.422D**	Toxic effect of venom of ants, intentional self-harm, subsequent encounter	ICD10CM
**T63.422S**	Toxic effect of venom of ants, intentional self-harm, sequela	ICD10CM
**T63.432**	Toxic effect of venom of caterpillars, intentional self-harm	ICD10CM
**T63.432A**	Toxic effect of venom of caterpillars, intentional self-harm, initial encounter	ICD10CM
**T63.432D**	Toxic effect of venom of caterpillars, intentional self-harm, subsequent encounter	ICD10CM
**T63.432S**	Toxic effect of venom of caterpillars, intentional self-harm, sequela	ICD10CM

T63.442	Toxic effect of venom of bees, intentional self-harm	ICD10CM
T63.442A	Toxic effect of venom of bees, intentional self-harm, initial encounter	ICD10CM
T63.442D	Toxic effect of venom of bees, intentional self-harm, subsequent encounter	ICD10CM
T63.442S	Toxic effect of venom of bees, intentional self-harm, sequela	ICD10CM
T63.452	Toxic effect of venom of hornets, intentional self-harm	ICD10CM
T63.452A	Toxic effect of venom of hornets, intentional self-harm, initial encounter	ICD10CM
T63.452D	Toxic effect of venom of hornets, intentional self-harm, subsequent encounter	ICD10CM
T63.452S	Toxic effect of venom of hornets, intentional self-harm, sequela	ICD10CM
T63.462	Toxic effect of venom of wasps, intentional self-harm	ICD10CM
T63.462A	Toxic effect of venom of wasps, intentional self-harm, initial encounter	ICD10CM
T63.462D	Toxic effect of venom of wasps, intentional self-harm, subsequent encounter	ICD10CM
T63.462S	Toxic effect of venom of wasps, intentional self-harm, sequela	ICD10CM
T63.482	Toxic effect of venom of other arthropod, intentional self-harm	ICD10CM
T63.482A	Toxic effect of venom of other arthropod, intentional self-harm, initial encounter	ICD10CM
T63.482D	Toxic effect of venom of other arthropod, intentional self-harm, subsequent encounter	ICD10CM
T63.482S	Toxic effect of venom of other arthropod, intentional self-harm, sequela	ICD10CM
T63.512	Toxic effect of contact with stingray, intentional self-harm	ICD10CM
T63.512A	Toxic effect of contact with stingray, intentional self-harm, initial encounter	ICD10CM
T63.512D	Toxic effect of contact with stingray, intentional self-harm, subsequent encounter	ICD10CM
T63.512S	Toxic effect of contact with stingray, intentional self-harm, sequela	ICD10CM
T63.592	Toxic effect of contact with other venomous fish, intentional self-harm	ICD10CM
T63.592A	Toxic effect of contact with other venomous fish, intentional self-harm, initial encounter	ICD10CM

© 2021 Walsh CG et al. *JAMA Network Open.*

**T63.592D**	Toxic effect of contact with other venomous fish, intentional self-harm, subsequent encounter	ICD10CM
**T63.592S**	Toxic effect of contact with other venomous fish, intentional self-harm, sequela	ICD10CM
**T63.612**	Toxic effect of contact with Portugese Man-o-war, intentional self-harm	ICD10CM
**T63.612A**	Toxic effect of contact with Portugese Man-o-war, intentional self-harm, initial encounter	ICD10CM
**T63.612D**	Toxic effect of contact with Portugese Man-o-war, intentional self-harm, subsequent encounter	ICD10CM
**T63.612S**	Toxic effect of contact with Portugese Man-o-war, intentional self-harm, sequela	ICD10CM
**T63.622**	Toxic effect of contact with other jellyfish, intentional self-harm	ICD10CM
**T63.622A**	Toxic effect of contact with other jellyfish, intentional self-harm, initial encounter	ICD10CM
**T63.622D**	Toxic effect of contact with other jellyfish, intentional self-harm, subsequent encounter	ICD10CM
**T63.622S**	Toxic effect of contact with other jellyfish, intentional self-harm, sequela	ICD10CM
**T63.632**	Toxic effect of contact with sea anemone, intentional self-harm	ICD10CM
**T63.632A**	Toxic effect of contact with sea anemone, intentional self-harm, initial encounter	ICD10CM
**T63.632D**	Toxic effect of contact with sea anemone, intentional self-harm, subsequent encounter	ICD10CM
**T63.632S**	Toxic effect of contact with sea anemone, intentional self-harm, sequela	ICD10CM
**T63.692**	Toxic effect of contact with other venomous marine animals, intentional self-harm	ICD10CM
**T63.692A**	Toxic effect of contact with other venomous marine animals, intentional self-harm, initial encounter	ICD10CM
**T63.692D**	Toxic effect of contact with other venomous marine animals, intentional self-harm, subsequent encounter	ICD10CM
**T63.692S**	Toxic effect of contact with other venomous marine animals, intentional self-harm, sequela	ICD10CM
**T63.712**	Toxic effect of contact with venomous marine plant, intentional self-harm	ICD10CM
**T63.712A**	Toxic effect of contact with venomous marine plant, intentional self-harm, initial encounter	ICD10CM

© 2021 Walsh CG et al. *JAMA Network Open.*

T63.712D	Toxic effect of contact with venomous marine plant, intentional self-harm, subsequent encounter	ICD10CM
T63.712S	Toxic effect of contact with venomous marine plant, intentional self-harm, sequela	ICD10CM
T63.792	Toxic effect of contact with other venomous plant, intentional self-harm	ICD10CM
T63.792A	Toxic effect of contact with other venomous plant, intentional self-harm, initial encounter	ICD10CM
T63.792D	Toxic effect of contact with other venomous plant, intentional self-harm, subsequent encounter	ICD10CM
T63.792S	Toxic effect of contact with other venomous plant, intentional self-harm, sequela	ICD10CM
T63.812	Toxic effect of contact with venomous frog, intentional self-harm	ICD10CM
T63.812A	Toxic effect of contact with venomous frog, intentional self-harm, initial encounter	ICD10CM
T63.812D	Toxic effect of contact with venomous frog, intentional self-harm, subsequent encounter	ICD10CM
T63.812S	Toxic effect of contact with venomous frog, intentional self-harm, sequela	ICD10CM
T63.822	Toxic effect of contact with venomous toad, intentional self-harm	ICD10CM
T63.822A	Toxic effect of contact with venomous toad, intentional self-harm, initial encounter	ICD10CM
T63.822D	Toxic effect of contact with venomous toad, intentional self-harm, subsequent encounter	ICD10CM
T63.822S	Toxic effect of contact with venomous toad, intentional self-harm, sequela	ICD10CM
T63.832	Toxic effect of contact with other venomous amphibian, intentional self-harm	ICD10CM
T63.832A	Toxic effect of contact with other venomous amphibian, intentional self-harm, initial encounter	ICD10CM
T63.832D	Toxic effect of contact with other venomous amphibian, intentional self-harm, subsequent encounter	ICD10CM
T63.832S	Toxic effect of contact with other venomous amphibian, intentional self-harm, sequela	ICD10CM
T63.892	Toxic effect of contact with other venomous animals, intentional self-harm	ICD10CM
T63.892A	Toxic effect of contact with other venomous animals, intentional self-harm, initial encounter	ICD10CM

© 2021 Walsh CG et al. *JAMA Network Open.*

**T63.892D**	Toxic effect of contact with other venomous animals, intentional self-harm, subsequent encounter	ICD10CM
**T63.892S**	Toxic effect of contact with other venomous animals, intentional self-harm, sequela	ICD10CM
**T63.92**	Toxic effect of contact with unspecified venomous animal, intentional self-harm	ICD10CM
**T63.92XA**	Toxic effect of contact with unspecified venomous animal, intentional self-harm, initial encounter	ICD10CM
**T63.92XD**	Toxic effect of contact with unspecified venomous animal, intentional self-harm, subsequent encounter	ICD10CM
**T63.92XS**	Toxic effect of contact with unspecified venomous animal, intentional self-harm, sequela	ICD10CM
**T64.02**	Toxic effect of aflatoxin, intentional self-harm	ICD10CM
**T64.02XA**	Toxic effect of aflatoxin, intentional self-harm, initial encounter	ICD10CM
**T64.02XD**	Toxic effect of aflatoxin, intentional self-harm, subsequent encounter	ICD10CM
**T64.02XS**	Toxic effect of aflatoxin, intentional self-harm, sequela	ICD10CM
**T64.82**	Toxic effect of other mycotoxin food contaminants, intentional self-harm	ICD10CM
**T64.82XA**	Toxic effect of other mycotoxin food contaminants, intentional self-harm, initial encounter	ICD10CM
**T64.82XD**	Toxic effect of other mycotoxin food contaminants, intentional self-harm, subsequent encounter	ICD10CM
**T64.82XS**	Toxic effect of other mycotoxin food contaminants, intentional self-harm, sequela	ICD10CM
**T65.0X2**	Toxic effect of cyanides, intentional self-harm	ICD10CM
**T65.0X2A**	Toxic effect of cyanides, intentional self-harm, initial encounter	ICD10CM
**T65.0X2D**	Toxic effect of cyanides, intentional self-harm, subsequent encounter	ICD10CM
**T65.0X2S**	Toxic effect of cyanides, intentional self-harm, sequela	ICD10CM
**T65.1X2**	Toxic effect of strychnine and its salts, intentional self-harm	ICD10CM
**T65.1X2A**	Toxic effect of strychnine and its salts, intentional self-harm, initial encounter	ICD10CM

© 2021 Walsh CG et al. *JAMA Network Open.*

**T65.1X2D**	Toxic effect of strychnine and its salts, intentional self-harm, subsequent encounter	ICD10CM
**T65.1X2S**	Toxic effect of strychnine and its salts, intentional self-harm, sequela	ICD10CM
**T65.212**	Toxic effect of chewing tobacco, intentional self-harm	ICD10CM
**T65.212A**	Toxic effect of chewing tobacco, intentional self-harm, initial encounter	ICD10CM
**T65.212D**	Toxic effect of chewing tobacco, intentional self-harm, subsequent encounter	ICD10CM
**T65.212S**	Toxic effect of chewing tobacco, intentional self-harm, sequela	ICD10CM
**T65.222**	Toxic effect of tobacco cigarettes, intentional self-harm	ICD10CM
**T65.222A**	Toxic effect of tobacco cigarettes, intentional self-harm, initial encounter	ICD10CM
**T65.222D**	Toxic effect of tobacco cigarettes, intentional self-harm, subsequent encounter	ICD10CM
**T65.222S**	Toxic effect of tobacco cigarettes, intentional self-harm, sequela	ICD10CM
**T65.292**	Toxic effect of other tobacco and nicotine, intentional self-harm	ICD10CM
**T65.292A**	Toxic effect of other tobacco and nicotine, intentional self-harm, initial encounter	ICD10CM
**T65.292D**	Toxic effect of other tobacco and nicotine, intentional self-harm, subsequent encounter	ICD10CM
**T65.292S**	Toxic effect of other tobacco and nicotine, intentional self-harm, sequela	ICD10CM
**T65.3X2**	Toxic effect of nitroderivatives and aminoderivatives of benzene and its homologues, intentional self-harm	ICD10CM
**T65.3X2A**	Toxic effect of nitroderivatives and aminoderivatives of benzene and its homologues, intentional self-harm, initial encounter	ICD10CM
**T65.3X2D**	Toxic effect of nitroderivatives and aminoderivatives of benzene and its homologues, intentional self-harm, subsequent encounter	ICD10CM
**T65.3X2S**	Toxic effect of nitroderivatives and aminoderivatives of benzene and its homologues, intentional self-harm, sequela	ICD10CM
**T65.4X2**	Toxic effect of carbon disulfide, intentional self-harm	ICD10CM
**T65.4X2A**	Toxic effect of carbon disulfide, intentional self-harm, initial encounter	ICD10CM
**T65.4X2D**	Toxic effect of carbon disulfide, intentional self-harm, subsequent encounter	ICD10CM

© 2021 Walsh CG et al. *JAMA Network Open.*

**T65.4X2S**	Toxic effect of carbon disulfide, intentional self-harm, sequela	ICD10CM
**T65.5X2**	Toxic effect of nitroglycerin and other nitric acids and esters, intentional self-harm	ICD10CM
**T65.5X2A**	Toxic effect of nitroglycerin and other nitric acids and esters, intentional self-harm, initial encounter	ICD10CM
**T65.5X2D**	Toxic effect of nitroglycerin and other nitric acids and esters, intentional self-harm, subsequent encounter	ICD10CM
**T65.5X2S**	Toxic effect of nitroglycerin and other nitric acids and esters, intentional self-harm, sequela	ICD10CM
**T65.6X2**	Toxic effect of paints and dyes, not elsewhere classified, intentional self-harm	ICD10CM
**T65.6X2A**	Toxic effect of paints and dyes, not elsewhere classified, intentional self-harm, initial encounter	ICD10CM
**T65.6X2D**	Toxic effect of paints and dyes, not elsewhere classified, intentional self-harm, subsequent encounter	ICD10CM
**T65.6X2S**	Toxic effect of paints and dyes, not elsewhere classified, intentional self-harm, sequela	ICD10CM
**T65.812**	Toxic effect of latex, intentional self-harm	ICD10CM
**T65.812A**	Toxic effect of latex, intentional self-harm, initial encounter	ICD10CM
**T65.812D**	Toxic effect of latex, intentional self-harm, subsequent encounter	ICD10CM
**T65.812S**	Toxic effect of latex, intentional self-harm, sequela	ICD10CM
**T65.822**	Toxic effect of harmful algae and algae toxins, intentional self-harm	ICD10CM
**T65.822A**	Toxic effect of harmful algae and algae toxins, intentional self-harm, initial encounter	ICD10CM
**T65.822D**	Toxic effect of harmful algae and algae toxins, intentional self-harm, subsequent encounter	ICD10CM
**T65.822S**	Toxic effect of harmful algae and algae toxins, intentional self-harm, sequela	ICD10CM
**T65.832**	Toxic effect of fiberglass, intentional self-harm	ICD10CM
**T65.832A**	Toxic effect of fiberglass, intentional self-harm, initial encounter	ICD10CM
**T65.832D**	Toxic effect of fiberglass, intentional self-harm, subsequent encounter	ICD10CM

© 2021 Walsh CG et al. *JAMA Network Open.*

T65.832S	Toxic effect of fiberglass, intentional self-harm, sequela	ICD10CM
T65.892	Toxic effect of other specified substances, intentional self-harm	ICD10CM
T65.892A	Toxic effect of other specified substances, intentional self-harm, initial encounter	ICD10CM
T65.892D	Toxic effect of other specified substances, intentional self-harm, subsequent encounter	ICD10CM
T65.892S	Toxic effect of other specified substances, intentional self-harm, sequela	ICD10CM
T65.92	Toxic effect of unspecified substance, intentional self-harm	ICD10CM
T65.92XA	Toxic effect of unspecified substance, intentional self-harm, initial encounter	ICD10CM
T65.92XD	Toxic effect of unspecified substance, intentional self-harm, subsequent encounter	ICD10CM
T65.92XS	Toxic effect of unspecified substance, intentional self-harm, sequela	ICD10CM
T71.112	Asphyxiation due to smothering under pillow, intentional self-harm	ICD10CM
T71.112A	Asphyxiation due to smothering under pillow, intentional self-harm, initial encounter	ICD10CM
T71.112D	Asphyxiation due to smothering under pillow, intentional self-harm, subsequent encounter	ICD10CM
T71.112S	Asphyxiation due to smothering under pillow, intentional self-harm, sequela	ICD10CM
T71.122	Asphyxiation due to plastic bag, intentional self-harm	ICD10CM
T71.122A	Asphyxiation due to plastic bag, intentional self-harm, initial encounter	ICD10CM
T71.122D	Asphyxiation due to plastic bag, intentional self-harm, subsequent encounter	ICD10CM
T71.122S	Asphyxiation due to plastic bag, intentional self-harm, sequela	ICD10CM
T71.132	Asphyxiation due to being trapped in bed linens, intentional self-harm	ICD10CM
T71.132A	Asphyxiation due to being trapped in bed linens, intentional self-harm, initial encounter	ICD10CM
T71.132D	Asphyxiation due to being trapped in bed linens, intentional self-harm, subsequent encounter	ICD10CM
T71.132S	Asphyxiation due to being trapped in bed linens, intentional self-harm, sequela	ICD10CM

© 2021 Walsh CG et al. *JAMA Network Open.*

T71.152	Asphyxiation due to smothering in furniture, intentional self-harm	ICD10CM
T71.152A	Asphyxiation due to smothering in furniture, intentional self-harm, initial encounter	ICD10CM
T71.152D	Asphyxiation due to smothering in furniture, intentional self-harm, subsequent encounter	ICD10CM
T71.152S	Asphyxiation due to smothering in furniture, intentional self-harm, sequela	ICD10CM
T71.162	Asphyxiation due to hanging, intentional self-harm	ICD10CM
T71.162A	Asphyxiation due to hanging, intentional self-harm, initial encounter	ICD10CM
T71.162D	Asphyxiation due to hanging, intentional self-harm, subsequent encounter	ICD10CM
T71.162S	Asphyxiation due to hanging, intentional self-harm, sequela	ICD10CM
T71.192	Asphyxiation due to mechanical threat to breathing due to other causes, intentional self-harm	ICD10CM
T71.192A	Asphyxiation due to mechanical threat to breathing due to other causes, intentional self-harm, initial encounter	ICD10CM
T71.192D	Asphyxiation due to mechanical threat to breathing due to other causes, intentional self-harm, subsequent encounter	ICD10CM
T71.192S	Asphyxiation due to mechanical threat to breathing due to other causes, intentional self-harm, sequela	ICD10CM
T71.222	Asphyxiation due to being trapped in a car trunk, intentional self-harm	ICD10CM
T71.222A	Asphyxiation due to being trapped in a car trunk, intentional self-harm, initial encounter	ICD10CM
T71.222D	Asphyxiation due to being trapped in a car trunk, intentional self-harm, subsequent encounter	ICD10CM
T71.222S	Asphyxiation due to being trapped in a car trunk, intentional self-harm, sequela	ICD10CM
T71.232	Asphyxiation due to being trapped in a (discarded) refrigerator, intentional self-harm	ICD10CM
T71.232A	Asphyxiation due to being trapped in a (discarded) refrigerator, intentional self-harm, initial encounter	ICD10CM
T71.232D	Asphyxiation due to being trapped in a (discarded) refrigerator, intentional self-harm, subsequent encounter	ICD10CM

© 2021 Walsh CG et al. *JAMA Network Open.*

T71.232S	Asphyxiation due to being trapped in a (discarded) refrigerator, intentional self-harm, sequela	ICD10CM
X71	Intentional self-harm by drowning and submersion	ICD10CM
X71.0	Intentional self-harm by drowning and submersion while in bathtub	ICD10CM
X71.0XXA	Intentional self-harm by drowning and submersion while in bathtub, initial encounter	ICD10CM
X71.0XXD	Intentional self-harm by drowning and submersion while in bathtub, subsequent encounter	ICD10CM
X71.0XXS	Intentional self-harm by drowning and submersion while in bathtub, sequela	ICD10CM
X71.1	Intentional self-harm by drowning and submersion while in swimming pool	ICD10CM
X71.1XXA	Intentional self-harm by drowning and submersion while in swimming pool, initial encounter	ICD10CM
X71.1XXD	Intentional self-harm by drowning and submersion while in swimming pool, subsequent encounter	ICD10CM
X71.1XXS	Intentional self-harm by drowning and submersion while in swimming pool, sequela	ICD10CM
X71.2	Intentional self-harm by drowning and submersion after jump into swimming pool	ICD10CM
X71.2XXA	Intentional self-harm by drowning and submersion after jump into swimming pool, initial encounter	ICD10CM
X71.2XXD	Intentional self-harm by drowning and submersion after jump into swimming pool, subsequent encounter	ICD10CM
X71.2XXS	Intentional self-harm by drowning and submersion after jump into swimming pool, sequela	ICD10CM
X71.3	Intentional self-harm by drowning and submersion in natural water	ICD10CM
X71.3XXA	Intentional self-harm by drowning and submersion in natural water, initial encounter	ICD10CM
X71.3XXD	Intentional self-harm by drowning and submersion in natural water, subsequent encounter	ICD10CM
X71.3XXS	Intentional self-harm by drowning and submersion in natural water, sequela	ICD10CM
X71.8	Other intentional self-harm by drowning and submersion	ICD10CM

© 2021 Walsh CG et al. *JAMA Network Open.*

X71.8XXA	Other intentional self-harm by drowning and submersion, initial encounter	ICD10CM
X71.8XXD	Other intentional self-harm by drowning and submersion, subsequent encounter	ICD10CM
X71.8XXS	Other intentional self-harm by drowning and submersion, sequela	ICD10CM
X71.9	Intentional self-harm by drowning and submersion, unspecified	ICD10CM
X71.9XXA	Intentional self-harm by drowning and submersion, unspecified, initial encounter	ICD10CM
X71.9XXD	Intentional self-harm by drowning and submersion, unspecified, subsequent encounter	ICD10CM
X71.9XXS	Intentional self-harm by drowning and submersion, unspecified, sequela	ICD10CM
X72	Intentional self-harm by handgun discharge	ICD10CM
X72.XXXA	Intentional self-harm by handgun discharge, initial encounter	ICD10CM
X72.XXXD	Intentional self-harm by handgun discharge, subsequent encounter	ICD10CM
X72.XXXS	Intentional self-harm by handgun discharge, sequela	ICD10CM
X73	Intentional self-harm by rifle, shotgun and larger firearm discharge	ICD10CM
X73.0	Intentional self-harm by shotgun discharge	ICD10CM
X73.0XXA	Intentional self-harm by shotgun discharge, initial encounter	ICD10CM
X73.0XXD	Intentional self-harm by shotgun discharge, subsequent encounter	ICD10CM
X73.0XXS	Intentional self-harm by shotgun discharge, sequela	ICD10CM
X73.1	Intentional self-harm by hunting rifle discharge	ICD10CM
X73.1XXA	Intentional self-harm by hunting rifle discharge, initial encounter	ICD10CM
X73.1XXD	Intentional self-harm by hunting rifle discharge, subsequent encounter	ICD10CM
X73.1XXS	Intentional self-harm by hunting rifle discharge, sequela	ICD10CM
X73.2	Intentional self-harm by machine gun discharge	ICD10CM
X73.2XXA	Intentional self-harm by machine gun discharge, initial encounter	ICD10CM

© 2021 Walsh CG et al. *JAMA Network Open.*

X73.2XXD	Intentional self-harm by machine gun discharge, subsequent encounter	ICD10CM
X73.2XXS	Intentional self-harm by machine gun discharge, sequela	ICD10CM
X73.8	Intentional self-harm by other larger firearm discharge	ICD10CM
X73.8XXA	Intentional self-harm by other larger firearm discharge, initial encounter	ICD10CM
X73.8XXD	Intentional self-harm by other larger firearm discharge, subsequent encounter	ICD10CM
X73.8XXS	Intentional self-harm by other larger firearm discharge, sequela	ICD10CM
X73.9	Intentional self-harm by unspecified larger firearm discharge	ICD10CM
X73.9XXA	Intentional self-harm by unspecified larger firearm discharge, initial encounter	ICD10CM
X73.9XXD	Intentional self-harm by unspecified larger firearm discharge, subsequent encounter	ICD10CM
X73.9XXS	Intentional self-harm by unspecified larger firearm discharge, sequela	ICD10CM
X74	Intentional self-harm by other and unspecified firearm and gun discharge	ICD10CM
X74.0	Intentional self-harm by gas, air or spring-operated guns	ICD10CM
X74.01	Intentional self-harm by airgun	ICD10CM
X74.01XA	Intentional self-harm by airgun, initial encounter	ICD10CM
X74.01XD	Intentional self-harm by airgun, subsequent encounter	ICD10CM
X74.01XS	Intentional self-harm by airgun, sequela	ICD10CM
X74.02	Intentional self-harm by paintball gun	ICD10CM
X74.02XA	Intentional self-harm by paintball gun, initial encounter	ICD10CM
X74.02XD	Intentional self-harm by paintball gun, subsequent encounter	ICD10CM
X74.02XS	Intentional self-harm by paintball gun, sequela	ICD10CM
X74.09	Intentional self-harm by other gas, air or spring-operated gun	ICD10CM
X74.09XA	Intentional self-harm by other gas, air or spring-operated gun, initial encounter	ICD10CM

© 2021 Walsh CG et al. *JAMA Network Open.*

X74.09XD	Intentional self-harm by other gas, air or spring-operated gun, subsequent encounter	ICD10CM
X74.09XS	Intentional self-harm by other gas, air or spring-operated gun, sequela	ICD10CM
X74.8	Intentional self-harm by other firearm discharge	ICD10CM
X74.8XXA	Intentional self-harm by other firearm discharge, initial encounter	ICD10CM
X74.8XXD	Intentional self-harm by other firearm discharge, subsequent encounter	ICD10CM
X74.8XXS	Intentional self-harm by other firearm discharge, sequela	ICD10CM
X74.9	Intentional self-harm by unspecified firearm discharge	ICD10CM
X74.9XXA	Intentional self-harm by unspecified firearm discharge, initial encounter	ICD10CM
X74.9XXD	Intentional self-harm by unspecified firearm discharge, subsequent encounter	ICD10CM
X74.9XXS	Intentional self-harm by unspecified firearm discharge, sequela	ICD10CM
X75	Intentional self-harm by explosive material	ICD10CM
X75.XXXA	Intentional self-harm by explosive material, initial encounter	ICD10CM
X75.XXXD	Intentional self-harm by explosive material, subsequent encounter	ICD10CM
X75.XXXS	Intentional self-harm by explosive material, sequela	ICD10CM
X76	Intentional self-harm by smoke, fire and flames	ICD10CM
X76.XXXA	Intentional self-harm by smoke, fire and flames, initial encounter	ICD10CM
X76.XXXD	Intentional self-harm by smoke, fire and flames, subsequent encounter	ICD10CM
X76.XXXS	Intentional self-harm by smoke, fire and flames, sequela	ICD10CM
X77	Intentional self-harm by steam, hot vapors and hot objects	ICD10CM
X77.0	Intentional self-harm by steam or hot vapors	ICD10CM
X77.0XXA	Intentional self-harm by steam or hot vapors, initial encounter	ICD10CM
X77.0XXD	Intentional self-harm by steam or hot vapors, subsequent encounter	ICD10CM

© 2021 Walsh CG et al. *JAMA Network Open.*

X77.0XXS	Intentional self-harm by steam or hot vapors, sequela	ICD10CM
X77.1	Intentional self-harm by hot tap water	ICD10CM
X77.1XXA	Intentional self-harm by hot tap water, initial encounter	ICD10CM
X77.1XXD	Intentional self-harm by hot tap water, subsequent encounter	ICD10CM
X77.1XXS	Intentional self-harm by hot tap water, sequela	ICD10CM
X77.2	Intentional self-harm by other hot fluids	ICD10CM
X77.2XXA	Intentional self-harm by other hot fluids, initial encounter	ICD10CM
X77.2XXD	Intentional self-harm by other hot fluids, subsequent encounter	ICD10CM
X77.2XXS	Intentional self-harm by other hot fluids, sequela	ICD10CM
X77.3	Intentional self-harm by hot household appliances	ICD10CM
X77.3XXA	Intentional self-harm by hot household appliances, initial encounter	ICD10CM
X77.3XXD	Intentional self-harm by hot household appliances, subsequent encounter	ICD10CM
X77.3XXS	Intentional self-harm by hot household appliances, sequela	ICD10CM
X77.8	Intentional self-harm by other hot objects	ICD10CM
X77.8XXA	Intentional self-harm by other hot objects, initial encounter	ICD10CM
X77.8XXD	Intentional self-harm by other hot objects, subsequent encounter	ICD10CM
X77.8XXS	Intentional self-harm by other hot objects, sequela	ICD10CM
X77.9	Intentional self-harm by unspecified hot objects	ICD10CM
X77.9XXA	Intentional self-harm by unspecified hot objects, initial encounter	ICD10CM
X77.9XXD	Intentional self-harm by unspecified hot objects, subsequent encounter	ICD10CM
X77.9XXS	Intentional self-harm by unspecified hot objects, sequela	ICD10CM
X78	Intentional self-harm by sharp object	ICD10CM

© 2021 Walsh CG et al. *JAMA Network Open.*

X78.0	Intentional self-harm by sharp glass	ICD10CM
X78.0XXA	Intentional self-harm by sharp glass, initial encounter	ICD10CM
X78.0XXD	Intentional self-harm by sharp glass, subsequent encounter	ICD10CM
X78.0XXS	Intentional self-harm by sharp glass, sequela	ICD10CM
X78.1	Intentional self-harm by knife	ICD10CM
X78.1XXA	Intentional self-harm by knife, initial encounter	ICD10CM
X78.1XXD	Intentional self-harm by knife, subsequent encounter	ICD10CM
X78.1XXS	Intentional self-harm by knife, sequela	ICD10CM
X78.2	Intentional self-harm by sword or dagger	ICD10CM
X78.2XXA	Intentional self-harm by sword or dagger, initial encounter	ICD10CM
X78.2XXD	Intentional self-harm by sword or dagger, subsequent encounter	ICD10CM
X78.2XXS	Intentional self-harm by sword or dagger, sequela	ICD10CM
X78.8	Intentional self-harm by other sharp object	ICD10CM
X78.8XXA	Intentional self-harm by other sharp object, initial encounter	ICD10CM
X78.8XXD	Intentional self-harm by other sharp object, subsequent encounter	ICD10CM
X78.8XXS	Intentional self-harm by other sharp object, sequela	ICD10CM
X78.9	Intentional self-harm by unspecified sharp object	ICD10CM
X78.9XXA	Intentional self-harm by unspecified sharp object, initial encounter	ICD10CM
X78.9XXD	Intentional self-harm by unspecified sharp object, subsequent encounter	ICD10CM
X78.9XXS	Intentional self-harm by unspecified sharp object, sequela	ICD10CM
X79	Intentional self-harm by blunt object	ICD10CM
X79.XXXA	Intentional self-harm by blunt object, initial encounter	ICD10CM

© 2021 Walsh CG et al. *JAMA Network Open.*

X79.XXXD	Intentional self-harm by blunt object, subsequent encounter	ICD10CM
X79.XXXS	Intentional self-harm by blunt object, sequela	ICD10CM
X80	Intentional self-harm by jumping from a high place	ICD10CM
X80.XXXA	Intentional self-harm by jumping from a high place, initial encounter	ICD10CM
X80.XXXD	Intentional self-harm by jumping from a high place, subsequent encounter	ICD10CM
X80.XXXS	Intentional self-harm by jumping from a high place, sequela	ICD10CM
X81	Intentional self-harm by jumping or lying in front of moving object	ICD10CM
X81.0	Intentional self-harm by jumping or lying in front of motor vehicle	ICD10CM
X81.0XXA	Intentional self-harm by jumping or lying in front of motor vehicle, initial encounter	ICD10CM
X81.0XXD	Intentional self-harm by jumping or lying in front of motor vehicle, subsequent encounter	ICD10CM
X81.0XXS	Intentional self-harm by jumping or lying in front of motor vehicle, sequela	ICD10CM
X81.1	Intentional self-harm by jumping or lying in front of (subway) train	ICD10CM
X81.1XXA	Intentional self-harm by jumping or lying in front of (subway) train, initial encounter	ICD10CM
X81.1XXD	Intentional self-harm by jumping or lying in front of (subway) train, subsequent encounter	ICD10CM
X81.1XXS	Intentional self-harm by jumping or lying in front of (subway) train, sequela	ICD10CM
X81.8	Intentional self-harm by jumping or lying in front of other moving object	ICD10CM
X81.8XXA	Intentional self-harm by jumping or lying in front of other moving object, initial encounter	ICD10CM
X81.8XXD	Intentional self-harm by jumping or lying in front of other moving object, subsequent encounter	ICD10CM
X81.8XXS	Intentional self-harm by jumping or lying in front of other moving object, sequela	ICD10CM
X82	Intentional self-harm by crashing of motor vehicle	ICD10CM

© 2021 Walsh CG et al. *JAMA Network Open.*

X82.0	Intentional collision of motor vehicle with other motor vehicle	ICD10CM
X82.0XXA	Intentional collision of motor vehicle with other motor vehicle, initial encounter	ICD10CM
X82.0XXD	Intentional collision of motor vehicle with other motor vehicle, subsequent encounter	ICD10CM
X82.0XXS	Intentional collision of motor vehicle with other motor vehicle, sequela	ICD10CM
X82.1	Intentional collision of motor vehicle with train	ICD10CM
X82.1XXA	Intentional collision of motor vehicle with train, initial encounter	ICD10CM
X82.1XXD	Intentional collision of motor vehicle with train, subsequent encounter	ICD10CM
X82.1XXS	Intentional collision of motor vehicle with train, sequela	ICD10CM
X82.2	Intentional collision of motor vehicle with tree	ICD10CM
X82.2XXA	Intentional collision of motor vehicle with tree, initial encounter	ICD10CM
X82.2XXD	Intentional collision of motor vehicle with tree, subsequent encounter	ICD10CM
X82.2XXS	Intentional collision of motor vehicle with tree, sequela	ICD10CM
X82.8	Other intentional self-harm by crashing of motor vehicle	ICD10CM
X82.8XXA	Other intentional self-harm by crashing of motor vehicle, initial encounter	ICD10CM
X82.8XXD	Other intentional self-harm by crashing of motor vehicle, subsequent encounter	ICD10CM
X82.8XXS	Other intentional self-harm by crashing of motor vehicle, sequela	ICD10CM
X83	Intentional self-harm by other specified means	ICD10CM
X83.0	Intentional self-harm by crashing of aircraft	ICD10CM
X83.0XXA	Intentional self-harm by crashing of aircraft, initial encounter	ICD10CM
X83.0XXD	Intentional self-harm by crashing of aircraft, subsequent encounter	ICD10CM
X83.0XXS	Intentional self-harm by crashing of aircraft, sequela	ICD10CM
X83.1	Intentional self-harm by electrocution	ICD10CM

© 2021 Walsh CG et al. *JAMA Network Open.*

X83.1XXA	Intentional self-harm by electrocution, initial encounter	ICD10CM
X83.1XXD	Intentional self-harm by electrocution, subsequent encounter	ICD10CM
X83.1XXS	Intentional self-harm by electrocution, sequela	ICD10CM
X83.2	Intentional self-harm by exposure to extremes of cold	ICD10CM
X83.2XXA	Intentional self-harm by exposure to extremes of cold, initial encounter	ICD10CM
X83.2XXD	Intentional self-harm by exposure to extremes of cold, subsequent encounter	ICD10CM
X83.2XXS	Intentional self-harm by exposure to extremes of cold, sequela	ICD10CM
X83.8	Intentional self-harm by other specified means	ICD10CM
X83.8XXA	Intentional self-harm by other specified means, initial encounter	ICD10CM
X83.8XXD	Intentional self-harm by other specified means, subsequent encounter	ICD10CM
X83.8XXS	Intentional self-harm by other specified means, sequela	ICD10CM
E950	Suicide and self-inflicted poisoning by solid or liquid substances	ICD9CM
E950.0	Suicide and self-inflicted poisoning by analgesics, antipyretics, and antirheumatics	ICD9CM
E950.1	Suicide and self-inflicted poisoning by barbiturates	ICD9CM
E950.2	Suicide and self-inflicted poisoning by other sedatives and hypnotics	ICD9CM
E950.3	Suicide and self-inflicted poisoning by tranquilizers and other psychotropic agents	ICD9CM
E950.4	Suicide and self-inflicted poisoning by other specified drugs and medicinal substances	ICD9CM
E950.5	Suicide and self-inflicted poisoning by unspecified drug or medicinal substance	ICD9CM
E950.6	Suicide and self-inflicted poisoning by agricultural and horticultural chemical and pharmaceutical preparations other than plant foods and fertilizers	ICD9CM
E950.7	Suicide and self-inflicted poisoning by corrosive and caustic substances	ICD9CM
E950.8	Suicide and self-inflicted poisoning by arsenic and its compounds	ICD9CM

© 2021 Walsh CG et al. *JAMA Network Open.*

**E950.9**	Suicide and self-inflicted poisoning by other and unspecified solid and liquid substances	ICD9CM
**E951**	Suicide and self-inflicted poisoning by gases in domestic use	ICD9CM
**E951.0**	Suicide and self-inflicted poisoning by gas distributed by pipeline	ICD9CM
**E951.1**	Suicide and self-inflicted poisoning by liquefied petroleum gas distributed in mobile containers	ICD9CM
**E951.8**	Suicide and self-inflicted poisoning by other utility gas	ICD9CM
**E952**	Suicide and self-inflicted poisoning by other gases and vapors	ICD9CM
**E952.0**	Suicide and self-inflicted poisoning by motor vehicle exhaust gas	ICD9CM
**E952.1**	Suicide and self-inflicted poisoning by other carbon monoxide	ICD9CM
**E952.8**	Suicide and self-inflicted poisoning by other specified gases and vapors	ICD9CM
**E952.9**	Suicide and self-inflicted poisoning by unspecified gases and vapors	ICD9CM
**E953**	Suicide and self-inflicted injury by hanging, strangulation, and suffocation	ICD9CM
**E953.0**	Suicide and self-inflicted injury by hanging	ICD9CM
**E953.1**	Suicide and self-inflicted injury by suffocation by plastic bag	ICD9CM
**E953.8**	Suicide and self-inflicted injury by other specified means	ICD9CM
**E953.9**	Suicide and self-inflicted injury by unspecified means	ICD9CM
**E954**	Suicide and self-inflicted injury by submersion [drowning]	ICD9CM
**E955**	Suicide and self-inflicted injury by firearms, air guns, and explosives	ICD9CM
**E955.0**	Suicide and self-inflicted injury by handgun	ICD9CM
**E955.1**	Suicide and self-inflicted injury by shotgun	ICD9CM
**E955.2**	Suicide and self-inflicted injury by hunting rifle	ICD9CM
**E955.3**	Suicide and self-inflicted injury by military firearms	ICD9CM
**E955.4**	Suicide and self-inflicted injury by other and unspecified firearm	ICD9CM

© 2021 Walsh CG et al. *JAMA Network Open.*

**E955.5**	Suicide and self-inflicted injury by explosives	ICD9CM
**E955.6**	Suicide and self-inflicted injury by air gun	ICD9CM
**E955.7**	Suicide and self-inflicted injury by paintball gun	ICD9CM
**E955.9**	Suicide and self-inflicted injury by firearms and explosives, unspecified	ICD9CM
**E956**	Suicide and self-inflicted injury by cutting and piercing instrument	ICD9CM
**E957**	Suicide and self-inflicted injuries by jumping from high place	ICD9CM
**E957.0**	Suicide and self-inflicted injuries by jumping from residential premises	ICD9CM
**E957.1**	Suicide and self-inflicted injuries by jumping from other man-made structures	ICD9CM
**E957.2**	Suicide and self-inflicted injuries by jumping from natural sites	ICD9CM
**E957.9**	Suicide and self-inflicted injuries by jumping from unspecified site	ICD9CM
**E958**	Suicide and self-inflicted injury by other and unspecified means	ICD9CM
**E958.0**	Suicide and self-inflicted injury by jumping or lying before moving object	ICD9CM
**E958.1**	Suicide and self-inflicted injury by burns, fire	ICD9CM
**E958.2**	Suicide and self-inflicted injury by scald	ICD9CM
**E958.3**	Suicide and self-inflicted injury by extremes of cold	ICD9CM
**E958.4**	Suicide and self-inflicted injury by electrocution	ICD9CM
**E958.5**	Suicide and self-inflicted injury by crashing of motor vehicle	ICD9CM
**E958.6**	Suicide and self-inflicted injury by crashing of aircraft	ICD9CM
**E958.7**	Suicide and self-inflicted injury by caustic substances, except poisoning	ICD9CM
**E958.8**	Suicide and self-inflicted injury by other specified means	ICD9CM
**E958.9**	Suicide and self-inflicted injury by unspecified means	ICD9CM
**E959**	Late effects of self-inflicted injury	ICD9CM

© 2021 Walsh CG et al. *JAMA Network Open.*

**Y10**	Poisoning by and exposure to nonopioid analgesics, antipyretics and antirheumatics, undetermined intent	ICD10
**Y11**	Poisoning by and exposure to antiepileptic, sedative-hypnotic, antiparkinsonism and psychotropic drugs, not elsewhere classified, undetermined intent	ICD10
**Y12**	Poisoning by and exposure to narcotics and psychodysleptics [hallucinogens], not elsewhere classified, undetermined intent	ICD10
**Y13**	Poisoning by and exposure to other drugs acting on the autonomic nervous system, undetermined intent	ICD10
**Y14**	Poisoning by and exposure to other and unspecified drugs, medicaments and biological substances, undetermined intent	ICD10
**Y15**	Poisoning by and exposure to alcohol, undetermined intent	ICD10
**Y16**	Poisoning by and exposure to organic solvents and halogenated hydrocarbons and their vapours, undetermined intent	ICD10
**Y17**	Poisoning by and exposure to other gases and vapours, undetermined intent	ICD10
**Y18**	Poisoning by and exposure to pesticides, undetermined intent	ICD10
**Y19**	Poisoning by and exposure to other and unspecified chemicals and noxious substances, undetermined intent	ICD10
**Y20**	Hanging, strangulation and suffocation, undetermined intent	ICD10
**Y21**	Drowning and submersion, undetermined intent	ICD10
**Y22**	Handgun discharge, undetermined intent	ICD10
**Y23**	Rifle, shotgun and larger firearm discharge, undetermined intent	ICD10
**Y24**	Other and unspecified firearm discharge, undetermined intent	ICD10
**Y25**	Contact with explosive material, undetermined intent	ICD10
**Y26**	Exposure to smoke, fire and flames, undetermined intent	ICD10
**Y27**	Contact with steam, hot vapours and hot objects, undetermined intent	ICD10
**Y28**	Contact with sharp object, undetermined intent	ICD10
**Y29**	Contact with blunt object, undetermined intent	ICD10

© 2021 Walsh CG et al. *JAMA Network Open.*

**Y30**	Falling, jumping or pushed from a high place, undetermined intent	ICD10
**Y31**	Falling, lying or running before or into moving object, undetermined intent	ICD10
**Y32**	Crashing of motor vehicle, undetermined intent	ICD10
**Y33**	Other specified events, undetermined intent	ICD10
**Y34**	Unspecified event, undetermined intent	ICD10
**Y87**	Sequelae of intentional self-harm, assault and events of undetermined intent	ICD10
**Y87.2**	Sequelae of events of undetermined intent	ICD10
**Y89.9**	Sequelae of unspecified external cause	ICD10
**Z91.5**	Personal history of self-harm	ICD10
**R45.851**	Suicidal ideation	ICD10CM
**T36.0X4**	Poisoning by penicillins, undetermined	ICD10CM
**T36.0X4A**	Poisoning by penicillins, undetermined, initial encounter	ICD10CM
**T36.0X4D**	Poisoning by penicillins, undetermined, subsequent encounter	ICD10CM
**T36.0X4S**	Poisoning by penicillins, undetermined, sequela	ICD10CM
**T36.1X4**	Poisoning by cephalosporins and other beta-lactam antibiotics, undetermined	ICD10CM
**T36.1X4A**	Poisoning by cephalosporins and other beta-lactam antibiotics, undetermined, initial encounter	ICD10CM
**T36.1X4D**	Poisoning by cephalosporins and other beta-lactam antibiotics, undetermined, subsequent encounter	ICD10CM
**T36.1X4S**	Poisoning by cephalosporins and other beta-lactam antibiotics, undetermined, sequela	ICD10CM
**T36.2X4**	Poisoning by chloramphenicol group, undetermined	ICD10CM
**T36.2X4A**	Poisoning by chloramphenicol group, undetermined, initial encounter	ICD10CM
**T36.2X4D**	Poisoning by chloramphenicol group, undetermined, subsequent encounter	ICD10CM

© 2021 Walsh CG et al. *JAMA Network Open.*

T36.2X4S	Poisoning by chloramphenicol group, undetermined, sequela	ICD10CM
T36.3X4	Poisoning by macrolides, undetermined	ICD10CM
T36.3X4A	Poisoning by macrolides, undetermined, initial encounter	ICD10CM
T36.3X4D	Poisoning by macrolides, undetermined, subsequent encounter	ICD10CM
T36.3X4S	Poisoning by macrolides, undetermined, sequela	ICD10CM
T36.4X4	Poisoning by tetracyclines, undetermined	ICD10CM
T36.4X4A	Poisoning by tetracyclines, undetermined, initial encounter	ICD10CM
T36.4X4D	Poisoning by tetracyclines, undetermined, subsequent encounter	ICD10CM
T36.4X4S	Poisoning by tetracyclines, undetermined, sequela	ICD10CM
T36.5X4	Poisoning by aminoglycosides, undetermined	ICD10CM
T36.5X4A	Poisoning by aminoglycosides, undetermined, initial encounter	ICD10CM
T36.5X4D	Poisoning by aminoglycosides, undetermined, subsequent encounter	ICD10CM
T36.5X4S	Poisoning by aminoglycosides, undetermined, sequela	ICD10CM
T36.6X4	Poisoning by rifampicins, undetermined	ICD10CM
T36.6X4A	Poisoning by rifampicins, undetermined, initial encounter	ICD10CM
T36.6X4D	Poisoning by rifampicins, undetermined, subsequent encounter	ICD10CM
T36.6X4S	Poisoning by rifampicins, undetermined, sequela	ICD10CM
T36.7X4	Poisoning by antifungal antibiotics, systemically used, undetermined	ICD10CM
T36.7X4A	Poisoning by antifungal antibiotics, systemically used, undetermined, initial encounter	ICD10CM
T36.7X4D	Poisoning by antifungal antibiotics, systemically used, undetermined, subsequent encounter	ICD10CM
T36.7X4S	Poisoning by antifungal antibiotics, systemically used, undetermined, sequela	ICD10CM
T36.8X4	Poisoning by other systemic antibiotics, undetermined	ICD10CM

© 2021 Walsh CG et al. *JAMA Network Open.*

T36.8X4A	Poisoning by other systemic antibiotics, undetermined, initial encounter	ICD10CM
T36.8X4D	Poisoning by other systemic antibiotics, undetermined, subsequent encounter	ICD10CM
T36.8X4S	Poisoning by other systemic antibiotics, undetermined, sequela	ICD10CM
T36.94	Poisoning by unspecified systemic antibiotic, undetermined	ICD10CM
T36.94XA	Poisoning by unspecified systemic antibiotic, undetermined, initial encounter	ICD10CM
T36.94XD	Poisoning by unspecified systemic antibiotic, undetermined, subsequent encounter	ICD10CM
T36.94XS	Poisoning by unspecified systemic antibiotic, undetermined, sequela	ICD10CM
T37.0X4	Poisoning by sulfonamides, undetermined	ICD10CM
T37.0X4A	Poisoning by sulfonamides, undetermined, initial encounter	ICD10CM
T37.0X4D	Poisoning by sulfonamides, undetermined, subsequent encounter	ICD10CM
T37.0X4S	Poisoning by sulfonamides, undetermined, sequela	ICD10CM
T37.1X4	Poisoning by antimycobacterial drugs, undetermined	ICD10CM
T37.1X4A	Poisoning by antimycobacterial drugs, undetermined, initial encounter	ICD10CM
T37.1X4D	Poisoning by antimycobacterial drugs, undetermined, subsequent encounter	ICD10CM
T37.1X4S	Poisoning by antimycobacterial drugs, undetermined, sequela	ICD10CM
T37.2X4	Poisoning by antimalarials and drugs acting on other blood protozoa, undetermined	ICD10CM
T37.2X4A	Poisoning by antimalarials and drugs acting on other blood protozoa, undetermined, initial encounter	ICD10CM
T37.2X4D	Poisoning by antimalarials and drugs acting on other blood protozoa, undetermined, subsequent encounter	ICD10CM
T37.2X4S	Poisoning by antimalarials and drugs acting on other blood protozoa, undetermined, sequela	ICD10CM
T37.3X4	Poisoning by other antiprotozoal drugs, undetermined	ICD10CM
T37.3X4A	Poisoning by other antiprotozoal drugs, undetermined, initial encounter	ICD10CM

© 2021 Walsh CG et al. *JAMA Network Open.*

T37.3X4D	Poisoning by other antiprotozoal drugs, undetermined, subsequent encounter	ICD10CM
T37.3X4S	Poisoning by other antiprotozoal drugs, undetermined, sequela	ICD10CM
T37.4X4	Poisoning by anthelminthics, undetermined	ICD10CM
T37.4X4A	Poisoning by anthelminthics, undetermined, initial encounter	ICD10CM
T37.4X4D	Poisoning by anthelminthics, undetermined, subsequent encounter	ICD10CM
T37.4X4S	Poisoning by anthelminthics, undetermined, sequela	ICD10CM
T37.5X4	Poisoning by antiviral drugs, undetermined	ICD10CM
T37.5X4A	Poisoning by antiviral drugs, undetermined, initial encounter	ICD10CM
T37.5X4D	Poisoning by antiviral drugs, undetermined, subsequent encounter	ICD10CM
T37.5X4S	Poisoning by antiviral drugs, undetermined, sequela	ICD10CM
T37.8X4	Poisoning by other specified systemic anti-infectives and antiparasitics, undetermined	ICD10CM
T37.8X4A	Poisoning by other specified systemic anti-infectives and antiparasitics, undetermined, initial encounter	ICD10CM
T37.8X4D	Poisoning by other specified systemic anti-infectives and antiparasitics, undetermined, subsequent encounter	ICD10CM
T37.8X4S	Poisoning by other specified systemic anti-infectives and antiparasitics, undetermined, sequela	ICD10CM
T37.94	Poisoning by unspecified systemic anti-infective and antiparasitics, undetermined	ICD10CM
T37.94XA	Poisoning by unspecified systemic anti-infective and antiparasitics, undetermined, initial encounter	ICD10CM
T37.94XD	Poisoning by unspecified systemic anti-infective and antiparasitics, undetermined, subsequent encounter	ICD10CM
T37.94XS	Poisoning by unspecified systemic anti-infective and antiparasitics, undetermined, sequela	ICD10CM
T38.0X4	Poisoning by glucocorticoids and synthetic analogues, undetermined	ICD10CM
T38.0X4A	Poisoning by glucocorticoids and synthetic analogues, undetermined, initial encounter	ICD10CM

© 2021 Walsh CG et al. *JAMA Network Open.*

T38.0X4D	Poisoning by glucocorticoids and synthetic analogues, undetermined, subsequent encounter	ICD10CM
T38.0X4S	Poisoning by glucocorticoids and synthetic analogues, undetermined, sequela	ICD10CM
T38.1X4	Poisoning by thyroid hormones and substitutes, undetermined	ICD10CM
T38.1X4A	Poisoning by thyroid hormones and substitutes, undetermined, initial encounter	ICD10CM
T38.1X4D	Poisoning by thyroid hormones and substitutes, undetermined, subsequent encounter	ICD10CM
T38.1X4S	Poisoning by thyroid hormones and substitutes, undetermined, sequela	ICD10CM
T38.2X4	Poisoning by antithyroid drugs, undetermined	ICD10CM
T38.2X4A	Poisoning by antithyroid drugs, undetermined, initial encounter	ICD10CM
T38.2X4D	Poisoning by antithyroid drugs, undetermined, subsequent encounter	ICD10CM
T38.2X4S	Poisoning by antithyroid drugs, undetermined, sequela	ICD10CM
T38.3X4	Poisoning by insulin and oral hypoglycemic [antidiabetic] drugs, undetermined	ICD10CM
T38.3X4A	Poisoning by insulin and oral hypoglycemic [antidiabetic] drugs, undetermined, initial encounter	ICD10CM
T38.3X4D	Poisoning by insulin and oral hypoglycemic [antidiabetic] drugs, undetermined, subsequent encounter	ICD10CM
T38.3X4S	Poisoning by insulin and oral hypoglycemic [antidiabetic] drugs, undetermined, sequela	ICD10CM
T38.4X4	Poisoning by oral contraceptives, undetermined	ICD10CM
T38.4X4A	Poisoning by oral contraceptives, undetermined, initial encounter	ICD10CM
T38.4X4D	Poisoning by oral contraceptives, undetermined, subsequent encounter	ICD10CM
T38.4X4S	Poisoning by oral contraceptives, undetermined, sequela	ICD10CM
T38.5X4	Poisoning by other estrogens and progestogens, undetermined	ICD10CM
T38.5X4A	Poisoning by other estrogens and progestogens, undetermined, initial encounter	ICD10CM

© 2021 Walsh CG et al. *JAMA Network Open.*

T38.5X4D	Poisoning by other estrogens and progestogens, undetermined, subsequent encounter	ICD10CM
T38.5X4S	Poisoning by other estrogens and progestogens, undetermined, sequela	ICD10CM
T38.6X4	Poisoning by antigonadotrophins, antiestrogens, antiandrogens, not elsewhere classified, undetermined	ICD10CM
T38.6X4A	Poisoning by antigonadotrophins, antiestrogens, antiandrogens, not elsewhere classified, undetermined, initial encounter	ICD10CM
T38.6X4D	Poisoning by antigonadotrophins, antiestrogens, antiandrogens, not elsewhere classified, undetermined, subsequent encounter	ICD10CM
T38.6X4S	Poisoning by antigonadotrophins, antiestrogens, antiandrogens, not elsewhere classified, undetermined, sequela	ICD10CM
T38.7X4	Poisoning by androgens and anabolic congeners, undetermined	ICD10CM
T38.7X4A	Poisoning by androgens and anabolic congeners, undetermined, initial encounter	ICD10CM
T38.7X4D	Poisoning by androgens and anabolic congeners, undetermined, subsequent encounter	ICD10CM
T38.7X4S	Poisoning by androgens and anabolic congeners, undetermined, sequela	ICD10CM
T38.804	Poisoning by unspecified hormones and synthetic substitutes, undetermined	ICD10CM
T38.804A	Poisoning by unspecified hormones and synthetic substitutes, undetermined, initial encounter	ICD10CM
T38.804D	Poisoning by unspecified hormones and synthetic substitutes, undetermined, subsequent encounter	ICD10CM
T38.804S	Poisoning by unspecified hormones and synthetic substitutes, undetermined, sequela	ICD10CM
T38.814	Poisoning by anterior pituitary [adenohypophyseal] hormones, undetermined	ICD10CM
T38.814A	Poisoning by anterior pituitary [adenohypophyseal] hormones, undetermined, initial encounter	ICD10CM
T38.814D	Poisoning by anterior pituitary [adenohypophyseal] hormones, undetermined, subsequent encounter	ICD10CM
T38.814S	Poisoning by anterior pituitary [adenohypophyseal] hormones, undetermined, sequela	ICD10CM
T38.894	Poisoning by other hormones and synthetic substitutes, undetermined	ICD10CM

© 2021 Walsh CG et al. *JAMA Network Open.*

T38.894A	Poisoning by other hormones and synthetic substitutes, undetermined, initial encounter	ICD10CM
T38.894D	Poisoning by other hormones and synthetic substitutes, undetermined, subsequent encounter	ICD10CM
T38.894S	Poisoning by other hormones and synthetic substitutes, undetermined, sequela	ICD10CM
T38.904	Poisoning by unspecified hormone antagonists, undetermined	ICD10CM
T38.904A	Poisoning by unspecified hormone antagonists, undetermined, initial encounter	ICD10CM
T38.904D	Poisoning by unspecified hormone antagonists, undetermined, subsequent encounter	ICD10CM
T38.904S	Poisoning by unspecified hormone antagonists, undetermined, sequela	ICD10CM
T38.994	Poisoning by other hormone antagonists, undetermined	ICD10CM
T38.994A	Poisoning by other hormone antagonists, undetermined, initial encounter	ICD10CM
T38.994D	Poisoning by other hormone antagonists, undetermined, subsequent encounter	ICD10CM
T38.994S	Poisoning by other hormone antagonists, undetermined, sequela	ICD10CM
T39.014	Poisoning by aspirin, undetermined	ICD10CM
T39.014A	Poisoning by aspirin, undetermined, initial encounter	ICD10CM
T39.014D	Poisoning by aspirin, undetermined, subsequent encounter	ICD10CM
T39.014S	Poisoning by aspirin, undetermined, sequela	ICD10CM
T39.094	Poisoning by salicylates, undetermined	ICD10CM
T39.094A	Poisoning by salicylates, undetermined, initial encounter	ICD10CM
T39.094D	Poisoning by salicylates, undetermined, subsequent encounter	ICD10CM
T39.094S	Poisoning by salicylates, undetermined, sequela	ICD10CM
T39.1X4	Poisoning by 4-Aminophenol derivatives, undetermined	ICD10CM
T39.1X4A	Poisoning by 4-Aminophenol derivatives, undetermined, initial encounter	ICD10CM

© 2021 Walsh CG et al. *JAMA Network Open.*

T39.1X4D	Poisoning by 4-Aminophenol derivatives, undetermined, subsequent encounter	ICD10CM
T39.1X4S	Poisoning by 4-Aminophenol derivatives, undetermined, sequela	ICD10CM
T39.2X4	Poisoning by pyrazolone derivatives, undetermined	ICD10CM
T39.2X4A	Poisoning by pyrazolone derivatives, undetermined, initial encounter	ICD10CM
T39.2X4D	Poisoning by pyrazolone derivatives, undetermined, subsequent encounter	ICD10CM
T39.2X4S	Poisoning by pyrazolone derivatives, undetermined, sequela	ICD10CM
T39.314	Poisoning by propionic acid derivatives, undetermined	ICD10CM
T39.314A	Poisoning by propionic acid derivatives, undetermined, initial encounter	ICD10CM
T39.314D	Poisoning by propionic acid derivatives, undetermined, subsequent encounter	ICD10CM
T39.314S	Poisoning by propionic acid derivatives, undetermined, sequela	ICD10CM
T39.394	Poisoning by other nonsteroidal anti-inflammatory drugs [NSAID], undetermined	ICD10CM
T39.394A	Poisoning by other nonsteroidal anti-inflammatory drugs [NSAID], undetermined, initial encounter	ICD10CM
T39.394D	Poisoning by other nonsteroidal anti-inflammatory drugs [NSAID], undetermined, subsequent encounter	ICD10CM
T39.394S	Poisoning by other nonsteroidal anti-inflammatory drugs [NSAID], undetermined, sequela	ICD10CM
T39.4X4	Poisoning by antirheumatics, not elsewhere classified, undetermined	ICD10CM
T39.4X4A	Poisoning by antirheumatics, not elsewhere classified, undetermined, initial encounter	ICD10CM
T39.4X4D	Poisoning by antirheumatics, not elsewhere classified, undetermined, subsequent encounter	ICD10CM
T39.4X4S	Poisoning by antirheumatics, not elsewhere classified, undetermined, sequela	ICD10CM
T39.8X4	Poisoning by other nonopioid analgesics and antipyretics, not elsewhere classified, undetermined	ICD10CM
T39.8X4A	Poisoning by other nonopioid analgesics and antipyretics, not elsewhere classified, undetermined, initial encounter	ICD10CM

© 2021 Walsh CG et al. *JAMA Network Open.*

T39.8X4D	Poisoning by other nonopioid analgesics and antipyretics, not elsewhere classified, undetermined, subsequent encounter	ICD10CM
T39.8X4S	Poisoning by other nonopioid analgesics and antipyretics, not elsewhere classified, undetermined, sequela	ICD10CM
T39.94	Poisoning by unspecified nonopioid analgesic, antipyretic and antirheumatic, undetermined	ICD10CM
T39.94XA	Poisoning by unspecified nonopioid analgesic, antipyretic and antirheumatic, undetermined, initial encounter	ICD10CM
T39.94XD	Poisoning by unspecified nonopioid analgesic, antipyretic and antirheumatic, undetermined, subsequent encounter	ICD10CM
T39.94XS	Poisoning by unspecified nonopioid analgesic, antipyretic and antirheumatic, undetermined, sequela	ICD10CM
T40.0X4	Poisoning by opium, undetermined	ICD10CM
T40.0X4A	Poisoning by opium, undetermined, initial encounter	ICD10CM
T40.0X4D	Poisoning by opium, undetermined, subsequent encounter	ICD10CM
T40.0X4S	Poisoning by opium, undetermined, sequela	ICD10CM
T40.1X4	Poisoning by heroin, undetermined	ICD10CM
T40.1X4A	Poisoning by heroin, undetermined, initial encounter	ICD10CM
T40.1X4D	Poisoning by heroin, undetermined, subsequent encounter	ICD10CM
T40.1X4S	Poisoning by heroin, undetermined, sequela	ICD10CM
T40.2X4	Poisoning by other opioids, undetermined	ICD10CM
T40.2X4A	Poisoning by other opioids, undetermined, initial encounter	ICD10CM
T40.2X4D	Poisoning by other opioids, undetermined, subsequent encounter	ICD10CM
T40.2X4S	Poisoning by other opioids, undetermined, sequela	ICD10CM
T40.3X4	Poisoning by methadone, undetermined	ICD10CM
T40.3X4A	Poisoning by methadone, undetermined, initial encounter	ICD10CM

© 2021 Walsh CG et al. *JAMA Network Open.*

T40.3X4D	Poisoning by methadone, undetermined, subsequent encounter	ICD10CM
T40.3X4S	Poisoning by methadone, undetermined, sequela	ICD10CM
T40.4X4	Poisoning by other synthetic narcotics, undetermined	ICD10CM
T40.4X4A	Poisoning by other synthetic narcotics, undetermined, initial encounter	ICD10CM
T40.4X4D	Poisoning by other synthetic narcotics, undetermined, subsequent encounter	ICD10CM
T40.4X4S	Poisoning by other synthetic narcotics, undetermined, sequela	ICD10CM
T40.5X4	Poisoning by cocaine, undetermined	ICD10CM
T40.5X4A	Poisoning by cocaine, undetermined, initial encounter	ICD10CM
T40.5X4D	Poisoning by cocaine, undetermined, subsequent encounter	ICD10CM
T40.5X4S	Poisoning by cocaine, undetermined, sequela	ICD10CM
T40.604	Poisoning by unspecified narcotics, undetermined	ICD10CM
T40.604A	Poisoning by unspecified narcotics, undetermined, initial encounter	ICD10CM
T40.604D	Poisoning by unspecified narcotics, undetermined, subsequent encounter	ICD10CM
T40.604S	Poisoning by unspecified narcotics, undetermined, sequela	ICD10CM
T40.694	Poisoning by other narcotics, undetermined	ICD10CM
T40.694A	Poisoning by other narcotics, undetermined, initial encounter	ICD10CM
T40.694D	Poisoning by other narcotics, undetermined, subsequent encounter	ICD10CM
T40.694S	Poisoning by other narcotics, undetermined, sequela	ICD10CM
T40.7X4	Poisoning by cannabis (derivatives), undetermined	ICD10CM
T40.7X4A	Poisoning by cannabis (derivatives), undetermined, initial encounter	ICD10CM
T40.7X4D	Poisoning by cannabis (derivatives), undetermined, subsequent encounter	ICD10CM
T40.7X4S	Poisoning by cannabis (derivatives), undetermined, sequela	ICD10CM

© 2021 Walsh CG et al. *JAMA Network Open.*

T40.8X4	Poisoning by lysergide [LSD], undetermined	ICD10CM
T40.8X4A	Poisoning by lysergide [LSD], undetermined, initial encounter	ICD10CM
T40.8X4D	Poisoning by lysergide [LSD], undetermined, subsequent encounter	ICD10CM
T40.8X4S	Poisoning by lysergide [LSD], undetermined, sequela	ICD10CM
T40.904	Poisoning by unspecified psychodysleptics [hallucinogens], undetermined	ICD10CM
T40.904A	Poisoning by unspecified psychodysleptics [hallucinogens], undetermined, initial encounter	ICD10CM
T40.904D	Poisoning by unspecified psychodysleptics [hallucinogens], undetermined, subsequent encounter	ICD10CM
T40.904S	Poisoning by unspecified psychodysleptics [hallucinogens], undetermined, sequela	ICD10CM
T40.994	Poisoning by other psychodysleptics [hallucinogens], undetermined	ICD10CM
T40.994A	Poisoning by other psychodysleptics [hallucinogens], undetermined, initial encounter	ICD10CM
T40.994D	Poisoning by other psychodysleptics [hallucinogens], undetermined, subsequent encounter	ICD10CM
T40.994S	Poisoning by other psychodysleptics [hallucinogens], undetermined, sequela	ICD10CM
T41.0X4	Poisoning by inhaled anesthetics, undetermined	ICD10CM
T41.0X4A	Poisoning by inhaled anesthetics, undetermined, initial encounter	ICD10CM
T41.0X4D	Poisoning by inhaled anesthetics, undetermined, subsequent encounter	ICD10CM
T41.0X4S	Poisoning by inhaled anesthetics, undetermined, sequela	ICD10CM
T41.1X4	Poisoning by intravenous anesthetics, undetermined	ICD10CM
T41.1X4A	Poisoning by intravenous anesthetics, undetermined, initial encounter	ICD10CM
T41.1X4D	Poisoning by intravenous anesthetics, undetermined, subsequent encounter	ICD10CM
T41.1X4S	Poisoning by intravenous anesthetics, undetermined, sequela	ICD10CM
T41.204	Poisoning by unspecified general anesthetics, undetermined	ICD10CM

© 2021 Walsh CG et al. *JAMA Network Open.*

T41.204A	Poisoning by unspecified general anesthetics, undetermined, initial encounter	ICD10CM
T41.204D	Poisoning by unspecified general anesthetics, undetermined, subsequent encounter	ICD10CM
T41.204S	Poisoning by unspecified general anesthetics, undetermined, sequela	ICD10CM
T41.294	Poisoning by other general anesthetics, undetermined	ICD10CM
T41.294A	Poisoning by other general anesthetics, undetermined, initial encounter	ICD10CM
T41.294D	Poisoning by other general anesthetics, undetermined, subsequent encounter	ICD10CM
T41.294S	Poisoning by other general anesthetics, undetermined, sequela	ICD10CM
T41.3X4	Poisoning by local anesthetics, undetermined	ICD10CM
T41.3X4A	Poisoning by local anesthetics, undetermined, initial encounter	ICD10CM
T41.3X4D	Poisoning by local anesthetics, undetermined, subsequent encounter	ICD10CM
T41.3X4S	Poisoning by local anesthetics, undetermined, sequela	ICD10CM
T41.44	Poisoning by unspecified anesthetic, undetermined	ICD10CM
T41.44XA	Poisoning by unspecified anesthetic, undetermined, initial encounter	ICD10CM
T41.44XD	Poisoning by unspecified anesthetic, undetermined, subsequent encounter	ICD10CM
T41.44XS	Poisoning by unspecified anesthetic, undetermined, sequela	ICD10CM
T41.5X4	Poisoning by therapeutic gases, undetermined	ICD10CM
T41.5X4A	Poisoning by therapeutic gases, undetermined, initial encounter	ICD10CM
T41.5X4D	Poisoning by therapeutic gases, undetermined, subsequent encounter	ICD10CM
T41.5X4S	Poisoning by therapeutic gases, undetermined, sequela	ICD10CM
T42.0X4	Poisoning by hydantoin derivatives, undetermined	ICD10CM
T42.0X4A	Poisoning by hydantoin derivatives, undetermined, initial encounter	ICD10CM
T42.0X4D	Poisoning by hydantoin derivatives, undetermined, subsequent encounter	ICD10CM

© 2021 Walsh CG et al. *JAMA Network Open.*

T42.0X4S	Poisoning by hydantoin derivatives, undetermined, sequela	ICD10CM
T42.1X4	Poisoning by iminostilbenes, undetermined	ICD10CM
T42.1X4A	Poisoning by iminostilbenes, undetermined, initial encounter	ICD10CM
T42.1X4D	Poisoning by iminostilbenes, undetermined, subsequent encounter	ICD10CM
T42.1X4S	Poisoning by iminostilbenes, undetermined, sequela	ICD10CM
T42.2X4	Poisoning by succinimides and oxazolidinediones, undetermined	ICD10CM
T42.2X4A	Poisoning by succinimides and oxazolidinediones, undetermined, initial encounter	ICD10CM
T42.2X4D	Poisoning by succinimides and oxazolidinediones, undetermined, subsequent encounter	ICD10CM
T42.2X4S	Poisoning by succinimides and oxazolidinediones, undetermined, sequela	ICD10CM
T42.3X4	Poisoning by barbiturates, undetermined	ICD10CM
T42.3X4A	Poisoning by barbiturates, undetermined, initial encounter	ICD10CM
T42.3X4D	Poisoning by barbiturates, undetermined, subsequent encounter	ICD10CM
T42.3X4S	Poisoning by barbiturates, undetermined, sequela	ICD10CM
T42.4X4	Poisoning by benzodiazepines, undetermined	ICD10CM
T42.4X4A	Poisoning by benzodiazepines, undetermined, initial encounter	ICD10CM
T42.4X4D	Poisoning by benzodiazepines, undetermined, subsequent encounter	ICD10CM
T42.4X4S	Poisoning by benzodiazepines, undetermined, sequela	ICD10CM
T42.5X4	Poisoning by mixed antiepileptics, undetermined	ICD10CM
T42.5X4A	Poisoning by mixed antiepileptics, undetermined, initial encounter	ICD10CM
T42.5X4D	Poisoning by mixed antiepileptics, undetermined, subsequent encounter	ICD10CM
T42.5X4S	Poisoning by mixed antiepileptics, undetermined, sequela	ICD10CM
T42.6X4	Poisoning by other antiepileptic and sedative-hypnotic drugs, undetermined	ICD10CM

© 2021 Walsh CG et al. *JAMA Network Open.*

T42.6X4A	Poisoning by other antiepileptic and sedative-hypnotic drugs, undetermined, initial encounter	ICD10CM
T42.6X4D	Poisoning by other antiepileptic and sedative-hypnotic drugs, undetermined, subsequent encounter	ICD10CM
T42.6X4S	Poisoning by other antiepileptic and sedative-hypnotic drugs, undetermined, sequela	ICD10CM
T42.74	Poisoning by unspecified antiepileptic and sedative-hypnotic drugs, undetermined	ICD10CM
T42.74XA	Poisoning by unspecified antiepileptic and sedative-hypnotic drugs, undetermined, initial encounter	ICD10CM
T42.74XD	Poisoning by unspecified antiepileptic and sedative-hypnotic drugs, undetermined, subsequent encounter	ICD10CM
T42.74XS	Poisoning by unspecified antiepileptic and sedative-hypnotic drugs, undetermined, sequela	ICD10CM
T42.8X4	Poisoning by antiparkinsonism drugs and other central muscle-tone depressants, undetermined	ICD10CM
T42.8X4A	Poisoning by antiparkinsonism drugs and other central muscle-tone depressants, undetermined, initial encounter	ICD10CM
T42.8X4D	Poisoning by antiparkinsonism drugs and other central muscle-tone depressants, undetermined, subsequent encounter	ICD10CM
T42.8X4S	Poisoning by antiparkinsonism drugs and other central muscle-tone depressants, undetermined, sequela	ICD10CM
T43.014	Poisoning by tricyclic antidepressants, undetermined	ICD10CM
T43.014A	Poisoning by tricyclic antidepressants, undetermined, initial encounter	ICD10CM
T43.014D	Poisoning by tricyclic antidepressants, undetermined, subsequent encounter	ICD10CM
T43.014S	Poisoning by tricyclic antidepressants, undetermined, sequela	ICD10CM
T43.024	Poisoning by tetracyclic antidepressants, undetermined	ICD10CM
T43.024A	Poisoning by tetracyclic antidepressants, undetermined, initial encounter	ICD10CM
T43.024D	Poisoning by tetracyclic antidepressants, undetermined, subsequent encounter	ICD10CM
T43.024S	Poisoning by tetracyclic antidepressants, undetermined, sequela	ICD10CM

© 2021 Walsh CG et al. *JAMA Network Open.*

T43.0x4A	Poisoning by tricyclic and tetracyclic antidepressants, undetermined, initial encounter	ICD10CM
T43.0x4D	Poisoning by tricyclic and tetracyclic antidepressants, undetermined, subsequent encounter	ICD10CM
T43.0x4S	Poisoning by tricyclic and tetracyclic antidepressants, undetermined, sequela	ICD10CM
T43.1X4	Poisoning by monoamine-oxidase-inhibitor antidepressants, undetermined	ICD10CM
T43.1X4A	Poisoning by monoamine-oxidase-inhibitor antidepressants, undetermined, initial encounter	ICD10CM
T43.1X4D	Poisoning by monoamine-oxidase-inhibitor antidepressants, undetermined, subsequent encounter	ICD10CM
T43.1X4S	Poisoning by monoamine-oxidase-inhibitor antidepressants, undetermined, sequela	ICD10CM
T43.204	Poisoning by unspecified antidepressants, undetermined	ICD10CM
T43.204A	Poisoning by unspecified antidepressants, undetermined, initial encounter	ICD10CM
T43.204D	Poisoning by unspecified antidepressants, undetermined, subsequent encounter	ICD10CM
T43.204S	Poisoning by unspecified antidepressants, undetermined, sequela	ICD10CM
T43.214	Poisoning by selective serotonin and norepinephrine reuptake inhibitors, undetermined	ICD10CM
T43.214A	Poisoning by selective serotonin and norepinephrine reuptake inhibitors, undetermined, initial encounter	ICD10CM
T43.214D	Poisoning by selective serotonin and norepinephrine reuptake inhibitors, undetermined, subsequent encounter	ICD10CM
T43.214S	Poisoning by selective serotonin and norepinephrine reuptake inhibitors, undetermined, sequela	ICD10CM
T43.224	Poisoning by selective serotonin reuptake inhibitors, undetermined	ICD10CM
T43.224A	Poisoning by selective serotonin reuptake inhibitors, undetermined, initial encounter	ICD10CM
T43.224D	Poisoning by selective serotonin reuptake inhibitors, undetermined, subsequent encounter	ICD10CM
T43.224S	Poisoning by selective serotonin reuptake inhibitors, undetermined, sequela	ICD10CM

© 2021 Walsh CG et al. *JAMA Network Open.*

**T43.294**	Poisoning by other antidepressants, undetermined	ICD10CM
**T43.294A**	Poisoning by other antidepressants, undetermined, initial encounter	ICD10CM
**T43.294D**	Poisoning by other antidepressants, undetermined, subsequent encounter	ICD10CM
**T43.294S**	Poisoning by other antidepressants, undetermined, sequela	ICD10CM
**T43.3X4**	Poisoning by phenothiazine antipsychotics and neuroleptics, undetermined	ICD10CM
**T43.3X4A**	Poisoning by phenothiazine antipsychotics and neuroleptics, undetermined, initial encounter	ICD10CM
**T43.3X4D**	Poisoning by phenothiazine antipsychotics and neuroleptics, undetermined, subsequent encounter	ICD10CM
**T43.3X4S**	Poisoning by phenothiazine antipsychotics and neuroleptics, undetermined, sequela	ICD10CM
**T43.4X4**	Poisoning by butyrophenone and thiothixene neuroleptics, undetermined	ICD10CM
**T43.4X4A**	Poisoning by butyrophenone and thiothixene neuroleptics, undetermined, initial encounter	ICD10CM
**T43.4X4D**	Poisoning by butyrophenone and thiothixene neuroleptics, undetermined, subsequent encounter	ICD10CM
**T43.4X4S**	Poisoning by butyrophenone and thiothixene neuroleptics, undetermined, sequela	ICD10CM
**T43.504**	Poisoning by unspecified antipsychotics and neuroleptics, undetermined	ICD10CM
**T43.504A**	Poisoning by unspecified antipsychotics and neuroleptics, undetermined, initial encounter	ICD10CM
**T43.504D**	Poisoning by unspecified antipsychotics and neuroleptics, undetermined, subsequent encounter	ICD10CM
**T43.504S**	Poisoning by unspecified antipsychotics and neuroleptics, undetermined, sequela	ICD10CM
**T43.594**	Poisoning by other antipsychotics and neuroleptics, undetermined	ICD10CM
**T43.594A**	Poisoning by other antipsychotics and neuroleptics, undetermined, initial encounter	ICD10CM
**T43.594D**	Poisoning by other antipsychotics and neuroleptics, undetermined, subsequent encounter	ICD10CM
**T43.594S**	Poisoning by other antipsychotics and neuroleptics, undetermined, sequela	ICD10CM

© 2021 Walsh CG et al. *JAMA Network Open.*

**T43.604**	Poisoning by unspecified psychostimulants, undetermined	ICD10CM
**T43.604A**	Poisoning by unspecified psychostimulants, undetermined, initial encounter	ICD10CM
**T43.604D**	Poisoning by unspecified psychostimulants, undetermined, subsequent encounter	ICD10CM
**T43.604S**	Poisoning by unspecified psychostimulants, undetermined, sequela	ICD10CM
**T43.614**	Poisoning by caffeine, undetermined	ICD10CM
**T43.614A**	Poisoning by caffeine, undetermined, initial encounter	ICD10CM
**T43.614D**	Poisoning by caffeine, undetermined, subsequent encounter	ICD10CM
**T43.614S**	Poisoning by caffeine, undetermined, sequela	ICD10CM
**T43.624**	Poisoning by amphetamines, undetermined	ICD10CM
**T43.624A**	Poisoning by amphetamines, undetermined, initial encounter	ICD10CM
**T43.624D**	Poisoning by amphetamines, undetermined, subsequent encounter	ICD10CM
**T43.624S**	Poisoning by amphetamines, undetermined, sequela	ICD10CM
**T43.634**	Poisoning by methylphenidate, undetermined	ICD10CM
**T43.634A**	Poisoning by methylphenidate, undetermined, initial encounter	ICD10CM
**T43.634D**	Poisoning by methylphenidate, undetermined, subsequent encounter	ICD10CM
**T43.634S**	Poisoning by methylphenidate, undetermined, sequela	ICD10CM
**T43.644**	Poisoning by ecstasy, undetermined	ICD10CM
**T43.644A**	Poisoning by ecstasy, undetermined, initial encounter	ICD10CM
**T43.644D**	Poisoning by ecstasy, undetermined, subsequent encounter	ICD10CM
**T43.644S**	Poisoning by ecstasy, undetermined, sequela	ICD10CM
**T43.694**	Poisoning by other psychostimulants, undetermined	ICD10CM
**T43.694A**	Poisoning by other psychostimulants, undetermined, initial encounter	ICD10CM

© 2021 Walsh CG et al. *JAMA Network Open.*

**T43.694D**	Poisoning by other psychostimulants, undetermined, subsequent encounter	ICD10CM
**T43.694S**	Poisoning by other psychostimulants, undetermined, sequela	ICD10CM
**T43.6x4A**	Poisoning by psychostimulants with abuse potential, undetermined, initial encounter	ICD10CM
**T43.6x4D**	Poisoning by psychostimulants with abuse potential, undetermined, subsequent encounter	ICD10CM
**T43.6x4S**	Poisoning by psychostimulants with abuse potential, undetermined, sequela	ICD10CM
**T43.8X4**	Poisoning by other psychotropic drugs, undetermined	ICD10CM
**T43.8X4A**	Poisoning by other psychotropic drugs, undetermined, initial encounter	ICD10CM
**T43.8X4D**	Poisoning by other psychotropic drugs, undetermined, subsequent encounter	ICD10CM
**T43.8X4S**	Poisoning by other psychotropic drugs, undetermined, sequela	ICD10CM
**T43.94**	Poisoning by unspecified psychotropic drug, undetermined	ICD10CM
**T43.94XA**	Poisoning by unspecified psychotropic drug, undetermined, initial encounter	ICD10CM
**T43.94XD**	Poisoning by unspecified psychotropic drug, undetermined, subsequent encounter	ICD10CM
**T43.94XS**	Poisoning by unspecified psychotropic drug, undetermined, sequela	ICD10CM
**T44.0X4**	Poisoning by anticholinesterase agents, undetermined	ICD10CM
**T44.0X4A**	Poisoning by anticholinesterase agents, undetermined, initial encounter	ICD10CM
**T44.0X4D**	Poisoning by anticholinesterase agents, undetermined, subsequent encounter	ICD10CM
**T44.0X4S**	Poisoning by anticholinesterase agents, undetermined, sequela	ICD10CM
**T44.1X4**	Poisoning by other parasympathomimetics [cholinergics], undetermined	ICD10CM
**T44.1X4A**	Poisoning by other parasympathomimetics [cholinergics], undetermined, initial encounter	ICD10CM
**T44.1X4D**	Poisoning by other parasympathomimetics [cholinergics], undetermined, subsequent encounter	ICD10CM
**T44.1X4S**	Poisoning by other parasympathomimetics [cholinergics], undetermined, sequela	ICD10CM

© 2021 Walsh CG et al. *JAMA Network Open.*

T44.2X4	Poisoning by ganglionic blocking drugs, undetermined	ICD10CM
T44.2X4A	Poisoning by ganglionic blocking drugs, undetermined, initial encounter	ICD10CM
T44.2X4D	Poisoning by ganglionic blocking drugs, undetermined, subsequent encounter	ICD10CM
T44.2X4S	Poisoning by ganglionic blocking drugs, undetermined, sequela	ICD10CM
T44.3X4	Poisoning by other parasympatholytics [anticholinergics and antimuscarinics] and spasmolytics, undetermined	ICD10CM
T44.3X4A	Poisoning by other parasympatholytics [anticholinergics and antimuscarinics] and spasmolytics, undetermined, initial encounter	ICD10CM
T44.3X4D	Poisoning by other parasympatholytics [anticholinergics and antimuscarinics] and spasmolytics, undetermined, subsequent encounter	ICD10CM
T44.3X4S	Poisoning by other parasympatholytics [anticholinergics and antimuscarinics] and spasmolytics, undetermined, sequela	ICD10CM
T44.4X4	Poisoning by predominantly alpha-adrenoreceptor agonists, undetermined	ICD10CM
T44.4X4A	Poisoning by predominantly alpha-adrenoreceptor agonists, undetermined, initial encounter	ICD10CM
T44.4X4D	Poisoning by predominantly alpha-adrenoreceptor agonists, undetermined, subsequent encounter	ICD10CM
T44.4X4S	Poisoning by predominantly alpha-adrenoreceptor agonists, undetermined, sequela	ICD10CM
T44.5X4	Poisoning by predominantly beta-adrenoreceptor agonists, undetermined	ICD10CM
T44.5X4A	Poisoning by predominantly beta-adrenoreceptor agonists, undetermined, initial encounter	ICD10CM
T44.5X4D	Poisoning by predominantly beta-adrenoreceptor agonists, undetermined, subsequent encounter	ICD10CM
T44.5X4S	Poisoning by predominantly beta-adrenoreceptor agonists, undetermined, sequela	ICD10CM
T44.6X4	Poisoning by alpha-adrenoreceptor antagonists, undetermined	ICD10CM
T44.6X4A	Poisoning by alpha-adrenoreceptor antagonists, undetermined, initial encounter	ICD10CM
T44.6X4D	Poisoning by alpha-adrenoreceptor antagonists, undetermined, subsequent encounter	ICD10CM
T44.6X4S	Poisoning by alpha-adrenoreceptor antagonists, undetermined, sequela	ICD10CM

© 2021 Walsh CG et al. *JAMA Network Open.*

**T44.7X4**	Poisoning by beta-adrenoreceptor antagonists, undetermined	ICD10CM
**T44.7X4A**	Poisoning by beta-adrenoreceptor antagonists, undetermined, initial encounter	ICD10CM
**T44.7X4D**	Poisoning by beta-adrenoreceptor antagonists, undetermined, subsequent encounter	ICD10CM
**T44.7X4S**	Poisoning by beta-adrenoreceptor antagonists, undetermined, sequela	ICD10CM
**T44.8X4**	Poisoning by centrally-acting and adrenergic-neuron-blocking agents, undetermined	ICD10CM
**T44.8X4A**	Poisoning by centrally-acting and adrenergic-neuron-blocking agents, undetermined, initial encounter	ICD10CM
**T44.8X4D**	Poisoning by centrally-acting and adrenergic-neuron-blocking agents, undetermined, subsequent encounter	ICD10CM
**T44.8X4S**	Poisoning by centrally-acting and adrenergic-neuron-blocking agents, undetermined, sequela	ICD10CM
**T44.904**	Poisoning by unspecified drugs primarily affecting the autonomic nervous system, undetermined	ICD10CM
**T44.904A**	Poisoning by unspecified drugs primarily affecting the autonomic nervous system, undetermined, initial encounter	ICD10CM
**T44.904D**	Poisoning by unspecified drugs primarily affecting the autonomic nervous system, undetermined, subsequent encounter	ICD10CM
**T44.904S**	Poisoning by unspecified drugs primarily affecting the autonomic nervous system, undetermined, sequela	ICD10CM
**T44.994**	Poisoning by other drug primarily affecting the autonomic nervous system, undetermined	ICD10CM
**T44.994A**	Poisoning by other drug primarily affecting the autonomic nervous system, undetermined, initial encounter	ICD10CM
**T44.994D**	Poisoning by other drug primarily affecting the autonomic nervous system, undetermined, subsequent encounter	ICD10CM
**T44.994S**	Poisoning by other drug primarily affecting the autonomic nervous system, undetermined, sequela	ICD10CM
**T45.0X4**	Poisoning by antiallergic and antiemetic drugs, undetermined	ICD10CM
**T45.0X4A**	Poisoning by antiallergic and antiemetic drugs, undetermined, initial encounter	ICD10CM

© 2021 Walsh CG et al. *JAMA Network Open.*

T45.0X4D	Poisoning by antiallergic and antiemetic drugs, undetermined, subsequent encounter	ICD10CM
T45.0X4S	Poisoning by antiallergic and antiemetic drugs, undetermined, sequela	ICD10CM
T45.1X4	Poisoning by antineoplastic and immunosuppressive drugs, undetermined	ICD10CM
T45.1X4A	Poisoning by antineoplastic and immunosuppressive drugs, undetermined, initial encounter	ICD10CM
T45.1X4D	Poisoning by antineoplastic and immunosuppressive drugs, undetermined, subsequent encounter	ICD10CM
T45.1X4S	Poisoning by antineoplastic and immunosuppressive drugs, undetermined, sequela	ICD10CM
T45.2X4	Poisoning by vitamins, undetermined	ICD10CM
T45.2X4A	Poisoning by vitamins, undetermined, initial encounter	ICD10CM
T45.2X4D	Poisoning by vitamins, undetermined, subsequent encounter	ICD10CM
T45.2X4S	Poisoning by vitamins, undetermined, sequela	ICD10CM
T45.3X4	Poisoning by enzymes, undetermined	ICD10CM
T45.3X4A	Poisoning by enzymes, undetermined, initial encounter	ICD10CM
T45.3X4D	Poisoning by enzymes, undetermined, subsequent encounter	ICD10CM
T45.3X4S	Poisoning by enzymes, undetermined, sequela	ICD10CM
T45.4X4	Poisoning by iron and its compounds, undetermined	ICD10CM
T45.4X4A	Poisoning by iron and its compounds, undetermined, initial encounter	ICD10CM
T45.4X4D	Poisoning by iron and its compounds, undetermined, subsequent encounter	ICD10CM
T45.4X4S	Poisoning by iron and its compounds, undetermined, sequela	ICD10CM
T45.514	Poisoning by anticoagulants, undetermined	ICD10CM
T45.514A	Poisoning by anticoagulants, undetermined, initial encounter	ICD10CM
T45.514D	Poisoning by anticoagulants, undetermined, subsequent encounter	ICD10CM

© 2021 Walsh CG et al. *JAMA Network Open.*

**T45.514S**	Poisoning by anticoagulants, undetermined, sequela	ICD10CM
**T45.524**	Poisoning by antithrombotic drugs, undetermined	ICD10CM
**T45.524A**	Poisoning by antithrombotic drugs, undetermined, initial encounter	ICD10CM
**T45.524D**	Poisoning by antithrombotic drugs, undetermined, subsequent encounter	ICD10CM
**T45.524S**	Poisoning by antithrombotic drugs, undetermined, sequela	ICD10CM
**T45.604**	Poisoning by unspecified fibrinolysis-affecting drugs, undetermined	ICD10CM
**T45.604A**	Poisoning by unspecified fibrinolysis-affecting drugs, undetermined, initial encounter	ICD10CM
**T45.604D**	Poisoning by unspecified fibrinolysis-affecting drugs, undetermined, subsequent encounter	ICD10CM
**T45.604S**	Poisoning by unspecified fibrinolysis-affecting drugs, undetermined, sequela	ICD10CM
**T45.614**	Poisoning by thrombolytic drug, undetermined	ICD10CM
**T45.614A**	Poisoning by thrombolytic drug, undetermined, initial encounter	ICD10CM
**T45.614D**	Poisoning by thrombolytic drug, undetermined, subsequent encounter	ICD10CM
**T45.614S**	Poisoning by thrombolytic drug, undetermined, sequela	ICD10CM
**T45.624**	Poisoning by hemostatic drug, undetermined	ICD10CM
**T45.624A**	Poisoning by hemostatic drug, undetermined, initial encounter	ICD10CM
**T45.624D**	Poisoning by hemostatic drug, undetermined, subsequent encounter	ICD10CM
**T45.624S**	Poisoning by hemostatic drug, undetermined, sequela	ICD10CM
**T45.694**	Poisoning by other fibrinolysis-affecting drugs, undetermined	ICD10CM
**T45.694A**	Poisoning by other fibrinolysis-affecting drugs, undetermined, initial encounter	ICD10CM
**T45.694D**	Poisoning by other fibrinolysis-affecting drugs, undetermined, subsequent encounter	ICD10CM
**T45.694S**	Poisoning by other fibrinolysis-affecting drugs, undetermined, sequela	ICD10CM

© 2021 Walsh CG et al. *JAMA Network Open.*

**T45.7X4**	Poisoning by anticoagulant antagonists, vitamin K and other coagulants, undetermined	ICD10CM
**T45.7X4A**	Poisoning by anticoagulant antagonists, vitamin K and other coagulants, undetermined, initial encounter	ICD10CM
**T45.7X4D**	Poisoning by anticoagulant antagonists, vitamin K and other coagulants, undetermined, subsequent encounter	ICD10CM
**T45.7X4S**	Poisoning by anticoagulant antagonists, vitamin K and other coagulants, undetermined, sequela	ICD10CM
**T45.8X4**	Poisoning by other primarily systemic and hematological agents, undetermined	ICD10CM
**T45.8X4A**	Poisoning by other primarily systemic and hematological agents, undetermined, initial encounter	ICD10CM
**T45.8X4D**	Poisoning by other primarily systemic and hematological agents, undetermined, subsequent encounter	ICD10CM
**T45.8X4S**	Poisoning by other primarily systemic and hematological agents, undetermined, sequela	ICD10CM
**T45.94**	Poisoning by unspecified primarily systemic and hematological agent, undetermined	ICD10CM
**T45.94XA**	Poisoning by unspecified primarily systemic and hematological agent, undetermined, initial encounter	ICD10CM
**T45.94XD**	Poisoning by unspecified primarily systemic and hematological agent, undetermined, subsequent encounter	ICD10CM
**T45.94XS**	Poisoning by unspecified primarily systemic and hematological agent, undetermined, sequela	ICD10CM
**T46.0X4**	Poisoning by cardiac-stimulant glycosides and drugs of similar action, undetermined	ICD10CM
**T46.0X4A**	Poisoning by cardiac-stimulant glycosides and drugs of similar action, undetermined, initial encounter	ICD10CM
**T46.0X4D**	Poisoning by cardiac-stimulant glycosides and drugs of similar action, undetermined, subsequent encounter	ICD10CM
**T46.0X4S**	Poisoning by cardiac-stimulant glycosides and drugs of similar action, undetermined, sequela	ICD10CM
**T46.1X4**	Poisoning by calcium-channel blockers, undetermined	ICD10CM
**T46.1X4A**	Poisoning by calcium-channel blockers, undetermined, initial encounter	ICD10CM

© 2021 Walsh CG et al. *JAMA Network Open.*

**T46.1X4D**	Poisoning by calcium-channel blockers, undetermined, subsequent encounter	ICD10CM
**T46.1X4S**	Poisoning by calcium-channel blockers, undetermined, sequela	ICD10CM
**T46.2X4**	Poisoning by other antidysrhythmic drugs, undetermined	ICD10CM
**T46.2X4A**	Poisoning by other antidysrhythmic drugs, undetermined, initial encounter	ICD10CM
**T46.2X4D**	Poisoning by other antidysrhythmic drugs, undetermined, subsequent encounter	ICD10CM
**T46.2X4S**	Poisoning by other antidysrhythmic drugs, undetermined, sequela	ICD10CM
**T46.3X4**	Poisoning by coronary vasodilators, undetermined	ICD10CM
**T46.3X4A**	Poisoning by coronary vasodilators, undetermined, initial encounter	ICD10CM
**T46.3X4D**	Poisoning by coronary vasodilators, undetermined, subsequent encounter	ICD10CM
**T46.3X4S**	Poisoning by coronary vasodilators, undetermined, sequela	ICD10CM
**T46.4X4**	Poisoning by angiotensin-converting-enzyme inhibitors, undetermined	ICD10CM
**T46.4X4A**	Poisoning by angiotensin-converting-enzyme inhibitors, undetermined, initial encounter	ICD10CM
**T46.4X4D**	Poisoning by angiotensin-converting-enzyme inhibitors, undetermined, subsequent encounter	ICD10CM
**T46.4X4S**	Poisoning by angiotensin-converting-enzyme inhibitors, undetermined, sequela	ICD10CM
**T46.5X4**	Poisoning by other antihypertensive drugs, undetermined	ICD10CM
**T46.5X4A**	Poisoning by other antihypertensive drugs, undetermined, initial encounter	ICD10CM
**T46.5X4D**	Poisoning by other antihypertensive drugs, undetermined, subsequent encounter	ICD10CM
**T46.5X4S**	Poisoning by other antihypertensive drugs, undetermined, sequela	ICD10CM
**T46.6X4**	Poisoning by antihyperlipidemic and antiarteriosclerotic drugs, undetermined	ICD10CM
**T46.6X4A**	Poisoning by antihyperlipidemic and antiarteriosclerotic drugs, undetermined, initial encounter	ICD10CM
**T46.6X4D**	Poisoning by antihyperlipidemic and antiarteriosclerotic drugs, undetermined, subsequent encounter	ICD10CM

**T46.6X4S**	Poisoning by antihyperlipidemic and antiarteriosclerotic drugs, undetermined, sequela	ICD10CM
**T46.7X4**	Poisoning by peripheral vasodilators, undetermined	ICD10CM
**T46.7X4A**	Poisoning by peripheral vasodilators, undetermined, initial encounter	ICD10CM
**T46.7X4D**	Poisoning by peripheral vasodilators, undetermined, subsequent encounter	ICD10CM
**T46.7X4S**	Poisoning by peripheral vasodilators, undetermined, sequela	ICD10CM
**T46.8X4**	Poisoning by antivaricose drugs, including sclerosing agents, undetermined	ICD10CM
**T46.8X4A**	Poisoning by antivaricose drugs, including sclerosing agents, undetermined, initial encounter	ICD10CM
**T46.8X4D**	Poisoning by antivaricose drugs, including sclerosing agents, undetermined, subsequent encounter	ICD10CM
**T46.8X4S**	Poisoning by antivaricose drugs, including sclerosing agents, undetermined, sequela	ICD10CM
**T46.904**	Poisoning by unspecified agents primarily affecting the cardiovascular system, undetermined	ICD10CM
**T46.904A**	Poisoning by unspecified agents primarily affecting the cardiovascular system, undetermined, initial encounter	ICD10CM
**T46.904D**	Poisoning by unspecified agents primarily affecting the cardiovascular system, undetermined, subsequent encounter	ICD10CM
**T46.904S**	Poisoning by unspecified agents primarily affecting the cardiovascular system, undetermined, sequela	ICD10CM
**T46.994**	Poisoning by other agents primarily affecting the cardiovascular system, undetermined	ICD10CM
**T46.994A**	Poisoning by other agents primarily affecting the cardiovascular system, undetermined, initial encounter	ICD10CM
**T46.994D**	Poisoning by other agents primarily affecting the cardiovascular system, undetermined, subsequent encounter	ICD10CM
**T46.994S**	Poisoning by other agents primarily affecting the cardiovascular system, undetermined, sequela	ICD10CM
**T47.0X4**	Poisoning by histamine H2-receptor blockers, undetermined	ICD10CM
**T47.0X4A**	Poisoning by histamine H2-receptor blockers, undetermined, initial encounter	ICD10CM

© 2021 Walsh CG et al. *JAMA Network Open.*

T47.0X4D	Poisoning by histamine H2-receptor blockers, undetermined, subsequent encounter	ICD10CM
T47.0X4S	Poisoning by histamine H2-receptor blockers, undetermined, sequela	ICD10CM
T47.1X4	Poisoning by other antacids and anti-gastric-secretion drugs, undetermined	ICD10CM
T47.1X4A	Poisoning by other antacids and anti-gastric-secretion drugs, undetermined, initial encounter	ICD10CM
T47.1X4D	Poisoning by other antacids and anti-gastric-secretion drugs, undetermined, subsequent encounter	ICD10CM
T47.1X4S	Poisoning by other antacids and anti-gastric-secretion drugs, undetermined, sequela	ICD10CM
T47.2X4	Poisoning by stimulant laxatives, undetermined	ICD10CM
T47.2X4A	Poisoning by stimulant laxatives, undetermined, initial encounter	ICD10CM
T47.2X4D	Poisoning by stimulant laxatives, undetermined, subsequent encounter	ICD10CM
T47.2X4S	Poisoning by stimulant laxatives, undetermined, sequela	ICD10CM
T47.3X4	Poisoning by saline and osmotic laxatives, undetermined	ICD10CM
T47.3X4A	Poisoning by saline and osmotic laxatives, undetermined, initial encounter	ICD10CM
T47.3X4D	Poisoning by saline and osmotic laxatives, undetermined, subsequent encounter	ICD10CM
T47.3X4S	Poisoning by saline and osmotic laxatives, undetermined, sequela	ICD10CM
T47.4X4	Poisoning by other laxatives, undetermined	ICD10CM
T47.4X4A	Poisoning by other laxatives, undetermined, initial encounter	ICD10CM
T47.4X4D	Poisoning by other laxatives, undetermined, subsequent encounter	ICD10CM
T47.4X4S	Poisoning by other laxatives, undetermined, sequela	ICD10CM
T47.5X4	Poisoning by digestants, undetermined	ICD10CM
T47.5X4A	Poisoning by digestants, undetermined, initial encounter	ICD10CM
T47.5X4D	Poisoning by digestants, undetermined, subsequent encounter	ICD10CM
T47.5X4S	Poisoning by digestants, undetermined, sequela	ICD10CM

© 2021 Walsh CG et al. *JAMA Network Open.*

T47.6X4	Poisoning by antidiarrheal drugs, undetermined	ICD10CM
T47.6X4A	Poisoning by antidiarrheal drugs, undetermined, initial encounter	ICD10CM
T47.6X4D	Poisoning by antidiarrheal drugs, undetermined, subsequent encounter	ICD10CM
T47.6X4S	Poisoning by antidiarrheal drugs, undetermined, sequela	ICD10CM
T47.7X4	Poisoning by emetics, undetermined	ICD10CM
T47.7X4A	Poisoning by emetics, undetermined, initial encounter	ICD10CM
T47.7X4D	Poisoning by emetics, undetermined, subsequent encounter	ICD10CM
T47.7X4S	Poisoning by emetics, undetermined, sequela	ICD10CM
T47.8X4	Poisoning by other agents primarily affecting gastrointestinal system, undetermined	ICD10CM
T47.8X4A	Poisoning by other agents primarily affecting gastrointestinal system, undetermined, initial encounter	ICD10CM
T47.8X4D	Poisoning by other agents primarily affecting gastrointestinal system, undetermined, subsequent encounter	ICD10CM
T47.8X4S	Poisoning by other agents primarily affecting gastrointestinal system, undetermined, sequela	ICD10CM
T47.94	Poisoning by unspecified agents primarily affecting the gastrointestinal system, undetermined	ICD10CM
T47.94XA	Poisoning by unspecified agents primarily affecting the gastrointestinal system, undetermined, initial encounter	ICD10CM
T47.94XD	Poisoning by unspecified agents primarily affecting the gastrointestinal system, undetermined, subsequent encounter	ICD10CM
T47.94XS	Poisoning by unspecified agents primarily affecting the gastrointestinal system, undetermined, sequela	ICD10CM
T48.0X4	Poisoning by oxytocic drugs, undetermined	ICD10CM
T48.0X4A	Poisoning by oxytocic drugs, undetermined, initial encounter	ICD10CM
T48.0X4D	Poisoning by oxytocic drugs, undetermined, subsequent encounter	ICD10CM
T48.0X4S	Poisoning by oxytocic drugs, undetermined, sequela	ICD10CM

© 2021 Walsh CG et al. *JAMA Network Open.*

T48.1X4	Poisoning by skeletal muscle relaxants [neuromuscular blocking agents], undetermined	ICD10CM
T48.1X4A	Poisoning by skeletal muscle relaxants [neuromuscular blocking agents], undetermined, initial encounter	ICD10CM
T48.1X4D	Poisoning by skeletal muscle relaxants [neuromuscular blocking agents], undetermined, subsequent encounter	ICD10CM
T48.1X4S	Poisoning by skeletal muscle relaxants [neuromuscular blocking agents], undetermined, sequela	ICD10CM
T48.204	Poisoning by unspecified drugs acting on muscles, undetermined	ICD10CM
T48.204A	Poisoning by unspecified drugs acting on muscles, undetermined, initial encounter	ICD10CM
T48.204D	Poisoning by unspecified drugs acting on muscles, undetermined, subsequent encounter	ICD10CM
T48.204S	Poisoning by unspecified drugs acting on muscles, undetermined, sequela	ICD10CM
T48.294	Poisoning by other drugs acting on muscles, undetermined	ICD10CM
T48.294A	Poisoning by other drugs acting on muscles, undetermined, initial encounter	ICD10CM
T48.294D	Poisoning by other drugs acting on muscles, undetermined, subsequent encounter	ICD10CM
T48.294S	Poisoning by other drugs acting on muscles, undetermined, sequela	ICD10CM
T48.3X4	Poisoning by antitussives, undetermined	ICD10CM
T48.3X4A	Poisoning by antitussives, undetermined, initial encounter	ICD10CM
T48.3X4D	Poisoning by antitussives, undetermined, subsequent encounter	ICD10CM
T48.3X4S	Poisoning by antitussives, undetermined, sequela	ICD10CM
T48.4X4	Poisoning by expectorants, undetermined	ICD10CM
T48.4X4A	Poisoning by expectorants, undetermined, initial encounter	ICD10CM
T48.4X4D	Poisoning by expectorants, undetermined, subsequent encounter	ICD10CM
T48.4X4S	Poisoning by expectorants, undetermined, sequela	ICD10CM
T48.5X4	Poisoning by other anti-common-cold drugs, undetermined	ICD10CM

© 2021 Walsh CG et al. *JAMA Network Open.*

**T48.5X4A**	Poisoning by other anti-common-cold drugs, undetermined, initial encounter	ICD10CM
**T48.5X4D**	Poisoning by other anti-common-cold drugs, undetermined, subsequent encounter	ICD10CM
**T48.5X4S**	Poisoning by other anti-common-cold drugs, undetermined, sequela	ICD10CM
**T48.6X4**	Poisoning by antiasthmatics, undetermined	ICD10CM
**T48.6X4A**	Poisoning by antiasthmatics, undetermined, initial encounter	ICD10CM
**T48.6X4D**	Poisoning by antiasthmatics, undetermined, subsequent encounter	ICD10CM
**T48.6X4S**	Poisoning by antiasthmatics, undetermined, sequela	ICD10CM
**T48.904**	Poisoning by unspecified agents primarily acting on the respiratory system, undetermined	ICD10CM
**T48.904A**	Poisoning by unspecified agents primarily acting on the respiratory system, undetermined, initial encounter	ICD10CM
**T48.904D**	Poisoning by unspecified agents primarily acting on the respiratory system, undetermined, subsequent encounter	ICD10CM
**T48.904S**	Poisoning by unspecified agents primarily acting on the respiratory system, undetermined, sequela	ICD10CM
**T48.994**	Poisoning by other agents primarily acting on the respiratory system, undetermined	ICD10CM
**T48.994A**	Poisoning by other agents primarily acting on the respiratory system, undetermined, initial encounter	ICD10CM
**T48.994D**	Poisoning by other agents primarily acting on the respiratory system, undetermined, subsequent encounter	ICD10CM
**T48.994S**	Poisoning by other agents primarily acting on the respiratory system, undetermined, sequela	ICD10CM
**T49.0X4**	Poisoning by local antifungal, anti-infective and anti-inflammatory drugs, undetermined	ICD10CM
**T49.0X4A**	Poisoning by local antifungal, anti-infective and anti-inflammatory drugs, undetermined, initial encounter	ICD10CM
**T49.0X4D**	Poisoning by local antifungal, anti-infective and anti-inflammatory drugs, undetermined, subsequent encounter	ICD10CM
**T49.0X4S**	Poisoning by local antifungal, anti-infective and anti-inflammatory drugs, undetermined, sequela	ICD10CM

© 2021 Walsh CG et al. *JAMA Network Open.*

T49.1X4	Poisoning by antipruritics, undetermined	ICD10CM
T49.1X4A	Poisoning by antipruritics, undetermined, initial encounter	ICD10CM
T49.1X4D	Poisoning by antipruritics, undetermined, subsequent encounter	ICD10CM
T49.1X4S	Poisoning by antipruritics, undetermined, sequela	ICD10CM
T49.2X4	Poisoning by local astringents and local detergents, undetermined	ICD10CM
T49.2X4A	Poisoning by local astringents and local detergents, undetermined, initial encounter	ICD10CM
T49.2X4D	Poisoning by local astringents and local detergents, undetermined, subsequent encounter	ICD10CM
T49.2X4S	Poisoning by local astringents and local detergents, undetermined, sequela	ICD10CM
T49.3X4	Poisoning by emollients, demulcents and protectants, undetermined	ICD10CM
T49.3X4A	Poisoning by emollients, demulcents and protectants, undetermined, initial encounter	ICD10CM
T49.3X4D	Poisoning by emollients, demulcents and protectants, undetermined, subsequent encounter	ICD10CM
T49.3X4S	Poisoning by emollients, demulcents and protectants, undetermined, sequela	ICD10CM
T49.4X4	Poisoning by keratolytics, keratoplastics, and other hair treatment drugs and preparations, undetermined	ICD10CM
T49.4X4A	Poisoning by keratolytics, keratoplastics, and other hair treatment drugs and preparations, undetermined, initial encounter	ICD10CM
T49.4X4D	Poisoning by keratolytics, keratoplastics, and other hair treatment drugs and preparations, undetermined, subsequent encounter	ICD10CM
T49.4X4S	Poisoning by keratolytics, keratoplastics, and other hair treatment drugs and preparations, undetermined, sequela	ICD10CM
T49.5X4	Poisoning by ophthalmological drugs and preparations, undetermined	ICD10CM
T49.5X4A	Poisoning by ophthalmological drugs and preparations, undetermined, initial encounter	ICD10CM
T49.5X4D	Poisoning by ophthalmological drugs and preparations, undetermined, subsequent encounter	ICD10CM
T49.5X4S	Poisoning by ophthalmological drugs and preparations, undetermined, sequela	ICD10CM

© 2021 Walsh CG et al. *JAMA Network Open.*

T49.6X4	Poisoning by otorhinolaryngological drugs and preparations, undetermined	ICD10CM
T49.6X4A	Poisoning by otorhinolaryngological drugs and preparations, undetermined, initial encounter	ICD10CM
T49.6X4D	Poisoning by otorhinolaryngological drugs and preparations, undetermined, subsequent encounter	ICD10CM
T49.6X4S	Poisoning by otorhinolaryngological drugs and preparations, undetermined, sequela	ICD10CM
T49.7X4	Poisoning by dental drugs, topically applied, undetermined	ICD10CM
T49.7X4A	Poisoning by dental drugs, topically applied, undetermined, initial encounter	ICD10CM
T49.7X4D	Poisoning by dental drugs, topically applied, undetermined, subsequent encounter	ICD10CM
T49.7X4S	Poisoning by dental drugs, topically applied, undetermined, sequela	ICD10CM
T49.8X4	Poisoning by other topical agents, undetermined	ICD10CM
T49.8X4A	Poisoning by other topical agents, undetermined, initial encounter	ICD10CM
T49.8X4D	Poisoning by other topical agents, undetermined, subsequent encounter	ICD10CM
T49.8X4S	Poisoning by other topical agents, undetermined, sequela	ICD10CM
T49.94	Poisoning by unspecified topical agent, undetermined	ICD10CM
T49.94XA	Poisoning by unspecified topical agent, undetermined, initial encounter	ICD10CM
T49.94XD	Poisoning by unspecified topical agent, undetermined, subsequent encounter	ICD10CM
T49.94XS	Poisoning by unspecified topical agent, undetermined, sequela	ICD10CM
T50.0X4	Poisoning by mineralocorticoids and their antagonists, undetermined	ICD10CM
T50.0X4A	Poisoning by mineralocorticoids and their antagonists, undetermined, initial encounter	ICD10CM
T50.0X4D	Poisoning by mineralocorticoids and their antagonists, undetermined, subsequent encounter	ICD10CM
T50.0X4S	Poisoning by mineralocorticoids and their antagonists, undetermined, sequela	ICD10CM
T50.1X4	Poisoning by loop [high-ceiling] diuretics, undetermined	ICD10CM

© 2021 Walsh CG et al. *JAMA Network Open.*

T50.1X4A	Poisoning by loop [high-ceiling] diuretics, undetermined, initial encounter	ICD10CM
T50.1X4D	Poisoning by loop [high-ceiling] diuretics, undetermined, subsequent encounter	ICD10CM
T50.1X4S	Poisoning by loop [high-ceiling] diuretics, undetermined, sequela	ICD10CM
T50.2X4	Poisoning by carbonic-anhydrase inhibitors, benzothiadiazides and other diuretics, undetermined	ICD10CM
T50.2X4A	Poisoning by carbonic-anhydrase inhibitors, benzothiadiazides and other diuretics, undetermined, initial encounter	ICD10CM
T50.2X4D	Poisoning by carbonic-anhydrase inhibitors, benzothiadiazides and other diuretics, undetermined, subsequent encounter	ICD10CM
T50.2X4S	Poisoning by carbonic-anhydrase inhibitors, benzothiadiazides and other diuretics, undetermined, sequela	ICD10CM
T50.3X4	Poisoning by electrolytic, caloric and water-balance agents, undetermined	ICD10CM
T50.3X4A	Poisoning by electrolytic, caloric and water-balance agents, undetermined, initial encounter	ICD10CM
T50.3X4D	Poisoning by electrolytic, caloric and water-balance agents, undetermined, subsequent encounter	ICD10CM
T50.3X4S	Poisoning by electrolytic, caloric and water-balance agents, undetermined, sequela	ICD10CM
T50.4X4	Poisoning by drugs affecting uric acid metabolism, undetermined	ICD10CM
T50.4X4A	Poisoning by drugs affecting uric acid metabolism, undetermined, initial encounter	ICD10CM
T50.4X4D	Poisoning by drugs affecting uric acid metabolism, undetermined, subsequent encounter	ICD10CM
T50.4X4S	Poisoning by drugs affecting uric acid metabolism, undetermined, sequela	ICD10CM
T50.5X4	Poisoning by appetite depressants, undetermined	ICD10CM
T50.5X4A	Poisoning by appetite depressants, undetermined, initial encounter	ICD10CM
T50.5X4D	Poisoning by appetite depressants, undetermined, subsequent encounter	ICD10CM
T50.5X4S	Poisoning by appetite depressants, undetermined, sequela	ICD10CM
T50.6X4	Poisoning by antidotes and chelating agents, undetermined	ICD10CM

© 2021 Walsh CG et al. *JAMA Network Open.*

T50.6X4A	Poisoning by antidotes and chelating agents, undetermined, initial encounter	ICD10CM
T50.6X4D	Poisoning by antidotes and chelating agents, undetermined, subsequent encounter	ICD10CM
T50.6X4S	Poisoning by antidotes and chelating agents, undetermined, sequela	ICD10CM
T50.7X4	Poisoning by analeptics and opioid receptor antagonists, undetermined	ICD10CM
T50.7X4A	Poisoning by analeptics and opioid receptor antagonists, undetermined, initial encounter	ICD10CM
T50.7X4D	Poisoning by analeptics and opioid receptor antagonists, undetermined, subsequent encounter	ICD10CM
T50.7X4S	Poisoning by analeptics and opioid receptor antagonists, undetermined, sequela	ICD10CM
T50.8X4	Poisoning by diagnostic agents, undetermined	ICD10CM
T50.8X4A	Poisoning by diagnostic agents, undetermined, initial encounter	ICD10CM
T50.8X4D	Poisoning by diagnostic agents, undetermined, subsequent encounter	ICD10CM
T50.8X4S	Poisoning by diagnostic agents, undetermined, sequela	ICD10CM
T50.904	Poisoning by unspecified drugs, medicaments and biological substances, undetermined	ICD10CM
T50.904A	Poisoning by unspecified drugs, medicaments and biological substances, undetermined, initial encounter	ICD10CM
T50.904D	Poisoning by unspecified drugs, medicaments and biological substances, undetermined, subsequent encounter	ICD10CM
T50.904S	Poisoning by unspecified drugs, medicaments and biological substances, undetermined, sequela	ICD10CM
T50.994	Poisoning by other drugs, medicaments and biological substances, undetermined	ICD10CM
T50.994A	Poisoning by other drugs, medicaments and biological substances, undetermined, initial encounter	ICD10CM
T50.994D	Poisoning by other drugs, medicaments and biological substances, undetermined, subsequent encounter	ICD10CM
T50.994S	Poisoning by other drugs, medicaments and biological substances, undetermined, sequela	ICD10CM

© 2021 Walsh CG et al. *JAMA Network Open.*

T50.A14	Poisoning by pertussis vaccine, including combinations with a pertussis component, undetermined	ICD10CM
T50.A14A	Poisoning by pertussis vaccine, including combinations with a pertussis component, undetermined, initial encounter	ICD10CM
T50.A14D	Poisoning by pertussis vaccine, including combinations with a pertussis component, undetermined, subsequent encounter	ICD10CM
T50.A14S	Poisoning by pertussis vaccine, including combinations with a pertussis component, undetermined, sequela	ICD10CM
T50.A24	Poisoning by mixed bacterial vaccines without a pertussis component, undetermined	ICD10CM
T50.A24A	Poisoning by mixed bacterial vaccines without a pertussis component, undetermined, initial encounter	ICD10CM
T50.A24D	Poisoning by mixed bacterial vaccines without a pertussis component, undetermined, subsequent encounter	ICD10CM
T50.A24S	Poisoning by mixed bacterial vaccines without a pertussis component, undetermined, sequela	ICD10CM
T50.A94	Poisoning by other bacterial vaccines, undetermined	ICD10CM
T50.A94A	Poisoning by other bacterial vaccines, undetermined, initial encounter	ICD10CM
T50.A94D	Poisoning by other bacterial vaccines, undetermined, subsequent encounter	ICD10CM
T50.A94S	Poisoning by other bacterial vaccines, undetermined, sequela	ICD10CM
T50.B14	Poisoning by smallpox vaccines, undetermined	ICD10CM
T50.B14A	Poisoning by smallpox vaccines, undetermined, initial encounter	ICD10CM
T50.B14D	Poisoning by smallpox vaccines, undetermined, subsequent encounter	ICD10CM
T50.B14S	Poisoning by smallpox vaccines, undetermined, sequela	ICD10CM
T50.B94	Poisoning by other viral vaccines, undetermined	ICD10CM
T50.B94A	Poisoning by other viral vaccines, undetermined, initial encounter	ICD10CM
T50.B94D	Poisoning by other viral vaccines, undetermined, subsequent encounter	ICD10CM
T50.B94S	Poisoning by other viral vaccines, undetermined, sequela	ICD10CM

© 2021 Walsh CG et al. *JAMA Network Open.*

T50.Z14	Poisoning by immunoglobulin, undetermined	ICD10CM
T50.Z14A	Poisoning by immunoglobulin, undetermined, initial encounter	ICD10CM
T50.Z14D	Poisoning by immunoglobulin, undetermined, subsequent encounter	ICD10CM
T50.Z14S	Poisoning by immunoglobulin, undetermined, sequela	ICD10CM
T50.Z94	Poisoning by other vaccines and biological substances, undetermined	ICD10CM
T50.Z94A	Poisoning by other vaccines and biological substances, undetermined, initial encounter	ICD10CM
T50.Z94D	Poisoning by other vaccines and biological substances, undetermined, subsequent encounter	ICD10CM
T50.Z94S	Poisoning by other vaccines and biological substances, undetermined, sequela	ICD10CM
T51.0X4	Toxic effect of ethanol, undetermined	ICD10CM
T51.0X4A	Toxic effect of ethanol, undetermined, initial encounter	ICD10CM
T51.0X4D	Toxic effect of ethanol, undetermined, subsequent encounter	ICD10CM
T51.0X4S	Toxic effect of ethanol, undetermined, sequela	ICD10CM
T51.1X4	Toxic effect of methanol, undetermined	ICD10CM
T51.1X4A	Toxic effect of methanol, undetermined, initial encounter	ICD10CM
T51.1X4D	Toxic effect of methanol, undetermined, subsequent encounter	ICD10CM
T51.1X4S	Toxic effect of methanol, undetermined, sequela	ICD10CM
T51.2X4	Toxic effect of 2-Propanol, undetermined	ICD10CM
T51.2X4A	Toxic effect of 2-Propanol, undetermined, initial encounter	ICD10CM
T51.2X4D	Toxic effect of 2-Propanol, undetermined, subsequent encounter	ICD10CM
T51.2X4S	Toxic effect of 2-Propanol, undetermined, sequela	ICD10CM
T51.3X4	Toxic effect of fusel oil, undetermined	ICD10CM
T51.3X4A	Toxic effect of fusel oil, undetermined, initial encounter	ICD10CM

© 2021 Walsh CG et al. *JAMA Network Open.*

T51.3X4D	Toxic effect of fusel oil, undetermined, subsequent encounter	ICD10CM
T51.3X4S	Toxic effect of fusel oil, undetermined, sequela	ICD10CM
T51.8X4	Toxic effect of other alcohols, undetermined	ICD10CM
T51.8X4A	Toxic effect of other alcohols, undetermined, initial encounter	ICD10CM
T51.8X4D	Toxic effect of other alcohols, undetermined, subsequent encounter	ICD10CM
T51.8X4S	Toxic effect of other alcohols, undetermined, sequela	ICD10CM
T51.94	Toxic effect of unspecified alcohol, undetermined	ICD10CM
T51.94XA	Toxic effect of unspecified alcohol, undetermined, initial encounter	ICD10CM
T51.94XD	Toxic effect of unspecified alcohol, undetermined, subsequent encounter	ICD10CM
T51.94XS	Toxic effect of unspecified alcohol, undetermined, sequela	ICD10CM
T52.0X4	Toxic effect of petroleum products, undetermined	ICD10CM
T52.0X4A	Toxic effect of petroleum products, undetermined, initial encounter	ICD10CM
T52.0X4D	Toxic effect of petroleum products, undetermined, subsequent encounter	ICD10CM
T52.0X4S	Toxic effect of petroleum products, undetermined, sequela	ICD10CM
T52.1X4	Toxic effect of benzene, undetermined	ICD10CM
T52.1X4A	Toxic effect of benzene, undetermined, initial encounter	ICD10CM
T52.1X4D	Toxic effect of benzene, undetermined, subsequent encounter	ICD10CM
T52.1X4S	Toxic effect of benzene, undetermined, sequela	ICD10CM
T52.2X4	Toxic effect of homologues of benzene, undetermined	ICD10CM
T52.2X4A	Toxic effect of homologues of benzene, undetermined, initial encounter	ICD10CM
T52.2X4D	Toxic effect of homologues of benzene, undetermined, subsequent encounter	ICD10CM
T52.2X4S	Toxic effect of homologues of benzene, undetermined, sequela	ICD10CM

© 2021 Walsh CG et al. *JAMA Network Open.*

**T52.3X4**	Toxic effect of glycols, undetermined	ICD10CM
**T52.3X4A**	Toxic effect of glycols, undetermined, initial encounter	ICD10CM
**T52.3X4D**	Toxic effect of glycols, undetermined, subsequent encounter	ICD10CM
**T52.3X4S**	Toxic effect of glycols, undetermined, sequela	ICD10CM
**T52.4X4**	Toxic effect of ketones, undetermined	ICD10CM
**T52.4X4A**	Toxic effect of ketones, undetermined, initial encounter	ICD10CM
**T52.4X4D**	Toxic effect of ketones, undetermined, subsequent encounter	ICD10CM
**T52.4X4S**	Toxic effect of ketones, undetermined, sequela	ICD10CM
**T52.8X4**	Toxic effect of other organic solvents, undetermined	ICD10CM
**T52.8X4A**	Toxic effect of other organic solvents, undetermined, initial encounter	ICD10CM
**T52.8X4D**	Toxic effect of other organic solvents, undetermined, subsequent encounter	ICD10CM
**T52.8X4S**	Toxic effect of other organic solvents, undetermined, sequela	ICD10CM
**T52.94**	Toxic effect of unspecified organic solvent, undetermined	ICD10CM
**T52.94XA**	Toxic effect of unspecified organic solvent, undetermined, initial encounter	ICD10CM
**T52.94XD**	Toxic effect of unspecified organic solvent, undetermined, subsequent encounter	ICD10CM
**T52.94XS**	Toxic effect of unspecified organic solvent, undetermined, sequela	ICD10CM
**T53.0X4**	Toxic effect of carbon tetrachloride, undetermined	ICD10CM
**T53.0X4A**	Toxic effect of carbon tetrachloride, undetermined, initial encounter	ICD10CM
**T53.0X4D**	Toxic effect of carbon tetrachloride, undetermined, subsequent encounter	ICD10CM
**T53.0X4S**	Toxic effect of carbon tetrachloride, undetermined, sequela	ICD10CM
**T53.1X4**	Toxic effect of chloroform, undetermined	ICD10CM
**T53.1X4A**	Toxic effect of chloroform, undetermined, initial encounter	ICD10CM

© 2021 Walsh CG et al. *JAMA Network Open.*

T53.1X4D	Toxic effect of chloroform, undetermined, subsequent encounter	ICD10CM
T53.1X4S	Toxic effect of chloroform, undetermined, sequela	ICD10CM
T53.2X4	Toxic effect of trichloroethylene, undetermined	ICD10CM
T53.2X4A	Toxic effect of trichloroethylene, undetermined, initial encounter	ICD10CM
T53.2X4D	Toxic effect of trichloroethylene, undetermined, subsequent encounter	ICD10CM
T53.2X4S	Toxic effect of trichloroethylene, undetermined, sequela	ICD10CM
T53.3X4	Toxic effect of tetrachloroethylene, undetermined	ICD10CM
T53.3X4A	Toxic effect of tetrachloroethylene, undetermined, initial encounter	ICD10CM
T53.3X4D	Toxic effect of tetrachloroethylene, undetermined, subsequent encounter	ICD10CM
T53.3X4S	Toxic effect of tetrachloroethylene, undetermined, sequela	ICD10CM
T53.4X4	Toxic effect of dichloromethane, undetermined	ICD10CM
T53.4X4A	Toxic effect of dichloromethane, undetermined, initial encounter	ICD10CM
T53.4X4D	Toxic effect of dichloromethane, undetermined, subsequent encounter	ICD10CM
T53.4X4S	Toxic effect of dichloromethane, undetermined, sequela	ICD10CM
T53.5X4	Toxic effect of chlorofluorocarbons, undetermined	ICD10CM
T53.5X4A	Toxic effect of chlorofluorocarbons, undetermined, initial encounter	ICD10CM
T53.5X4D	Toxic effect of chlorofluorocarbons, undetermined, subsequent encounter	ICD10CM
T53.5X4S	Toxic effect of chlorofluorocarbons, undetermined, sequela	ICD10CM
T53.6X4	Toxic effect of other halogen derivatives of aliphatic hydrocarbons, undetermined	ICD10CM
T53.6X4A	Toxic effect of other halogen derivatives of aliphatic hydrocarbons, undetermined, initial encounter	ICD10CM
T53.6X4D	Toxic effect of other halogen derivatives of aliphatic hydrocarbons, undetermined, subsequent encounter	ICD10CM

© 2021 Walsh CG et al. *JAMA Network Open.*

T53.6X4S	Toxic effect of other halogen derivatives of aliphatic hydrocarbons, undetermined, sequela	ICD10CM
T53.7X4	Toxic effect of other halogen derivatives of aromatic hydrocarbons, undetermined	ICD10CM
T53.7X4A	Toxic effect of other halogen derivatives of aromatic hydrocarbons, undetermined, initial encounter	ICD10CM
T53.7X4D	Toxic effect of other halogen derivatives of aromatic hydrocarbons, undetermined, subsequent encounter	ICD10CM
T53.7X4S	Toxic effect of other halogen derivatives of aromatic hydrocarbons, undetermined, sequela	ICD10CM
T53.94	Toxic effect of unspecified halogen derivatives of aliphatic and aromatic hydrocarbons, undetermined	ICD10CM
T53.94XA	Toxic effect of unspecified halogen derivatives of aliphatic and aromatic hydrocarbons, undetermined, initial encounter	ICD10CM
T53.94XD	Toxic effect of unspecified halogen derivatives of aliphatic and aromatic hydrocarbons, undetermined, subsequent encounter	ICD10CM
T53.94XS	Toxic effect of unspecified halogen derivatives of aliphatic and aromatic hydrocarbons, undetermined, sequela	ICD10CM
T54.0X4	Toxic effect of phenol and phenol homologues, undetermined	ICD10CM
T54.0X4A	Toxic effect of phenol and phenol homologues, undetermined, initial encounter	ICD10CM
T54.0X4D	Toxic effect of phenol and phenol homologues, undetermined, subsequent encounter	ICD10CM
T54.0X4S	Toxic effect of phenol and phenol homologues, undetermined, sequela	ICD10CM
T54.1X4	Toxic effect of other corrosive organic compounds, undetermined	ICD10CM
T54.1X4A	Toxic effect of other corrosive organic compounds, undetermined, initial encounter	ICD10CM
T54.1X4D	Toxic effect of other corrosive organic compounds, undetermined, subsequent encounter	ICD10CM
T54.1X4S	Toxic effect of other corrosive organic compounds, undetermined, sequela	ICD10CM
T54.2X4	Toxic effect of corrosive acids and acid-like substances, undetermined	ICD10CM
T54.2X4A	Toxic effect of corrosive acids and acid-like substances, undetermined, initial encounter	ICD10CM

© 2021 Walsh CG et al. *JAMA Network Open.*

T54.2X4D	Toxic effect of corrosive acids and acid-like substances, undetermined, subsequent encounter	ICD10CM
T54.2X4S	Toxic effect of corrosive acids and acid-like substances, undetermined, sequela	ICD10CM
T54.3X4	Toxic effect of corrosive alkalis and alkali-like substances, undetermined	ICD10CM
T54.3X4A	Toxic effect of corrosive alkalis and alkali-like substances, undetermined, initial encounter	ICD10CM
T54.3X4D	Toxic effect of corrosive alkalis and alkali-like substances, undetermined, subsequent encounter	ICD10CM
T54.3X4S	Toxic effect of corrosive alkalis and alkali-like substances, undetermined, sequela	ICD10CM
T54.94	Toxic effect of unspecified corrosive substance, undetermined	ICD10CM
T54.94XA	Toxic effect of unspecified corrosive substance, undetermined, initial encounter	ICD10CM
T54.94XD	Toxic effect of unspecified corrosive substance, undetermined, subsequent encounter	ICD10CM
T54.94XS	Toxic effect of unspecified corrosive substance, undetermined, sequela	ICD10CM
T55.0X4	Toxic effect of soaps, undetermined	ICD10CM
T55.0X4A	Toxic effect of soaps, undetermined, initial encounter	ICD10CM
T55.0X4D	Toxic effect of soaps, undetermined, subsequent encounter	ICD10CM
T55.0X4S	Toxic effect of soaps, undetermined, sequela	ICD10CM
T55.1X4	Toxic effect of detergents, undetermined	ICD10CM
T55.1X4A	Toxic effect of detergents, undetermined, initial encounter	ICD10CM
T55.1X4D	Toxic effect of detergents, undetermined, subsequent encounter	ICD10CM
T55.1X4S	Toxic effect of detergents, undetermined, sequela	ICD10CM
T56.0X4	Toxic effect of lead and its compounds, undetermined	ICD10CM
T56.0X4A	Toxic effect of lead and its compounds, undetermined, initial encounter	ICD10CM
T56.0X4D	Toxic effect of lead and its compounds, undetermined, subsequent encounter	ICD10CM

© 2021 Walsh CG et al. *JAMA Network Open.*

T56.0X4S	Toxic effect of lead and its compounds, undetermined, sequela	ICD10CM
T56.1X4	Toxic effect of mercury and its compounds, undetermined	ICD10CM
T56.1X4A	Toxic effect of mercury and its compounds, undetermined, initial encounter	ICD10CM
T56.1X4D	Toxic effect of mercury and its compounds, undetermined, subsequent encounter	ICD10CM
T56.1X4S	Toxic effect of mercury and its compounds, undetermined, sequela	ICD10CM
T56.2X4	Toxic effect of chromium and its compounds, undetermined	ICD10CM
T56.2X4A	Toxic effect of chromium and its compounds, undetermined, initial encounter	ICD10CM
T56.2X4D	Toxic effect of chromium and its compounds, undetermined, subsequent encounter	ICD10CM
T56.2X4S	Toxic effect of chromium and its compounds, undetermined, sequela	ICD10CM
T56.3X4	Toxic effect of cadmium and its compounds, undetermined	ICD10CM
T56.3X4A	Toxic effect of cadmium and its compounds, undetermined, initial encounter	ICD10CM
T56.3X4D	Toxic effect of cadmium and its compounds, undetermined, subsequent encounter	ICD10CM
T56.3X4S	Toxic effect of cadmium and its compounds, undetermined, sequela	ICD10CM
T56.4X4	Toxic effect of copper and its compounds, undetermined	ICD10CM
T56.4X4A	Toxic effect of copper and its compounds, undetermined, initial encounter	ICD10CM
T56.4X4D	Toxic effect of copper and its compounds, undetermined, subsequent encounter	ICD10CM
T56.4X4S	Toxic effect of copper and its compounds, undetermined, sequela	ICD10CM
T56.5X4	Toxic effect of zinc and its compounds, undetermined	ICD10CM
T56.5X4A	Toxic effect of zinc and its compounds, undetermined, initial encounter	ICD10CM
T56.5X4D	Toxic effect of zinc and its compounds, undetermined, subsequent encounter	ICD10CM
T56.5X4S	Toxic effect of zinc and its compounds, undetermined, sequela	ICD10CM
T56.6X4	Toxic effect of tin and its compounds, undetermined	ICD10CM

© 2021 Walsh CG et al. *JAMA Network Open.*

**T56.6X4A**	Toxic effect of tin and its compounds, undetermined, initial encounter	ICD10CM
**T56.6X4D**	Toxic effect of tin and its compounds, undetermined, subsequent encounter	ICD10CM
**T56.6X4S**	Toxic effect of tin and its compounds, undetermined, sequela	ICD10CM
**T56.7X4**	Toxic effect of beryllium and its compounds, undetermined	ICD10CM
**T56.7X4A**	Toxic effect of beryllium and its compounds, undetermined, initial encounter	ICD10CM
**T56.7X4D**	Toxic effect of beryllium and its compounds, undetermined, subsequent encounter	ICD10CM
**T56.7X4S**	Toxic effect of beryllium and its compounds, undetermined, sequela	ICD10CM
**T56.814**	Toxic effect of thallium, undetermined	ICD10CM
**T56.814A**	Toxic effect of thallium, undetermined, initial encounter	ICD10CM
**T56.814D**	Toxic effect of thallium, undetermined, subsequent encounter	ICD10CM
**T56.814S**	Toxic effect of thallium, undetermined, sequela	ICD10CM
**T56.894**	Toxic effect of other metals, undetermined	ICD10CM
**T56.894A**	Toxic effect of other metals, undetermined, initial encounter	ICD10CM
**T56.894D**	Toxic effect of other metals, undetermined, subsequent encounter	ICD10CM
**T56.894S**	Toxic effect of other metals, undetermined, sequela	ICD10CM
**T56.8x4A**	Toxic effect of other metals, undetermined, initial encounter	ICD10CM
**T56.8x4D**	Toxic effect of other metals, undetermined, subsequent encounter	ICD10CM
**T56.8x4S**	Toxic effect of other metals, undetermined, sequela	ICD10CM
**T56.94**	Toxic effect of unspecified metal, undetermined	ICD10CM
**T56.94XA**	Toxic effect of unspecified metal, undetermined, initial encounter	ICD10CM
**T56.94XD**	Toxic effect of unspecified metal, undetermined, subsequent encounter	ICD10CM
**T56.94XS**	Toxic effect of unspecified metal, undetermined, sequela	ICD10CM

© 2021 Walsh CG et al. *JAMA Network Open.*

T57.0X4	Toxic effect of arsenic and its compounds, undetermined	ICD10CM
T57.0X4A	Toxic effect of arsenic and its compounds, undetermined, initial encounter	ICD10CM
T57.0X4D	Toxic effect of arsenic and its compounds, undetermined, subsequent encounter	ICD10CM
T57.0X4S	Toxic effect of arsenic and its compounds, undetermined, sequela	ICD10CM
T57.1X4	Toxic effect of phosphorus and its compounds, undetermined	ICD10CM
T57.1X4A	Toxic effect of phosphorus and its compounds, undetermined, initial encounter	ICD10CM
T57.1X4D	Toxic effect of phosphorus and its compounds, undetermined, subsequent encounter	ICD10CM
T57.1X4S	Toxic effect of phosphorus and its compounds, undetermined, sequela	ICD10CM
T57.2X4	Toxic effect of manganese and its compounds, undetermined	ICD10CM
T57.2X4A	Toxic effect of manganese and its compounds, undetermined, initial encounter	ICD10CM
T57.2X4D	Toxic effect of manganese and its compounds, undetermined, subsequent encounter	ICD10CM
T57.2X4S	Toxic effect of manganese and its compounds, undetermined, sequela	ICD10CM
T57.3X4	Toxic effect of hydrogen cyanide, undetermined	ICD10CM
T57.3X4A	Toxic effect of hydrogen cyanide, undetermined, initial encounter	ICD10CM
T57.3X4D	Toxic effect of hydrogen cyanide, undetermined, subsequent encounter	ICD10CM
T57.3X4S	Toxic effect of hydrogen cyanide, undetermined, sequela	ICD10CM
T57.8X4	Toxic effect of other specified inorganic substances, undetermined	ICD10CM
T57.8X4A	Toxic effect of other specified inorganic substances, undetermined, initial encounter	ICD10CM
T57.8X4D	Toxic effect of other specified inorganic substances, undetermined, subsequent encounter	ICD10CM
T57.8X4S	Toxic effect of other specified inorganic substances, undetermined, sequela	ICD10CM
T57.94	Toxic effect of unspecified inorganic substance, undetermined	ICD10CM
T57.94XA	Toxic effect of unspecified inorganic substance, undetermined, initial encounter	ICD10CM

**T57.94XD**	Toxic effect of unspecified inorganic substance, undetermined, subsequent encounter	ICD10CM
**T57.94XS**	Toxic effect of unspecified inorganic substance, undetermined, sequela	ICD10CM
**T58.04**	Toxic effect of carbon monoxide from motor vehicle exhaust, undetermined	ICD10CM
**T58.04XA**	Toxic effect of carbon monoxide from motor vehicle exhaust, undetermined, initial encounter	ICD10CM
**T58.04XD**	Toxic effect of carbon monoxide from motor vehicle exhaust, undetermined, subsequent encounter	ICD10CM
**T58.04XS**	Toxic effect of carbon monoxide from motor vehicle exhaust, undetermined, sequela	ICD10CM
**T58.14**	Toxic effect of carbon monoxide from utility gas, undetermined	ICD10CM
**T58.14XA**	Toxic effect of carbon monoxide from utility gas, undetermined, initial encounter	ICD10CM
**T58.14XD**	Toxic effect of carbon monoxide from utility gas, undetermined, subsequent encounter	ICD10CM
**T58.14XS**	Toxic effect of carbon monoxide from utility gas, undetermined, sequela	ICD10CM
**T58.2X4**	Toxic effect of carbon monoxide from incomplete combustion of other domestic fuels, undetermined	ICD10CM
**T58.2X4A**	Toxic effect of carbon monoxide from incomplete combustion of other domestic fuels, undetermined, initial encounter	ICD10CM
**T58.2X4D**	Toxic effect of carbon monoxide from incomplete combustion of other domestic fuels, undetermined, subsequent encounter	ICD10CM
**T58.2X4S**	Toxic effect of carbon monoxide from incomplete combustion of other domestic fuels, undetermined, sequela	ICD10CM
**T58.8X4**	Toxic effect of carbon monoxide from other source, undetermined	ICD10CM
**T58.8X4A**	Toxic effect of carbon monoxide from other source, undetermined, initial encounter	ICD10CM
**T58.8X4D**	Toxic effect of carbon monoxide from other source, undetermined, subsequent encounter	ICD10CM
**T58.8X4S**	Toxic effect of carbon monoxide from other source, undetermined, sequela	ICD10CM
**T58.94**	Toxic effect of carbon monoxide from unspecified source, undetermined	ICD10CM

© 2021 Walsh CG et al. *JAMA Network Open.*

T58.94XA	Toxic effect of carbon monoxide from unspecified source, undetermined, initial encounter	ICD10CM
T58.94XD	Toxic effect of carbon monoxide from unspecified source, undetermined, subsequent encounter	ICD10CM
T58.94XS	Toxic effect of carbon monoxide from unspecified source, undetermined, sequela	ICD10CM
T59.0X4	Toxic effect of nitrogen oxides, undetermined	ICD10CM
T59.0X4A	Toxic effect of nitrogen oxides, undetermined, initial encounter	ICD10CM
T59.0X4D	Toxic effect of nitrogen oxides, undetermined, subsequent encounter	ICD10CM
T59.0X4S	Toxic effect of nitrogen oxides, undetermined, sequela	ICD10CM
T59.1X4	Toxic effect of sulfur dioxide, undetermined	ICD10CM
T59.1X4A	Toxic effect of sulfur dioxide, undetermined, initial encounter	ICD10CM
T59.1X4D	Toxic effect of sulfur dioxide, undetermined, subsequent encounter	ICD10CM
T59.1X4S	Toxic effect of sulfur dioxide, undetermined, sequela	ICD10CM
T59.2X4	Toxic effect of formaldehyde, undetermined	ICD10CM
T59.2X4A	Toxic effect of formaldehyde, undetermined, initial encounter	ICD10CM
T59.2X4D	Toxic effect of formaldehyde, undetermined, subsequent encounter	ICD10CM
T59.2X4S	Toxic effect of formaldehyde, undetermined, sequela	ICD10CM
T59.3X4	Toxic effect of lacrimogenic gas, undetermined	ICD10CM
T59.3X4A	Toxic effect of lacrimogenic gas, undetermined, initial encounter	ICD10CM
T59.3X4D	Toxic effect of lacrimogenic gas, undetermined, subsequent encounter	ICD10CM
T59.3X4S	Toxic effect of lacrimogenic gas, undetermined, sequela	ICD10CM
T59.4X4	Toxic effect of chlorine gas, undetermined	ICD10CM
T59.4X4A	Toxic effect of chlorine gas, undetermined, initial encounter	ICD10CM
T59.4X4D	Toxic effect of chlorine gas, undetermined, subsequent encounter	ICD10CM

© 2021 Walsh CG et al. *JAMA Network Open.*

T59.4X4S	Toxic effect of chlorine gas, undetermined, sequela	ICD10CM
T59.5X4	Toxic effect of fluorine gas and hydrogen fluoride, undetermined	ICD10CM
T59.5X4A	Toxic effect of fluorine gas and hydrogen fluoride, undetermined, initial encounter	ICD10CM
T59.5X4D	Toxic effect of fluorine gas and hydrogen fluoride, undetermined, subsequent encounter	ICD10CM
T59.5X4S	Toxic effect of fluorine gas and hydrogen fluoride, undetermined, sequela	ICD10CM
T59.6X4	Toxic effect of hydrogen sulfide, undetermined	ICD10CM
T59.6X4A	Toxic effect of hydrogen sulfide, undetermined, initial encounter	ICD10CM
T59.6X4D	Toxic effect of hydrogen sulfide, undetermined, subsequent encounter	ICD10CM
T59.6X4S	Toxic effect of hydrogen sulfide, undetermined, sequela	ICD10CM
T59.7X4	Toxic effect of carbon dioxide, undetermined	ICD10CM
T59.7X4A	Toxic effect of carbon dioxide, undetermined, initial encounter	ICD10CM
T59.7X4D	Toxic effect of carbon dioxide, undetermined, subsequent encounter	ICD10CM
T59.7X4S	Toxic effect of carbon dioxide, undetermined, sequela	ICD10CM
T59.814	Toxic effect of smoke, undetermined	ICD10CM
T59.814A	Toxic effect of smoke, undetermined, initial encounter	ICD10CM
T59.814D	Toxic effect of smoke, undetermined, subsequent encounter	ICD10CM
T59.814S	Toxic effect of smoke, undetermined, sequela	ICD10CM
T59.894	Toxic effect of other specified gases, fumes and vapors, undetermined	ICD10CM
T59.894A	Toxic effect of other specified gases, fumes and vapors, undetermined, initial encounter	ICD10CM
T59.894D	Toxic effect of other specified gases, fumes and vapors, undetermined, subsequent encounter	ICD10CM
T59.894S	Toxic effect of other specified gases, fumes and vapors, undetermined, sequela	ICD10CM

© 2021 Walsh CG et al. *JAMA Network Open.*

**T59.94**	Toxic effect of unspecified gases, fumes and vapors, undetermined	ICD10CM
**T59.94XA**	Toxic effect of unspecified gases, fumes and vapors, undetermined, initial encounter	ICD10CM
**T59.94XD**	Toxic effect of unspecified gases, fumes and vapors, undetermined, subsequent encounter	ICD10CM
**T59.94XS**	Toxic effect of unspecified gases, fumes and vapors, undetermined, sequela	ICD10CM
**T60.0X4**	Toxic effect of organophosphate and carbamate insecticides, undetermined	ICD10CM
**T60.0X4A**	Toxic effect of organophosphate and carbamate insecticides, undetermined, initial encounter	ICD10CM
**T60.0X4D**	Toxic effect of organophosphate and carbamate insecticides, undetermined, subsequent encounter	ICD10CM
**T60.0X4S**	Toxic effect of organophosphate and carbamate insecticides, undetermined, sequela	ICD10CM
**T60.1X4**	Toxic effect of halogenated insecticides, undetermined	ICD10CM
**T60.1X4A**	Toxic effect of halogenated insecticides, undetermined, initial encounter	ICD10CM
**T60.1X4D**	Toxic effect of halogenated insecticides, undetermined, subsequent encounter	ICD10CM
**T60.1X4S**	Toxic effect of halogenated insecticides, undetermined, sequela	ICD10CM
**T60.2X4**	Toxic effect of other insecticides, undetermined	ICD10CM
**T60.2X4A**	Toxic effect of other insecticides, undetermined, initial encounter	ICD10CM
**T60.2X4D**	Toxic effect of other insecticides, undetermined, subsequent encounter	ICD10CM
**T60.2X4S**	Toxic effect of other insecticides, undetermined, sequela	ICD10CM
**T60.3X4**	Toxic effect of herbicides and fungicides, undetermined	ICD10CM
**T60.3X4A**	Toxic effect of herbicides and fungicides, undetermined, initial encounter	ICD10CM
**T60.3X4D**	Toxic effect of herbicides and fungicides, undetermined, subsequent encounter	ICD10CM
**T60.3X4S**	Toxic effect of herbicides and fungicides, undetermined, sequela	ICD10CM
**T60.4X4**	Toxic effect of rodenticides, undetermined	ICD10CM

© 2021 Walsh CG et al. *JAMA Network Open.*

T60.4X4A	Toxic effect of rodenticides, undetermined, initial encounter	ICD10CM
T60.4X4D	Toxic effect of rodenticides, undetermined, subsequent encounter	ICD10CM
T60.4X4S	Toxic effect of rodenticides, undetermined, sequela	ICD10CM
T60.8X4	Toxic effect of other pesticides, undetermined	ICD10CM
T60.8X4A	Toxic effect of other pesticides, undetermined, initial encounter	ICD10CM
T60.8X4D	Toxic effect of other pesticides, undetermined, subsequent encounter	ICD10CM
T60.8X4S	Toxic effect of other pesticides, undetermined, sequela	ICD10CM
T60.94	Toxic effect of unspecified pesticide, undetermined	ICD10CM
T60.94XA	Toxic effect of unspecified pesticide, undetermined, initial encounter	ICD10CM
T60.94XD	Toxic effect of unspecified pesticide, undetermined, subsequent encounter	ICD10CM
T60.94XS	Toxic effect of unspecified pesticide, undetermined, sequela	ICD10CM
T61.04	Ciguatera fish poisoning, undetermined	ICD10CM
T61.04XA	Ciguatera fish poisoning, undetermined, initial encounter	ICD10CM
T61.04XD	Ciguatera fish poisoning, undetermined, subsequent encounter	ICD10CM
T61.04XS	Ciguatera fish poisoning, undetermined, sequela	ICD10CM
T61.14	Scombroid fish poisoning, undetermined	ICD10CM
T61.14XA	Scombroid fish poisoning, undetermined, initial encounter	ICD10CM
T61.14XD	Scombroid fish poisoning, undetermined, subsequent encounter	ICD10CM
T61.14XS	Scombroid fish poisoning, undetermined, sequela	ICD10CM
T61.774	Other fish poisoning, undetermined	ICD10CM
T61.774A	Other fish poisoning, undetermined, initial encounter	ICD10CM
T61.774D	Other fish poisoning, undetermined, subsequent encounter	ICD10CM

© 2021 Walsh CG et al. *JAMA Network Open.*

**T61.774S**	Other fish poisoning, undetermined, sequela	ICD10CM
**T61.784**	Other shellfish poisoning, undetermined	ICD10CM
**T61.784A**	Other shellfish poisoning, undetermined, initial encounter	ICD10CM
**T61.784D**	Other shellfish poisoning, undetermined, subsequent encounter	ICD10CM
**T61.784S**	Other shellfish poisoning, undetermined, sequela	ICD10CM
**T61.8X4**	Toxic effect of other seafood, undetermined	ICD10CM
**T61.8X4A**	Toxic effect of other seafood, undetermined, initial encounter	ICD10CM
**T61.8X4D**	Toxic effect of other seafood, undetermined, subsequent encounter	ICD10CM
**T61.8X4S**	Toxic effect of other seafood, undetermined, sequela	ICD10CM
**T61.94**	Toxic effect of unspecified seafood, undetermined	ICD10CM
**T61.94XA**	Toxic effect of unspecified seafood, undetermined, initial encounter	ICD10CM
**T61.94XD**	Toxic effect of unspecified seafood, undetermined, subsequent encounter	ICD10CM
**T61.94XS**	Toxic effect of unspecified seafood, undetermined, sequela	ICD10CM
**T62.0X4**	Toxic effect of ingested mushrooms, undetermined	ICD10CM
**T62.0X4A**	Toxic effect of ingested mushrooms, undetermined, initial encounter	ICD10CM
**T62.0X4D**	Toxic effect of ingested mushrooms, undetermined, subsequent encounter	ICD10CM
**T62.0X4S**	Toxic effect of ingested mushrooms, undetermined, sequela	ICD10CM
**T62.1X4**	Toxic effect of ingested berries, undetermined	ICD10CM
**T62.1X4A**	Toxic effect of ingested berries, undetermined, initial encounter	ICD10CM
**T62.1X4D**	Toxic effect of ingested berries, undetermined, subsequent encounter	ICD10CM
**T62.1X4S**	Toxic effect of ingested berries, undetermined, sequela	ICD10CM
**T62.2X4**	Toxic effect of other ingested (parts of) plant(s), undetermined	ICD10CM

© 2021 Walsh CG et al. *JAMA Network Open.*

**T62.2X4A**	Toxic effect of other ingested (parts of) plant(s), undetermined, initial encounter	ICD10CM
**T62.2X4D**	Toxic effect of other ingested (parts of) plant(s), undetermined, subsequent encounter	ICD10CM
**T62.2X4S**	Toxic effect of other ingested (parts of) plant(s), undetermined, sequela	ICD10CM
**T62.8X4**	Toxic effect of other specified noxious substances eaten as food, undetermined	ICD10CM
**T62.8X4A**	Toxic effect of other specified noxious substances eaten as food, undetermined, initial encounter	ICD10CM
**T62.8X4D**	Toxic effect of other specified noxious substances eaten as food, undetermined, subsequent encounter	ICD10CM
**T62.8X4S**	Toxic effect of other specified noxious substances eaten as food, undetermined, sequela	ICD10CM
**T62.94**	Toxic effect of unspecified noxious substance eaten as food, undetermined	ICD10CM
**T62.94XA**	Toxic effect of unspecified noxious substance eaten as food, undetermined, initial encounter	ICD10CM
**T62.94XD**	Toxic effect of unspecified noxious substance eaten as food, undetermined, subsequent encounter	ICD10CM
**T62.94XS**	Toxic effect of unspecified noxious substance eaten as food, undetermined, sequela	ICD10CM
**T63.004**	Toxic effect of unspecified snake venom, undetermined	ICD10CM
**T63.004A**	Toxic effect of unspecified snake venom, undetermined, initial encounter	ICD10CM
**T63.004D**	Toxic effect of unspecified snake venom, undetermined, subsequent encounter	ICD10CM
**T63.004S**	Toxic effect of unspecified snake venom, undetermined, sequela	ICD10CM
**T63.014**	Toxic effect of rattlesnake venom, undetermined	ICD10CM
**T63.014A**	Toxic effect of rattlesnake venom, undetermined, initial encounter	ICD10CM
**T63.014D**	Toxic effect of rattlesnake venom, undetermined, subsequent encounter	ICD10CM
**T63.014S**	Toxic effect of rattlesnake venom, undetermined, sequela	ICD10CM
**T63.024**	Toxic effect of coral snake venom, undetermined	ICD10CM

© 2021 Walsh CG et al. *JAMA Network Open.*

**T63.024A**	Toxic effect of coral snake venom, undetermined, initial encounter	ICD10CM
**T63.024D**	Toxic effect of coral snake venom, undetermined, subsequent encounter	ICD10CM
**T63.024S**	Toxic effect of coral snake venom, undetermined, sequela	ICD10CM
**T63.034**	Toxic effect of taipan venom, undetermined	ICD10CM
**T63.034A**	Toxic effect of taipan venom, undetermined, initial encounter	ICD10CM
**T63.034D**	Toxic effect of taipan venom, undetermined, subsequent encounter	ICD10CM
**T63.034S**	Toxic effect of taipan venom, undetermined, sequela	ICD10CM
**T63.044**	Toxic effect of cobra venom, undetermined	ICD10CM
**T63.044A**	Toxic effect of cobra venom, undetermined, initial encounter	ICD10CM
**T63.044D**	Toxic effect of cobra venom, undetermined, subsequent encounter	ICD10CM
**T63.044S**	Toxic effect of cobra venom, undetermined, sequela	ICD10CM
**T63.064**	Toxic effect of venom of other North and South American snake, undetermined	ICD10CM
**T63.064A**	Toxic effect of venom of other North and South American snake, undetermined, initial encounter	ICD10CM
**T63.064D**	Toxic effect of venom of other North and South American snake, undetermined, subsequent encounter	ICD10CM
**T63.064S**	Toxic effect of venom of other North and South American snake, undetermined, sequela	ICD10CM
**T63.074**	Toxic effect of venom of other Australian snake, undetermined	ICD10CM
**T63.074A**	Toxic effect of venom of other Australian snake, undetermined, initial encounter	ICD10CM
**T63.074D**	Toxic effect of venom of other Australian snake, undetermined, subsequent encounter	ICD10CM
**T63.074S**	Toxic effect of venom of other Australian snake, undetermined, sequela	ICD10CM
**T63.084**	Toxic effect of venom of other African and Asian snake, undetermined	ICD10CM
**T63.084A**	Toxic effect of venom of other African and Asian snake, undetermined, initial encounter	ICD10CM

© 2021 Walsh CG et al. *JAMA Network Open.*

T63.084D	Toxic effect of venom of other African and Asian snake, undetermined, subsequent encounter	ICD10CM
T63.084S	Toxic effect of venom of other African and Asian snake, undetermined, sequela	ICD10CM
T63.094	Toxic effect of venom of other snake, undetermined	ICD10CM
T63.094A	Toxic effect of venom of other snake, undetermined, initial encounter	ICD10CM
T63.094D	Toxic effect of venom of other snake, undetermined, subsequent encounter	ICD10CM
T63.094S	Toxic effect of venom of other snake, undetermined, sequela	ICD10CM
T63.114	Toxic effect of venom of gila monster, undetermined	ICD10CM
T63.114A	Toxic effect of venom of gila monster, undetermined, initial encounter	ICD10CM
T63.114D	Toxic effect of venom of gila monster, undetermined, subsequent encounter	ICD10CM
T63.114S	Toxic effect of venom of gila monster, undetermined, sequela	ICD10CM
T63.124	Toxic effect of venom of other venomous lizard, undetermined	ICD10CM
T63.124A	Toxic effect of venom of other venomous lizard, undetermined, initial encounter	ICD10CM
T63.124D	Toxic effect of venom of other venomous lizard, undetermined, subsequent encounter	ICD10CM
T63.124S	Toxic effect of venom of other venomous lizard, undetermined, sequela	ICD10CM
T63.194	Toxic effect of venom of other reptiles, undetermined	ICD10CM
T63.194A	Toxic effect of venom of other reptiles, undetermined, initial encounter	ICD10CM
T63.194D	Toxic effect of venom of other reptiles, undetermined, subsequent encounter	ICD10CM
T63.194S	Toxic effect of venom of other reptiles, undetermined, sequela	ICD10CM
T63.2X4	Toxic effect of venom of scorpion, undetermined	ICD10CM
T63.2X4A	Toxic effect of venom of scorpion, undetermined, initial encounter	ICD10CM
T63.2X4D	Toxic effect of venom of scorpion, undetermined, subsequent encounter	ICD10CM
T63.2X4S	Toxic effect of venom of scorpion, undetermined, sequela	ICD10CM

© 2021 Walsh CG et al. *JAMA Network Open.*

T63.304	Toxic effect of unspecified spider venom, undetermined	ICD10CM
T63.304A	Toxic effect of unspecified spider venom, undetermined, initial encounter	ICD10CM
T63.304D	Toxic effect of unspecified spider venom, undetermined, subsequent encounter	ICD10CM
T63.304S	Toxic effect of unspecified spider venom, undetermined, sequela	ICD10CM
T63.314	Toxic effect of venom of black widow spider, undetermined	ICD10CM
T63.314A	Toxic effect of venom of black widow spider, undetermined, initial encounter	ICD10CM
T63.314D	Toxic effect of venom of black widow spider, undetermined, subsequent encounter	ICD10CM
T63.314S	Toxic effect of venom of black widow spider, undetermined, sequela	ICD10CM
T63.324	Toxic effect of venom of tarantula, undetermined	ICD10CM
T63.324A	Toxic effect of venom of tarantula, undetermined, initial encounter	ICD10CM
T63.324D	Toxic effect of venom of tarantula, undetermined, subsequent encounter	ICD10CM
T63.324S	Toxic effect of venom of tarantula, undetermined, sequela	ICD10CM
T63.334	Toxic effect of venom of brown recluse spider, undetermined	ICD10CM
T63.334A	Toxic effect of venom of brown recluse spider, undetermined, initial encounter	ICD10CM
T63.334D	Toxic effect of venom of brown recluse spider, undetermined, subsequent encounter	ICD10CM
T63.334S	Toxic effect of venom of brown recluse spider, undetermined, sequela	ICD10CM
T63.394	Toxic effect of venom of other spider, undetermined	ICD10CM
T63.394A	Toxic effect of venom of other spider, undetermined, initial encounter	ICD10CM
T63.394D	Toxic effect of venom of other spider, undetermined, subsequent encounter	ICD10CM
T63.394S	Toxic effect of venom of other spider, undetermined, sequela	ICD10CM
T63.414	Toxic effect of venom of centipedes and venomous millipedes, undetermined	ICD10CM
T63.414A	Toxic effect of venom of centipedes and venomous millipedes, undetermined, initial encounter	ICD10CM

© 2021 Walsh CG et al. *JAMA Network Open.*

T63.414D	Toxic effect of venom of centipedes and venomous millipedes, undetermined, subsequent encounter	ICD10CM
T63.414S	Toxic effect of venom of centipedes and venomous millipedes, undetermined, sequela	ICD10CM
T63.424	Toxic effect of venom of ants, undetermined	ICD10CM
T63.424A	Toxic effect of venom of ants, undetermined, initial encounter	ICD10CM
T63.424D	Toxic effect of venom of ants, undetermined, subsequent encounter	ICD10CM
T63.424S	Toxic effect of venom of ants, undetermined, sequela	ICD10CM
T63.434	Toxic effect of venom of caterpillars, undetermined	ICD10CM
T63.434A	Toxic effect of venom of caterpillars, undetermined, initial encounter	ICD10CM
T63.434D	Toxic effect of venom of caterpillars, undetermined, subsequent encounter	ICD10CM
T63.434S	Toxic effect of venom of caterpillars, undetermined, sequela	ICD10CM
T63.444	Toxic effect of venom of bees, undetermined	ICD10CM
T63.444A	Toxic effect of venom of bees, undetermined, initial encounter	ICD10CM
T63.444D	Toxic effect of venom of bees, undetermined, subsequent encounter	ICD10CM
T63.444S	Toxic effect of venom of bees, undetermined, sequela	ICD10CM
T63.454	Toxic effect of venom of hornets, undetermined	ICD10CM
T63.454A	Toxic effect of venom of hornets, undetermined, initial encounter	ICD10CM
T63.454D	Toxic effect of venom of hornets, undetermined, subsequent encounter	ICD10CM
T63.454S	Toxic effect of venom of hornets, undetermined, sequela	ICD10CM
T63.464	Toxic effect of venom of wasps, undetermined	ICD10CM
T63.464A	Toxic effect of venom of wasps, undetermined, initial encounter	ICD10CM
T63.464D	Toxic effect of venom of wasps, undetermined, subsequent encounter	ICD10CM
T63.464S	Toxic effect of venom of wasps, undetermined, sequela	ICD10CM

© 2021 Walsh CG et al. *JAMA Network Open.*

T63.484	Toxic effect of venom of other arthropod, undetermined	ICD10CM
T63.484A	Toxic effect of venom of other arthropod, undetermined, initial encounter	ICD10CM
T63.484D	Toxic effect of venom of other arthropod, undetermined, subsequent encounter	ICD10CM
T63.484S	Toxic effect of venom of other arthropod, undetermined, sequela	ICD10CM
T63.514	Toxic effect of contact with stingray, undetermined	ICD10CM
T63.514A	Toxic effect of contact with stingray, undetermined, initial encounter	ICD10CM
T63.514D	Toxic effect of contact with stingray, undetermined, subsequent encounter	ICD10CM
T63.514S	Toxic effect of contact with stingray, undetermined, sequela	ICD10CM
T63.594	Toxic effect of contact with other venomous fish, undetermined	ICD10CM
T63.594A	Toxic effect of contact with other venomous fish, undetermined, initial encounter	ICD10CM
T63.594D	Toxic effect of contact with other venomous fish, undetermined, subsequent encounter	ICD10CM
T63.594S	Toxic effect of contact with other venomous fish, undetermined, sequela	ICD10CM
T63.614	Toxic effect of contact with Portugese Man-o-war, undetermined	ICD10CM
T63.614A	Toxic effect of contact with Portugese Man-o-war, undetermined, initial encounter	ICD10CM
T63.614D	Toxic effect of contact with Portugese Man-o-war, undetermined, subsequent encounter	ICD10CM
T63.614S	Toxic effect of contact with Portugese Man-o-war, undetermined, sequela	ICD10CM
T63.624	Toxic effect of contact with other jellyfish, undetermined	ICD10CM
T63.624A	Toxic effect of contact with other jellyfish, undetermined, initial encounter	ICD10CM
T63.624D	Toxic effect of contact with other jellyfish, undetermined, subsequent encounter	ICD10CM
T63.624S	Toxic effect of contact with other jellyfish, undetermined, sequela	ICD10CM
T63.634	Toxic effect of contact with sea anemone, undetermined	ICD10CM
T63.634A	Toxic effect of contact with sea anemone, undetermined, initial encounter	ICD10CM

© 2021 Walsh CG et al. *JAMA Network Open.*

T63.634D	Toxic effect of contact with sea anemone, undetermined, subsequent encounter	ICD10CM
T63.634S	Toxic effect of contact with sea anemone, undetermined, sequela	ICD10CM
T63.694	Toxic effect of contact with other venomous marine animals, undetermined	ICD10CM
T63.694A	Toxic effect of contact with other venomous marine animals, undetermined, initial encounter	ICD10CM
T63.694D	Toxic effect of contact with other venomous marine animals, undetermined, subsequent encounter	ICD10CM
T63.694S	Toxic effect of contact with other venomous marine animals, undetermined, sequela	ICD10CM
T63.714	Toxic effect of contact with venomous marine plant, undetermined	ICD10CM
T63.714A	Toxic effect of contact with venomous marine plant, undetermined, initial encounter	ICD10CM
T63.714D	Toxic effect of contact with venomous marine plant, undetermined, subsequent encounter	ICD10CM
T63.714S	Toxic effect of contact with venomous marine plant, undetermined, sequela	ICD10CM
T63.794	Toxic effect of contact with other venomous plant, undetermined	ICD10CM
T63.794A	Toxic effect of contact with other venomous plant, undetermined, initial encounter	ICD10CM
T63.794D	Toxic effect of contact with other venomous plant, undetermined, subsequent encounter	ICD10CM
T63.794S	Toxic effect of contact with other venomous plant, undetermined, sequela	ICD10CM
T63.814	Toxic effect of contact with venomous frog, undetermined	ICD10CM
T63.814A	Toxic effect of contact with venomous frog, undetermined, initial encounter	ICD10CM
T63.814D	Toxic effect of contact with venomous frog, undetermined, subsequent encounter	ICD10CM
T63.814S	Toxic effect of contact with venomous frog, undetermined, sequela	ICD10CM
T63.824	Toxic effect of contact with venomous toad, undetermined	ICD10CM
T63.824A	Toxic effect of contact with venomous toad, undetermined, initial encounter	ICD10CM
T63.824D	Toxic effect of contact with venomous toad, undetermined, subsequent encounter	ICD10CM

© 2021 Walsh CG et al. *JAMA Network Open.*

**T63.824S**	Toxic effect of contact with venomous toad, undetermined, sequela	ICD10CM
**T63.834**	Toxic effect of contact with other venomous amphibian, undetermined	ICD10CM
**T63.834A**	Toxic effect of contact with other venomous amphibian, undetermined, initial encounter	ICD10CM
**T63.834D**	Toxic effect of contact with other venomous amphibian, undetermined, subsequent encounter	ICD10CM
**T63.834S**	Toxic effect of contact with other venomous amphibian, undetermined, sequela	ICD10CM
**T63.894**	Toxic effect of contact with other venomous animals, undetermined	ICD10CM
**T63.894A**	Toxic effect of contact with other venomous animals, undetermined, initial encounter	ICD10CM
**T63.894D**	Toxic effect of contact with other venomous animals, undetermined, subsequent encounter	ICD10CM
**T63.894S**	Toxic effect of contact with other venomous animals, undetermined, sequela	ICD10CM
**T63.94**	Toxic effect of contact with unspecified venomous animal, undetermined	ICD10CM
**T63.94XA**	Toxic effect of contact with unspecified venomous animal, undetermined, initial encounter	ICD10CM
**T63.94XD**	Toxic effect of contact with unspecified venomous animal, undetermined, subsequent encounter	ICD10CM
**T63.94XS**	Toxic effect of contact with unspecified venomous animal, undetermined, sequela	ICD10CM
**T64.04**	Toxic effect of aflatoxin, undetermined	ICD10CM
**T64.04XA**	Toxic effect of aflatoxin, undetermined, initial encounter	ICD10CM
**T64.04XD**	Toxic effect of aflatoxin, undetermined, subsequent encounter	ICD10CM
**T64.04XS**	Toxic effect of aflatoxin, undetermined, sequela	ICD10CM
**T64.84**	Toxic effect of other mycotoxin food contaminants, undetermined	ICD10CM
**T64.84XA**	Toxic effect of other mycotoxin food contaminants, undetermined, initial encounter	ICD10CM
**T64.84XD**	Toxic effect of other mycotoxin food contaminants, undetermined, subsequent encounter	ICD10CM

© 2021 Walsh CG et al. *JAMA Network Open.*

T64.84XS	Toxic effect of other mycotoxin food contaminants, undetermined, sequela	ICD10CM
T65.0X4	Toxic effect of cyanides, undetermined	ICD10CM
T65.0X4A	Toxic effect of cyanides, undetermined, initial encounter	ICD10CM
T65.0X4D	Toxic effect of cyanides, undetermined, subsequent encounter	ICD10CM
T65.0X4S	Toxic effect of cyanides, undetermined, sequela	ICD10CM
T65.1X4	Toxic effect of strychnine and its salts, undetermined	ICD10CM
T65.1X4A	Toxic effect of strychnine and its salts, undetermined, initial encounter	ICD10CM
T65.1X4D	Toxic effect of strychnine and its salts, undetermined, subsequent encounter	ICD10CM
T65.1X4S	Toxic effect of strychnine and its salts, undetermined, sequela	ICD10CM
T65.214	Toxic effect of chewing tobacco, undetermined	ICD10CM
T65.214A	Toxic effect of chewing tobacco, undetermined, initial encounter	ICD10CM
T65.214D	Toxic effect of chewing tobacco, undetermined, subsequent encounter	ICD10CM
T65.214S	Toxic effect of chewing tobacco, undetermined, sequela	ICD10CM
T65.224	Toxic effect of tobacco cigarettes, undetermined	ICD10CM
T65.224A	Toxic effect of tobacco cigarettes, undetermined, initial encounter	ICD10CM
T65.224D	Toxic effect of tobacco cigarettes, undetermined, subsequent encounter	ICD10CM
T65.224S	Toxic effect of tobacco cigarettes, undetermined, sequela	ICD10CM
T65.294	Toxic effect of other tobacco and nicotine, undetermined	ICD10CM
T65.294A	Toxic effect of other tobacco and nicotine, undetermined, initial encounter	ICD10CM
T65.294D	Toxic effect of other tobacco and nicotine, undetermined, subsequent encounter	ICD10CM
T65.294S	Toxic effect of other tobacco and nicotine, undetermined, sequela	ICD10CM
T65.3X4	Toxic effect of nitroderivatives and aminoderivatives of benzene and its homologues, undetermined	ICD10CM

© 2021 Walsh CG et al. *JAMA Network Open.*

T65.3X4A	Toxic effect of nitroderivatives and aminoderivatives of benzene and its homologues, undetermined, initial encounter	ICD10CM
T65.3X4D	Toxic effect of nitroderivatives and aminoderivatives of benzene and its homologues, undetermined, subsequent encounter	ICD10CM
T65.3X4S	Toxic effect of nitroderivatives and aminoderivatives of benzene and its homologues, undetermined, sequela	ICD10CM
T65.4X4	Toxic effect of carbon disulfide, undetermined	ICD10CM
T65.4X4A	Toxic effect of carbon disulfide, undetermined, initial encounter	ICD10CM
T65.4X4D	Toxic effect of carbon disulfide, undetermined, subsequent encounter	ICD10CM
T65.4X4S	Toxic effect of carbon disulfide, undetermined, sequela	ICD10CM
T65.5X4	Toxic effect of nitroglycerin and other nitric acids and esters, undetermined	ICD10CM
T65.5X4A	Toxic effect of nitroglycerin and other nitric acids and esters, undetermined, initial encounter	ICD10CM
T65.5X4D	Toxic effect of nitroglycerin and other nitric acids and esters, undetermined, subsequent encounter	ICD10CM
T65.5X4S	Toxic effect of nitroglycerin and other nitric acids and esters, undetermined, sequela	ICD10CM
T65.6X4	Toxic effect of paints and dyes, not elsewhere classified, undetermined	ICD10CM
T65.6X4A	Toxic effect of paints and dyes, not elsewhere classified, undetermined, initial encounter	ICD10CM
T65.6X4D	Toxic effect of paints and dyes, not elsewhere classified, undetermined, subsequent encounter	ICD10CM
T65.6X4S	Toxic effect of paints and dyes, not elsewhere classified, undetermined, sequela	ICD10CM
T65.814	Toxic effect of latex, undetermined	ICD10CM
T65.814A	Toxic effect of latex, undetermined, initial encounter	ICD10CM
T65.814D	Toxic effect of latex, undetermined, subsequent encounter	ICD10CM
T65.814S	Toxic effect of latex, undetermined, sequela	ICD10CM
T65.824	Toxic effect of harmful algae and algae toxins, undetermined	ICD10CM

© 2021 Walsh CG et al. *JAMA Network Open.*

T65.824A	Toxic effect of harmful algae and algae toxins, undetermined, initial encounter	ICD10CM
T65.824D	Toxic effect of harmful algae and algae toxins, undetermined, subsequent encounter	ICD10CM
T65.824S	Toxic effect of harmful algae and algae toxins, undetermined, sequela	ICD10CM
T65.834	Toxic effect of fiberglass, undetermined	ICD10CM
T65.834A	Toxic effect of fiberglass, undetermined, initial encounter	ICD10CM
T65.834D	Toxic effect of fiberglass, undetermined, subsequent encounter	ICD10CM
T65.834S	Toxic effect of fiberglass, undetermined, sequela	ICD10CM
T65.894	Toxic effect of other specified substances, undetermined	ICD10CM
T65.894A	Toxic effect of other specified substances, undetermined, initial encounter	ICD10CM
T65.894D	Toxic effect of other specified substances, undetermined, subsequent encounter	ICD10CM
T65.894S	Toxic effect of other specified substances, undetermined, sequela	ICD10CM
T65.94	Toxic effect of unspecified substance, undetermined	ICD10CM
T65.94XA	Toxic effect of unspecified substance, undetermined, initial encounter	ICD10CM
T65.94XD	Toxic effect of unspecified substance, undetermined, subsequent encounter	ICD10CM
T65.94XS	Toxic effect of unspecified substance, undetermined, sequela	ICD10CM
T71.114	Asphyxiation due to smothering under pillow, undetermined	ICD10CM
T71.114A	Asphyxiation due to smothering under pillow, undetermined, initial encounter	ICD10CM
T71.114D	Asphyxiation due to smothering under pillow, undetermined, subsequent encounter	ICD10CM
T71.114S	Asphyxiation due to smothering under pillow, undetermined, sequela	ICD10CM
T71.124	Asphyxiation due to plastic bag, undetermined	ICD10CM
T71.124A	Asphyxiation due to plastic bag, undetermined, initial encounter	ICD10CM
T71.124D	Asphyxiation due to plastic bag, undetermined, subsequent encounter	ICD10CM

© 2021 Walsh CG et al. *JAMA Network Open.*

T71.124S	Asphyxiation due to plastic bag, undetermined, sequela	ICD10CM
T71.134	Asphyxiation due to being trapped in bed linens, undetermined	ICD10CM
T71.134A	Asphyxiation due to being trapped in bed linens, undetermined, initial encounter	ICD10CM
T71.134D	Asphyxiation due to being trapped in bed linens, undetermined, subsequent encounter	ICD10CM
T71.134S	Asphyxiation due to being trapped in bed linens, undetermined, sequela	ICD10CM
T71.144	Asphyxiation due to smothering under another person's body (in bed), undetermined	ICD10CM
T71.144A	Asphyxiation due to smothering under another person's body (in bed), undetermined, initial encounter	ICD10CM
T71.144D	Asphyxiation due to smothering under another person's body (in bed), undetermined, subsequent encounter	ICD10CM
T71.144S	Asphyxiation due to smothering under another person's body (in bed), undetermined, sequela	ICD10CM
T71.154	Asphyxiation due to smothering in furniture, undetermined	ICD10CM
T71.154A	Asphyxiation due to smothering in furniture, undetermined, initial encounter	ICD10CM
T71.154D	Asphyxiation due to smothering in furniture, undetermined, subsequent encounter	ICD10CM
T71.154S	Asphyxiation due to smothering in furniture, undetermined, sequela	ICD10CM
T71.164	Asphyxiation due to hanging, undetermined	ICD10CM
T71.164A	Asphyxiation due to hanging, undetermined, initial encounter	ICD10CM
T71.164D	Asphyxiation due to hanging, undetermined, subsequent encounter	ICD10CM
T71.164S	Asphyxiation due to hanging, undetermined, sequela	ICD10CM
T71.194	Asphyxiation due to mechanical threat to breathing due to other causes, undetermined	ICD10CM
T71.194A	Asphyxiation due to mechanical threat to breathing due to other causes, undetermined, initial encounter	ICD10CM
T71.194D	Asphyxiation due to mechanical threat to breathing due to other causes, undetermined, subsequent encounter	ICD10CM

© 2021 Walsh CG et al. *JAMA Network Open.*

T71.194S	Asphyxiation due to mechanical threat to breathing due to other causes, undetermined, sequela	ICD10CM
T71.224	Asphyxiation due to being trapped in a car trunk, undetermined	ICD10CM
T71.224A	Asphyxiation due to being trapped in a car trunk, undetermined, initial encounter	ICD10CM
T71.224D	Asphyxiation due to being trapped in a car trunk, undetermined, subsequent encounter	ICD10CM
T71.224S	Asphyxiation due to being trapped in a car trunk, undetermined, sequela	ICD10CM
T71.234	Asphyxiation due to being trapped in a (discarded) refrigerator, undetermined	ICD10CM
T71.234A	Asphyxiation due to being trapped in a (discarded) refrigerator, undetermined, initial encounter	ICD10CM
T71.234D	Asphyxiation due to being trapped in a (discarded) refrigerator, undetermined, subsequent encounter	ICD10CM
T71.234S	Asphyxiation due to being trapped in a (discarded) refrigerator, undetermined, sequela	ICD10CM
Y21	Drowning and submersion, undetermined intent	ICD10CM
Y21.0	Drowning and submersion while in bathtub, undetermined intent	ICD10CM
Y21.0XXA	Drowning and submersion while in bathtub, undetermined intent, initial encounter	ICD10CM
Y21.0XXD	Drowning and submersion while in bathtub, undetermined intent, subsequent encounter	ICD10CM
Y21.0XXS	Drowning and submersion while in bathtub, undetermined intent, sequela	ICD10CM
Y21.1	Drowning and submersion after fall into bathtub, undetermined intent	ICD10CM
Y21.1XXA	Drowning and submersion after fall into bathtub, undetermined intent, initial encounter	ICD10CM
Y21.1XXD	Drowning and submersion after fall into bathtub, undetermined intent, subsequent encounter	ICD10CM
Y21.1XXS	Drowning and submersion after fall into bathtub, undetermined intent, sequela	ICD10CM
Y21.2	Drowning and submersion while in swimming pool, undetermined intent	ICD10CM
Y21.2XXA	Drowning and submersion while in swimming pool, undetermined intent, initial encounter	ICD10CM

© 2021 Walsh CG et al. *JAMA Network Open.*

Y21.2XXD	Drowning and submersion while in swimming pool, undetermined intent, subsequent encounter	ICD10CM
Y21.2XXS	Drowning and submersion while in swimming pool, undetermined intent, sequela	ICD10CM
Y21.3	Drowning and submersion after fall into swimming pool, undetermined intent	ICD10CM
Y21.3XXA	Drowning and submersion after fall into swimming pool, undetermined intent, initial encounter	ICD10CM
Y21.3XXD	Drowning and submersion after fall into swimming pool, undetermined intent, subsequent encounter	ICD10CM
Y21.3XXS	Drowning and submersion after fall into swimming pool, undetermined intent, sequela	ICD10CM
Y21.4	Drowning and submersion in natural water, undetermined intent	ICD10CM
Y21.4XXA	Drowning and submersion in natural water, undetermined intent, initial encounter	ICD10CM
Y21.4XXD	Drowning and submersion in natural water, undetermined intent, subsequent encounter	ICD10CM
Y21.4XXS	Drowning and submersion in natural water, undetermined intent, sequela	ICD10CM
Y21.8	Other drowning and submersion, undetermined intent	ICD10CM
Y21.8XXA	Other drowning and submersion, undetermined intent, initial encounter	ICD10CM
Y21.8XXD	Other drowning and submersion, undetermined intent, subsequent encounter	ICD10CM
Y21.8XXS	Other drowning and submersion, undetermined intent, sequela	ICD10CM
Y21.9	Unspecified drowning and submersion, undetermined intent	ICD10CM
Y21.9XXA	Unspecified drowning and submersion, undetermined intent, initial encounter	ICD10CM
Y21.9XXD	Unspecified drowning and submersion, undetermined intent, subsequent encounter	ICD10CM
Y21.9XXS	Unspecified drowning and submersion, undetermined intent, sequela	ICD10CM
Y22	Handgun discharge, undetermined intent	ICD10CM
Y22.XXXA	Handgun discharge, undetermined intent, initial encounter	ICD10CM
Y22.XXXD	Handgun discharge, undetermined intent, subsequent encounter	ICD10CM

© 2021 Walsh CG et al. *JAMA Network Open.*

Y22.XXXS	Handgun discharge, undetermined intent, sequela	ICD10CM
Y23	Rifle, shotgun and larger firearm discharge, undetermined intent	ICD10CM
Y23.0	Shotgun discharge, undetermined intent	ICD10CM
Y23.0XXA	Shotgun discharge, undetermined intent, initial encounter	ICD10CM
Y23.0XXD	Shotgun discharge, undetermined intent, subsequent encounter	ICD10CM
Y23.0XXS	Shotgun discharge, undetermined intent, sequela	ICD10CM
Y23.1	Hunting rifle discharge, undetermined intent	ICD10CM
Y23.1XXA	Hunting rifle discharge, undetermined intent, initial encounter	ICD10CM
Y23.1XXD	Hunting rifle discharge, undetermined intent, subsequent encounter	ICD10CM
Y23.1XXS	Hunting rifle discharge, undetermined intent, sequela	ICD10CM
Y23.2	Military firearm discharge, undetermined intent	ICD10CM
Y23.2XXA	Military firearm discharge, undetermined intent, initial encounter	ICD10CM
Y23.2XXD	Military firearm discharge, undetermined intent, subsequent encounter	ICD10CM
Y23.2XXS	Military firearm discharge, undetermined intent, sequela	ICD10CM
Y23.3	Machine gun discharge, undetermined intent	ICD10CM
Y23.3XXA	Machine gun discharge, undetermined intent, initial encounter	ICD10CM
Y23.3XXD	Machine gun discharge, undetermined intent, subsequent encounter	ICD10CM
Y23.3XXS	Machine gun discharge, undetermined intent, sequela	ICD10CM
Y23.8	Other larger firearm discharge, undetermined intent	ICD10CM
Y23.8XXA	Other larger firearm discharge, undetermined intent, initial encounter	ICD10CM
Y23.8XXD	Other larger firearm discharge, undetermined intent, subsequent encounter	ICD10CM
Y23.8XXS	Other larger firearm discharge, undetermined intent, sequela	ICD10CM

© 2021 Walsh CG et al. *JAMA Network Open.*

Y23.9	Unspecified larger firearm discharge, undetermined intent	ICD10CM
Y23.9XXA	Unspecified larger firearm discharge, undetermined intent, initial encounter	ICD10CM
Y23.9XXD	Unspecified larger firearm discharge, undetermined intent, subsequent encounter	ICD10CM
Y23.9XXS	Unspecified larger firearm discharge, undetermined intent, sequela	ICD10CM
Y24	Other and unspecified firearm discharge, undetermined intent	ICD10CM
Y24.0	Airgun discharge, undetermined intent	ICD10CM
Y24.0XXA	Airgun discharge, undetermined intent, initial encounter	ICD10CM
Y24.0XXD	Airgun discharge, undetermined intent, subsequent encounter	ICD10CM
Y24.0XXS	Airgun discharge, undetermined intent, sequela	ICD10CM
Y24.8	Other firearm discharge, undetermined intent	ICD10CM
Y24.8XXA	Other firearm discharge, undetermined intent, initial encounter	ICD10CM
Y24.8XXD	Other firearm discharge, undetermined intent, subsequent encounter	ICD10CM
Y24.8XXS	Other firearm discharge, undetermined intent, sequela	ICD10CM
Y24.9	Unspecified firearm discharge, undetermined intent	ICD10CM
Y24.9XXA	Unspecified firearm discharge, undetermined intent, initial encounter	ICD10CM
Y24.9XXD	Unspecified firearm discharge, undetermined intent, subsequent encounter	ICD10CM
Y24.9XXS	Unspecified firearm discharge, undetermined intent, sequela	ICD10CM
Y25	Contact with explosive material, undetermined intent	ICD10CM
Y25.XXXA	Contact with explosive material, undetermined intent, initial encounter	ICD10CM
Y25.XXXD	Contact with explosive material, undetermined intent, subsequent encounter	ICD10CM
Y25.XXXS	Contact with explosive material, undetermined intent, sequela	ICD10CM
Y26	Exposure to smoke, fire and flames, undetermined intent	ICD10CM

© 2021 Walsh CG et al. *JAMA Network Open.*

Y26.XXXA	Exposure to smoke, fire and flames, undetermined intent, initial encounter	ICD10CM
Y26.XXXD	Exposure to smoke, fire and flames, undetermined intent, subsequent encounter	ICD10CM
Y26.XXXS	Exposure to smoke, fire and flames, undetermined intent, sequela	ICD10CM
Y27	Contact with steam, hot vapors and hot objects, undetermined intent	ICD10CM
Y27.0	Contact with steam and hot vapors, undetermined intent	ICD10CM
Y27.0XXA	Contact with steam and hot vapors, undetermined intent, initial encounter	ICD10CM
Y27.0XXD	Contact with steam and hot vapors, undetermined intent, subsequent encounter	ICD10CM
Y27.0XXS	Contact with steam and hot vapors, undetermined intent, sequela	ICD10CM
Y27.1	Contact with hot tap water, undetermined intent	ICD10CM
Y27.1XXA	Contact with hot tap water, undetermined intent, initial encounter	ICD10CM
Y27.1XXD	Contact with hot tap water, undetermined intent, subsequent encounter	ICD10CM
Y27.1XXS	Contact with hot tap water, undetermined intent, sequela	ICD10CM
Y27.2	Contact with hot fluids, undetermined intent	ICD10CM
Y27.2XXA	Contact with hot fluids, undetermined intent, initial encounter	ICD10CM
Y27.2XXD	Contact with hot fluids, undetermined intent, subsequent encounter	ICD10CM
Y27.2XXS	Contact with hot fluids, undetermined intent, sequela	ICD10CM
Y27.3	Contact with hot household appliance, undetermined intent	ICD10CM
Y27.3XXA	Contact with hot household appliance, undetermined intent, initial encounter	ICD10CM
Y27.3XXD	Contact with hot household appliance, undetermined intent, subsequent encounter	ICD10CM
Y27.3XXS	Contact with hot household appliance, undetermined intent, sequela	ICD10CM
Y27.8	Contact with other hot objects, undetermined intent	ICD10CM
Y27.8XXA	Contact with other hot objects, undetermined intent, initial encounter	ICD10CM

© 2021 Walsh CG et al. *JAMA Network Open.*

Y27.8XXD	Contact with other hot objects, undetermined intent, subsequent encounter	ICD10CM
Y27.8XXS	Contact with other hot objects, undetermined intent, sequela	ICD10CM
Y27.9	Contact with unspecified hot objects, undetermined intent	ICD10CM
Y27.9XXA	Contact with unspecified hot objects, undetermined intent, initial encounter	ICD10CM
Y27.9XXD	Contact with unspecified hot objects, undetermined intent, subsequent encounter	ICD10CM
Y27.9XXS	Contact with unspecified hot objects, undetermined intent, sequela	ICD10CM
Y28	Contact with sharp object, undetermined intent	ICD10CM
Y28.0	Contact with sharp glass, undetermined intent	ICD10CM
Y28.0XXA	Contact with sharp glass, undetermined intent, initial encounter	ICD10CM
Y28.0XXD	Contact with sharp glass, undetermined intent, subsequent encounter	ICD10CM
Y28.0XXS	Contact with sharp glass, undetermined intent, sequela	ICD10CM
Y28.1	Contact with knife, undetermined intent	ICD10CM
Y28.1XXA	Contact with knife, undetermined intent, initial encounter	ICD10CM
Y28.1XXD	Contact with knife, undetermined intent, subsequent encounter	ICD10CM
Y28.1XXS	Contact with knife, undetermined intent, sequela	ICD10CM
Y28.2	Contact with sword or dagger, undetermined intent	ICD10CM
Y28.2XXA	Contact with sword or dagger, undetermined intent, initial encounter	ICD10CM
Y28.2XXD	Contact with sword or dagger, undetermined intent, subsequent encounter	ICD10CM
Y28.2XXS	Contact with sword or dagger, undetermined intent, sequela	ICD10CM
Y28.8	Contact with other sharp object, undetermined intent	ICD10CM
Y28.8XXA	Contact with other sharp object, undetermined intent, initial encounter	ICD10CM
Y28.8XXD	Contact with other sharp object, undetermined intent, subsequent encounter	ICD10CM

© 2021 Walsh CG et al. *JAMA Network Open.*

**Y28.8XXS**	Contact with other sharp object, undetermined intent, sequela	ICD10CM
**Y28.9**	Contact with unspecified sharp object, undetermined intent	ICD10CM
**Y28.9XXA**	Contact with unspecified sharp object, undetermined intent, initial encounter	ICD10CM
**Y28.9XXD**	Contact with unspecified sharp object, undetermined intent, subsequent encounter	ICD10CM
**Y28.9XXS**	Contact with unspecified sharp object, undetermined intent, sequela	ICD10CM
**Y29**	Contact with blunt object, undetermined intent	ICD10CM
**Y29.XXXA**	Contact with blunt object, undetermined intent, initial encounter	ICD10CM
**Y29.XXXD**	Contact with blunt object, undetermined intent, subsequent encounter	ICD10CM
**Y29.XXXS**	Contact with blunt object, undetermined intent, sequela	ICD10CM
**Y30**	Falling, jumping or pushed from a high place, undetermined intent	ICD10CM
**Y30.XXXA**	Falling, jumping or pushed from a high place, undetermined intent, initial encounter	ICD10CM
**Y30.XXXD**	Falling, jumping or pushed from a high place, undetermined intent, subsequent encounter	ICD10CM
**Y30.XXXS**	Falling, jumping or pushed from a high place, undetermined intent, sequela	ICD10CM
**Y31**	Falling, lying or running before or into moving object, undetermined intent	ICD10CM
**Y31.XXXA**	Falling, lying or running before or into moving object, undetermined intent, initial encounter	ICD10CM
**Y31.XXXD**	Falling, lying or running before or into moving object, undetermined intent, subsequent encounter	ICD10CM
**Y31.XXXS**	Falling, lying or running before or into moving object, undetermined intent, sequela	ICD10CM
**Y32**	Crashing of motor vehicle, undetermined intent	ICD10CM
**Y32.XXXA**	Crashing of motor vehicle, undetermined intent, initial encounter	ICD10CM
**Y32.XXXD**	Crashing of motor vehicle, undetermined intent, subsequent encounter	ICD10CM
**Y32.XXXS**	Crashing of motor vehicle, undetermined intent, sequela	ICD10CM

© 2021 Walsh CG et al. *JAMA Network Open.*

Y33	Other specified events, undetermined intent	ICD10CM
Y33.q	Other specified events, undetermined intent	ICD10CM
Y33.XXXA	Other specified events, undetermined intent, initial encounter	ICD10CM
Y33.XXXD	Other specified events, undetermined intent, subsequent encounter	ICD10CM
Y33.XXXS	Other specified events, undetermined intent, sequela	ICD10CM
Z91.5	Personal history of self-harm	ICD10CM
E980	Poisoning by solid or liquid substances, undetermined whether accidentally or purposely inflicted	ICD9CM
E980.0	Poisoning by analgesics, antipyretics, and antirheumatics, undetermined whether accidentally or purposely inflicted	ICD9CM
E980.1	Poisoning by barbiturates, undetermined whether accidentally or purposely inflicted	ICD9CM
E980.2	Poisoning by other sedatives and hypnotics, undetermined whether accidentally or purposely inflicted	ICD9CM
E980.3	Poisoning by tranquilizers and other psychotropic agents, undetermined whether accidentally or purposely inflicted	ICD9CM
E980.4	Poisoning by other specified drugs and medicinal substances, undetermined whether accidentally or purposely inflicted	ICD9CM
E980.5	Poisoning by unspecified drug or medicinal substance, undetermined whether accidentally or purposely inflicted	ICD9CM
E980.6	Poisoning by corrosive and caustic substances, undetermined whether accidentally or purposely inflicted	ICD9CM
E980.7	Poisoning by agricultural and horticultural chemical and pharmaceutical preparations other than plant foods and fertilizers, undetermined whether accidentally or purposely inflicted	ICD9CM
E980.8	Poisoning by arsenic and its compounds, undetermined whether accidentally or purposely inflicted	ICD9CM
E980.9	Poisoning by other and unspecified solid and liquid substances, undetermined whether accidentally or purposely inflicted	ICD9CM
E981	Poisoning by gases in domestic use, undetermined whether accidentally or purposely inflicted	ICD9CM

© 2021 Walsh CG et al. *JAMA Network Open.*

**E981.0**	Poisoning by gas distributed by pipeline, undetermined whether accidentally or purposely inflicted	ICD9CM
**E981.1**	Poisoning by liquefied petroleum gas distributed in mobile containers, undetermined whether accidentally or purposely inflicted	ICD9CM
**E981.8**	Poisoning by other utility gas, undetermined whether accidentally or purposely inflicted	ICD9CM
**E982**	Poisoning by other gases, undetermined whether accidentally or purposely inflicted	ICD9CM
**E982.0**	Poisoning by motor vehicle exhaust gas, undetermined whether accidentally or purposely inflicted	ICD9CM
**E982.1**	Poisoning by other carbon monoxide, undetermined whether accidentally or purposely inflicted	ICD9CM
**E982.8**	Poisoning by other specified gases and vapors, undetermined whether accidentally or purposely inflicted	ICD9CM
**E982.9**	Poisoning by unspecified gases and vapors, undetermined whether accidentally or purposely inflicted	ICD9CM
**E983**	Hanging, strangulation, or suffocation, undetermined whether accidentally or purposely inflicted	ICD9CM
**E983.0**	Hanging, undetermined whether accidentally or purposely inflicted	ICD9CM
**E983.1**	Suffocation by plastic bag, undetermined whether accidentally or purposely inflicted	ICD9CM
**E983.8**	Strangulation or suffocation by other specified means, undetermined whether accidentally or purposely inflicted	ICD9CM
**E983.9**	Strangulation or suffocation by unspecified means, undetermined whether accidentally or purposely inflicted	ICD9CM
**E984**	Submersion (drowning), undetermined whether accidentally or purposely inflicted	ICD9CM
**E985**	Injury by firearms, air guns and explosives, undetermined whether accidentally or purposely inflicted	ICD9CM
**E985.0**	Injury by handgun, undetermined whether accidentally or purposely inflicted	ICD9CM
**E985.1**	Injury by shotgun, undetermined whether accidentally or purposely inflicted	ICD9CM
**E985.2**	Injury by hunting rifle, undetermined whether accidentally or purposely inflicted	ICD9CM

© 2021 Walsh CG et al. *JAMA Network Open.*

**E985.3**	Injury by military firearms, undetermined whether accidentally or purposely inflicted	ICD9CM
**E985.4**	Injury by other and unspecified firearm, undetermined whether accidentally or purposely inflicted	ICD9CM
**E985.5**	Injury by explosives, undetermined whether accidentally or purposely inflicted	ICD9CM
**E985.6**	Injury by air gun, undetermined whether accidental or purposely inflicted	ICD9CM
**E985.7**	Injury by paintball gun, undetermined whether accidental or purposely inflicted	ICD9CM
**E986**	Injury by cutting and piercing instruments, undetermined whether accidentally or purposely inflicted	ICD9CM
**E987**	Falling from high place, undetermined whether accidentally or purposely inflicted	ICD9CM
**E987.0**	Falling from residential premises, undetermined whether accidentally or purposely inflicted	ICD9CM
**E987.1**	Falling from other man-made structures, undetermined whether accidentally or purposely inflicted	ICD9CM
**E987.2**	Falling from natural sites, undetermined whether accidentally or purposely inflicted	ICD9CM
**E987.9**	Falling from unspecified site, undetermined whether accidentally or purposely inflicted	ICD9CM
**E988**	Injury by other and unspecified means, undetermined whether accidentally or purposely inflicted	ICD9CM
**E988.0**	Injury by jumping or lying before moving object, undetermined whether accidentally or purposely inflicted	ICD9CM
**E988.1**	Injury by burns or fire, undetermined whether accidentally or purposely inflicted	ICD9CM
**E988.2**	Injury by scald, undetermined whether accidentally or purposely inflicted	ICD9CM
**E988.3**	Injury by extremes of cold, undetermined whether accidentally or purposely inflicted	ICD9CM
**E988.4**	Injury by electrocution, undetermined whether accidentally or purposely inflicted	ICD9CM
**E988.5**	Injury by crashing of motor vehicle, undetermined whether accidentally or purposely inflicted	ICD9CM
**E988.6**	Injury by crashing of aircraft, undetermined whether accidentally or purposely inflicted	ICD9CM

© 2021 Walsh CG et al. *JAMA Network Open.*

**E988.7**	Injury by caustic substances, except poisoning, undetermined whether accidentally or purposely inflicted	ICD9CM
**E988.8**	Injury by other specified means, undetermined whether accidentally or purposely inflicted	ICD9CM
**E988.9**	Injury by unspecified means, undetermined whether accidentally or purposely inflicted	ICD9CM
**E989**	Late effects of injury, undetermined whether accidentally or purposely inflicted	ICD9CM
**V62.84**	Suicidal ideation	ICD9CM

© 2021 Walsh CG et al. *JAMA Network Open.*
